# The interactive effects of extreme temperatures and PM_2.5_ pollution on mortalities in Jiangsu Province, China

**DOI:** 10.1038/s41598-023-36635-x

**Published:** 2023-06-10

**Authors:** Lian Zhou, Yuning Wang, Qingqing Wang, Zhen Ding, Hui Jin, Ting Zhang, Baoli Zhu

**Affiliations:** 1 Center for Disease Control and Prevention of Jiangsu Province, Nanjing, 210009 China; 2 Department of Epidemiology and Health Statistics, School of Public Health, Southeast University, No. 87 Dingjia Bridge, Gulou District, Nanjing, 210009 China; 3 Key Laboratory of Environmental Medicine Engineering, Ministry of Education, School of Public Health, Southeast University, Nanjing, 210009 China; 4 State Key Laboratory of Pollution Control and Resource Reuse, School of the Environment, Nanjing University, Nanjing, 210023 China; 5 Department of Civil, Environmental, and Infrastructure Engineering, George Mason University, Fairfax, VA 22030 USA; 6 Center for Global Health, School of Public Health, Nanjing Medical University, Nanjing, 211166 China

**Keywords:** Environmental impact, Environmental social sciences, Public health

## Abstract

Exposure to extreme temperatures or fine particles is associated with adverse health outcomes but their interactive effects remain unclear. We aimed to explore the interactions of extreme temperatures and PM_2.5_ pollution on mortalities. Based on the daily mortality data collected during 2015–2019 in Jiangsu Province, China, we conducted generalized linear models with distributed lag non-linear model to estimate the regional-level effects of cold/hot extremes and PM_2.5_ pollution. The relative excess risk due to interaction (RERI) was evaluated to represent the interaction. The relative risks (RRs) and cumulative relative risks (CRRs) of total and cause-specific mortalities associated with hot extremes were significantly stronger (*p* < 0.05) than those related to cold extremes across Jiangsu. We identified significantly higher interactions between hot extremes and PM_2.5_ pollution, with the RERI range of 0.00–1.15. The interactions peaked on ischaemic heart disease (RERI = 1.13 [95%CI: 0.85, 1.41]) in middle Jiangsu. For respiratory mortality, RERIs were higher in females and the less educated. The interaction pattern remained consistent when defining the extremes/pollution with different thresholds. This study provides a comprehensive picture of the interactions between extreme temperatures and PM_2.5_ pollution on total and cause-specific mortalities. The projected interactions call for public health actions to face the twin challenges, especially the co-appearance of hot extremes and PM pollution.

## Introduction

Extreme temperatures represent one of the most severe environmental challenges. Even though cold extremes have become less frequent and severe in recent decades^1^, low temperature has been added as a new risk factor for premature deaths according to the Global Burden of Disease report since 2019^2^. With the process of global climate change, hot extremes have become more frequent and intense across most regions^1^. In 2022, more than half of mainland China faced an extreme heat event lasting for more than 30 days, with the highest temperature reaching over 44 ℃^3^. Studies examining the impacts of air pollutants across different temperature intervals demonstrated a significantly higher health burden from extreme temperatures than that from the moderate temperature categories^4^. The hot-related burden is more notable in countries with less favorable socio-demographic conditions^5^.

In spite of the decreasing air pollution level^6,7^, fine particulate matter (PM_2.5_) is still related to the largest amount of premature death worldwide^8^. Considerable consistency existed across studies that short/long-term exposure to PM_2.5_ is related to an increment in acute cardiovascular and respiratory diseases and mortalities^9,10^. The situation worsens in China, where the number of PM_2.5_-related deaths reached 0.85 million in 2017, accounting for 29% of that globally^11^.

Studies regarding individual effects of extreme temperatures have been widely carried out, where air pollutant(s) is used as a confounder^12–14^, and vice versa^15,16^. Emerging evidence of the interactions between extreme temperatures and air pollutants was also observed. Various studies conducted in China and the United States have explored the interactive effect between extreme temperatures and the main air pollutants (such as PM_2.5_, PM_10_, SO_2_, and NO_2_) on preterm birth, total and cause-specific mortalities, and detected positive interactions of cold spells and heat waves with air pollutants on respiratory and circulatory disease mortalities^17–24^. Studies that focused on the interactions of the Air Quality Index and extreme temperatures in China, however, indicated conflicting interactive effects on respiratory disease hospitalization and mortality^25,26^. Other studies conducted in countries/areas such as South Asia, Northeast Asia, South Korea, and Taiwan reported aggravated effects on respiratory disease morbidity, cardiovascular disease mortality, and preterm birth of the extreme temperatures as the pollution increased^27–30^. Studies carried out in European cities and Brazil showed similar synergistic effects between heat and air pollutants on total, respiratory, and cardiac disease mortalities^31–33^.

The simultaneous exposure to extreme temperatures and PM_2.5_ may also lead to exacerbation of adverse health effects due to interaction, but the evidence regarding which was still scarce. Positive interactions between cold spells and PM_2.5_ were on preterm birth and non-accidental mortality^29,34^. More literature discussed the interactions between hot extremes and PM_2.5_. Yitshak-Sade et al.^31^ disclosed that the short-term PM_2.5_ effect on respiratory admissions was larger on warmer days. Wang et al.^56^ also pointed out the existence of synergistic effects of heatwaves and PM_2.5_ exposure on preterm birth. However, these studies (1) still focused on the individual effects of temperature and PM_2.5_ without quantifying the interactive effects; or (2) explored the interactions between cold/hot extremes and PM_2.5_ only.

Jiangsu Province is one of the most developed areas in China that is also experiencing severe PM_2.5_ pollution^35^. Locating in the transition belt from a subtropical to temperate zone with a typical East Asia monsoon climate^36^, Jiangsu has distinct weather conditions between seasons. Based on the high-quality cause-specific mortality data collected from 2015 to 2019 in Jiangsu, we aimed to investigate the interactive effects of extreme temperatures and PM_2.5_ pollution, with a focus on the difference in the interactions between cold/hot extremes and PM_2.5_ pollution.

## Materials and methods

### Data collection

We extracted the city-level daily cause-specific mortality records from January 2015 to December 2019 across Jiangsu Province from the Center for Disease Control (CDC) and Prevention of Jiangsu Province. The mortality records were managed by the Jiangsu Provincial Mortality Surveillance System, the data of which was reported and examined by professionals such as doctors, local CDC staff, or other health workers. The underlying cause of death was classified following the 10th Revision of the International Classification of Disease (ICD10), including total causes, non-accidental causes (A00-R99), respiratory disease (RESP; J00-J99), chronic obstructive pulmonary disease (COPD; J41-J44), cardiovascular diseases (CVD; I00-I99), stroke (I60-I69), myocardial infarction (MI; I21-I23), and ischaemic heart disease (IHD; I20-I25).

**Figure 1:**
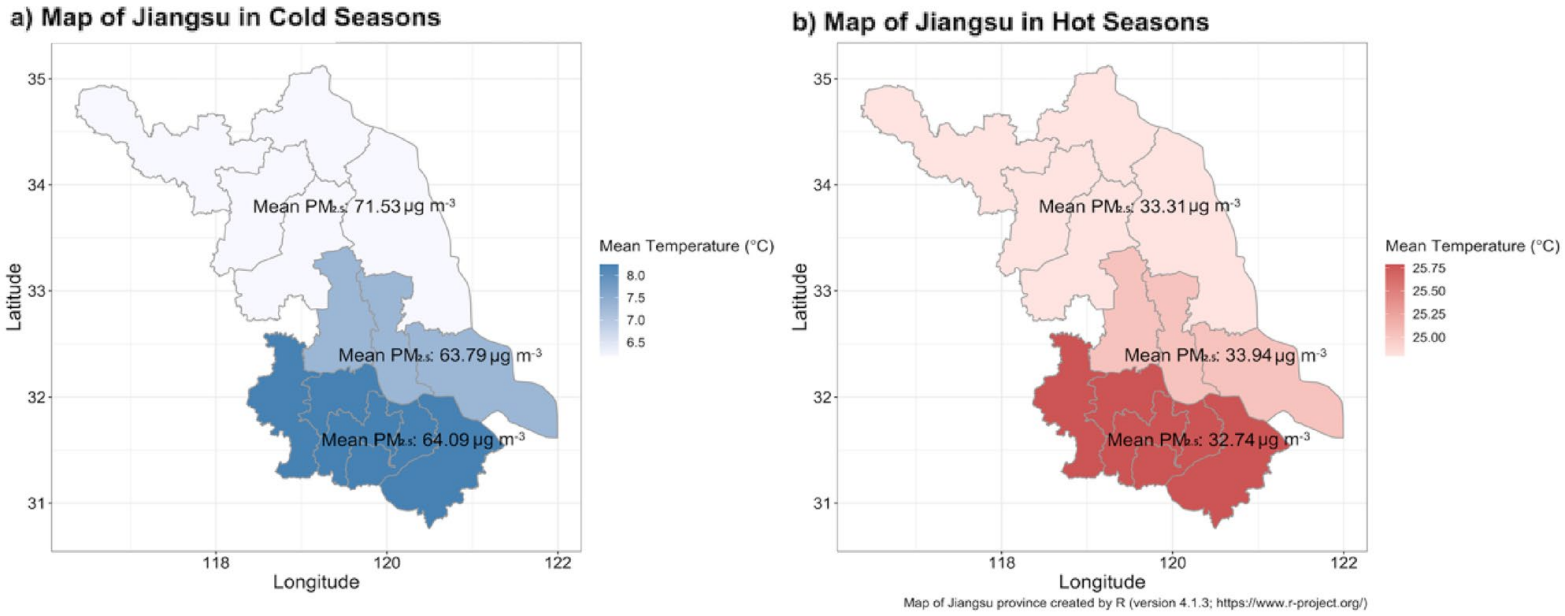
The distribution of extreme temperatures and PM_2.5_ pollution events in northern, middle, and southern Jiangsu, 2015–2019.

City-level daily mean temperature and relative humidity data were obtained from the China Meteorological Data Sharing Service System. Daily average PM_2.5_ concentrations were collected from the Jiangsu Environmental Monitoring Center. Cities in Jiangsu Province were divided into three parts—northern (Lianyungang, Xuzhou, Suqian, Huaian, Yancheng), middle (Yangzhou, Taizhou, Nantong), and southern (Nanjing, Suzhou, Wuxi, Changzhou, Zhenjiang) Jiangsu—according to their geographical locations. The geographical location of the study site is shown in Fig. 1, which was created by the ‘*ggplot2*’ package in R software (version 4.1.3; https://www.r-project.org/).

### Extreme temperature and PM_2.5_ pollution definition

We set cold seasons from November to March and hot seasons from May to September^37,38^. Without a standard definition for heatwave or cold spell^39,40^, we defined a hot or cold extreme as the day when the daily average temperature was lower than the 5th percentile in the cold or higher than the 95th percentile in the hot seasons of a given area. We categorized a day with the PM_2.5_ concentration higher than 35 μg m^−3^ as a PM_2.5_ pollution day following the air quality standard in China (GB 3095—2012).

### Statistical model

#### Independent effects of temperature and PM_2.5_

A generalized linear model (GLM) of Poisson distribution was used to estimate the independent effects of extreme temperature and PM_2.5_ pollution.

1 $$\begin{aligned} logE\left[ {Y_{t}^{c} } \right] & = \beta_{0} + ET_{t} + AP_{t} + ns\left( {relative \,\,humidity, df = 3} \right) \\ & \;\;\; + factor\left( {Holiday} \right) + year \\ \end{aligned}$$

where *ET*_*t*_ and *AP*_*t*_ refer to whether the day *t* is an extreme temperature or PM_2.5_ pollution day. Both *ET*_*t*_ and *AP*_*t*_ are binary variables (yes/no). ${Y}_{t}^{c}$ is the daily death number from total, non-accidental, or cause-specific disease for region *c* on day *t*. A natural cubic spline with a degree of freedom (*df*) of 3 was used to control for the potential nonlinearity of relative humidity, the *df* value was determined according to the Akaike Information Criterion (AIC) analyses; *Holiday* is a binary variable that represents the public holidays; $year$ is a numeric variable that was used to control long-term trends. We used the relative risk (RR) to represent the extreme temperature- or PM_2.5_-related risk of mortality.

#### Lag effects of temperature and PM_2.5_

A Poisson GLM in combination with a distributed lag non-linear model (DLNM) was applied to estimate the independent and interactive cumulative lag effects of extreme temperature and PM_2.5_ pollution.

2 $$\begin{aligned} logE\left[ {Y_{t}^{c} } \right] & = \beta_{0} + cb\left( {COMB_{t} , lag} \right) + ns\left( {mean \,\,temperature, df = 3} \right) \\ & \;\;\; + ns\left( {PM_{2.5} , df = 3} \right) + ns\left( {relative \,\, humidity, df = 3} \right) \\ & \;\;\; + factor\left( {Holiday} \right) + year \\ \end{aligned}$$

where *COMB*_*t*_ is a binary variable (yes/no) that refers to whether the day *t* is a day with combined events of extreme temperature and PM_2.5_ pollution; *cb*(.) means cross-basis function obtained by applying the DLNM to *COMB*_*t*_, with a strata function and a natural cubic spline function (NS) with 4 *dfs* for exposure- and lag-response dimensions. To account for the detected chronicity of cold effects and acuteness of heat events, we set the maximum lag in cold seasons at 27 days and for hot seasons at 7 days^17,40,41^. ${Y}_{t}^{c}$ is the daily death number from total, non-accidental, or cause-specific disease for region *c* on day *t*. A natural cubic spline with a *df* of 3 was used to control for the potential nonlinearity of relative humidity, mean temperature, and PM_2.5_ concentration, where the *df* value was determined according to the AIC analyses. We used the cumulative relative risk (CRR) to represent the combined-related risk of mortality. The estimated CRRs of extreme temperature events and PM_2.5_ pollution was also investigated.

#### The interactive effect of extreme temperatures and PM_2.5_ pollution

We investigated the additive interactions by calculating the relative excess risk because of interaction (RERI). Three RRs—including the risk when both extreme temperatures and PM_2.5_ pollution occurred (RR_11_), extreme cold or hot events only (RR_10_), and PM_2.5_ pollution only (RR_01_)—were used to calculate the RERI through the equation^42,43^:

3 $${\text{RERI}}\; = \;\left( {{\text{RR}}_{{{11}}} {-}{ 1}} \right)\; - \;\left( {{\text{RR}}_{{{1}0}} {-}{ 1}} \right)\; - \;\left( {{\text{RR}}_{{0{1}}} {-}{ 1}} \right) .$$

The RERIs of PM_2.5_ pollution and cold or hot extremes were calculated separately. We estimated the RERIs for total and cause-specific mortality and for different subgroups.

To identify the more susceptible groups, we conducted subgroup analyses stratified by gender, age (< 64, 64– 74, > 74), and education level (middle school and lower, high school, and college).

#### Sensitivity analysis

We carried out a series of sensitivity analyses to test the robustness of the results. First, we applied different thresholds of extreme temperature events (the 2.5th and 97.5th percentile of temperature in the study period) and PM_2.5_ pollution (the 75th and 50th percentile of PM_2.5_ concentration in the study period; Table S1). Secondly, we substituted ‘day of week’ (*DOW*) and calendar day for ‘*Holiday*’ and ‘*year*’ to account for short- and long-term trends. A natural cubic spline smooth function was applied to the calendar day and the *df* was determined based on the AIC analysis. Thirdly, we used PM_2.5_ concentration instead of the dichotomous variable indicating whether the day is a PM_2.5_ pollution day in independent effect models to examine the PM_2.5_-related relative risk of mortality.

We carried out all analyses at city and region levels with R software (version 4.1.3). Two-tailed *P* values less than 0.05 were considered statistically significant for all statistical tests in this study.

## Results

### Descriptive statistics

During the study period, the daily number of deaths due to non-accidental and respiratory disease was the highest in middle Jiangsu and the lowest in southern Jiangsu (Table 1), while the daily number of deaths from CVD and stroke was the highest in northern Jiangsu and the lowest in southern Jiangsu. When selecting the cutoffs with the 5th percentile of average daily temperatures in cold seasons and 95th percentile in hot seasons, the highest cutoff appeared in southern Jiangsu (0.9 and 32.4 ℃ in cold and hot seasons, respectively; Fig. 1). The number of PM_2.5_ pollution days (> 35 μg m^−3^) peaked in northern Jiangsu in cold seasons (18.6%), but in middle Jiangsu in hot seasons (60.4%). The cold and hot extremes distribution showed different time trends, with the highest number of cold extreme days in 2018 and the highest number of hot extreme days in 2017. The detected PM_2.5_ pollution days per year decreased in three regions at large from 2015 to 2019 (Fig. S1).

**Table 1:** Descriptive statistics on the study population, study period, daily non-accidental mortality, temperature, and PM_2.5_ in Jiangsu Province, 2015–2019.

Region	Daily mean death numbers								Daily mean temperature (℃)		Corresponding percentile of PM_2.5_: 35 μg m^−3^ (%)		
	Total	Non-accidental	RESP	CVD	Stroke	COPD	MI	IHD	5th percentile in study period	95th percentile in study period	Cold season	Hot season	Whole year
Northern Jiangsu	117.9	108.8	15.7	48.7	27.3	13.5	11.6	16.6	− 1.2	30.8	18.6	61.9	39.3
Middle Jiangsu	128.1	118.6	16.2	46.2	26.5	13.7	6.7	13.4	0.0	31.5	23.3	60.4	41.5
Southern Jiangsu	94.5	86.3	9.7	32.6	19.8	6.2	3.1	7.8	0.9	32.4	20.9	62.3	41.2

### Independent effect of extreme temperatures

**Figure 2:**
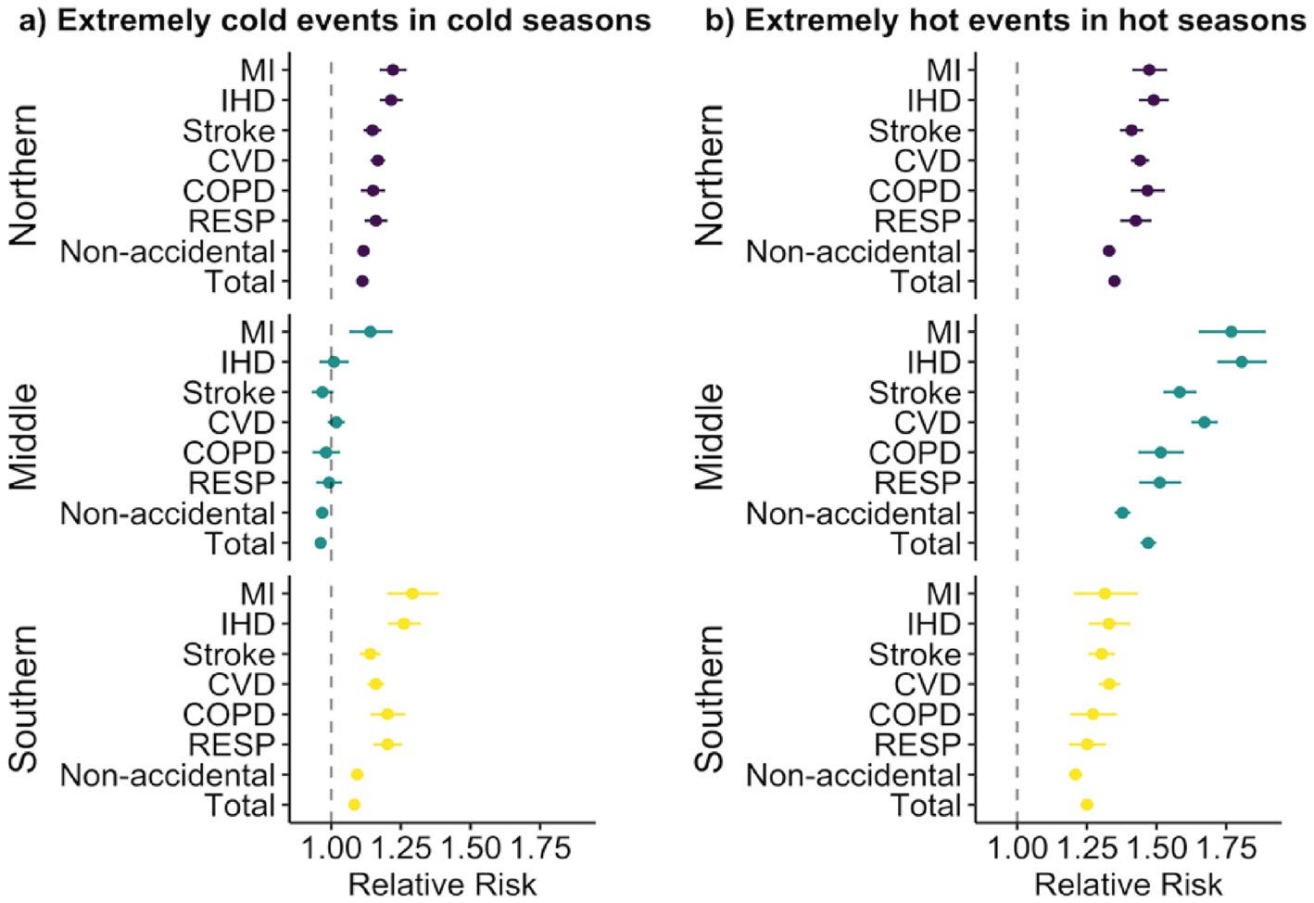
The relative risks (RR) of total and cause-specific mortalities associated with cold (**a**) or hot (**b**) extremes in three regions in Jiangsu Province in 2015–2019.

The association between cause-specific mortalities and cold extremes differed in regions, with the RRs ranging from 0.96 to 1.30 (Fig. 2a; Table S2). Strongly pronounced cold effects for southern and northern Jiangsu were on total and cause-specific mortalities. In comparison, in middle Jiangsu, the only significant RR was discovered in MI (RR = 1.14 [95%CI: 1.07, 1.22]), but still lower than that in two other regions. The RRs of respiratory disease and CVD mortality in southern Jiangsu were slightly higher than those in the northern part. At the city level, a higher risk of cause-specific mortality was also associated with cold extremes (Table S3).

Hot extremes related-mortality risks varied in the three regions in Jiangsu, with RRs significantly higher than 1.0 (*p* < 0.05) (Fig. 2b; Table S2). Heat effects were more pronounced in middle Jiangsu than in the two other regions. The RR of IHD peaked among all diseases, with the value being 1.33 (95%CI: 1.26, 1.40), 1.49 (95%CI: 1.44, 1.54), and 1.81 (95%CI: 1.72, 1.89) in the in southern, northern, and middle Jiangsu, respectively. Similarly, RRs of cause-specific mortalities were significantly higher than 1.0 (*p* < 0.05) under hot extremes at the city level (Table S3).

### Independent effect of PM_2.5_ pollution

**Figure 3:**
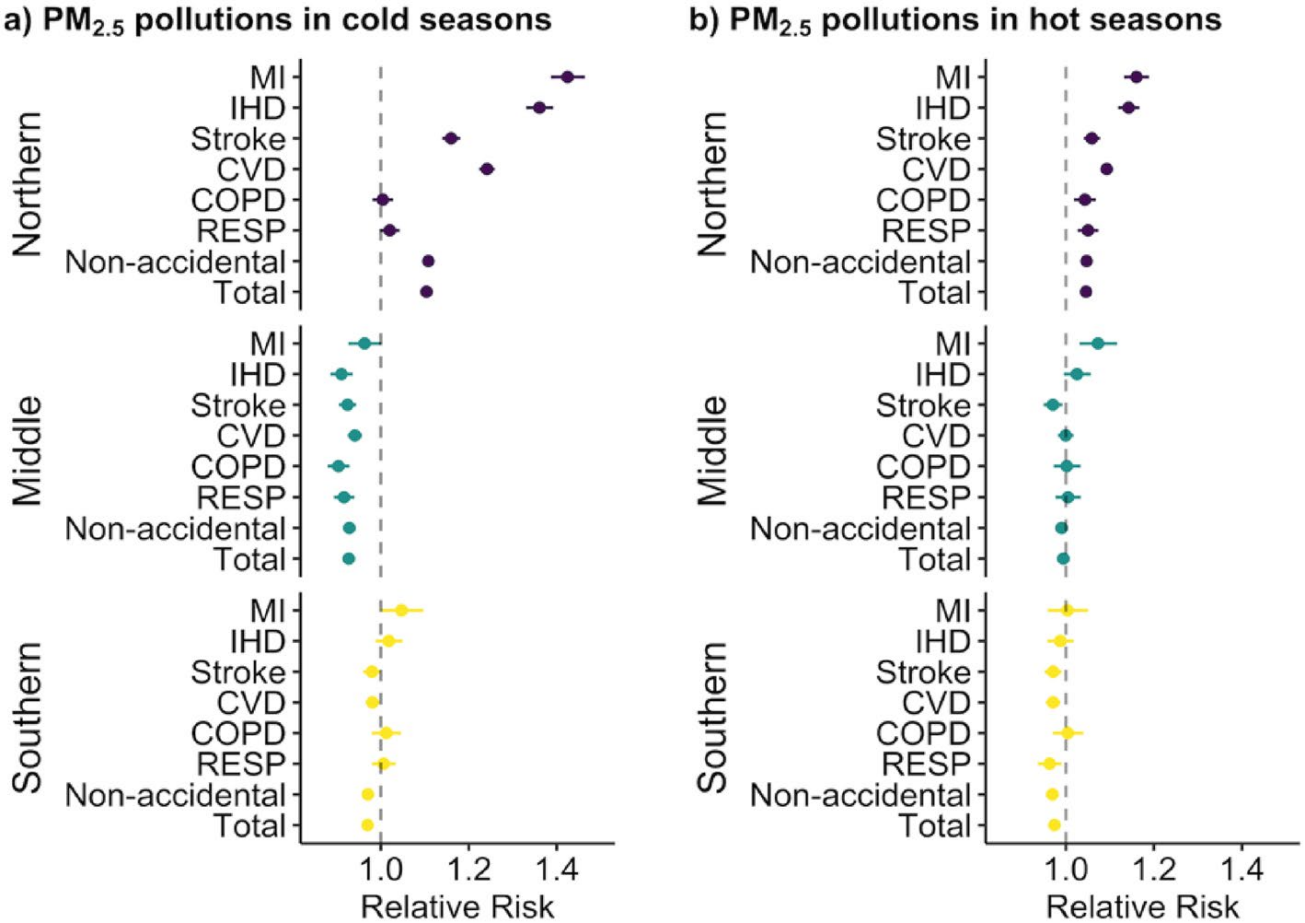
The relative risk (RR) of total and cause-specific mortalities associated with PM_2.5_ pollution (> 35 μg m^−3^) events in the cold (**a**) and hot (**b**) seasons at region levels in Jiangsu Province during 2015–2019.

The PM_2.5_ pollution associated cause-specific mortality RRs were lower (*p* > 0.05) in cold seasons (median = 0.99 [range: 0.90, 1.50]) than in hot seasons (median = 1.00 [range: 0.96, 1.20]; Fig. 3a and b). During cold seasons, the most pronounced effect of PM_2.5_ pollution was discovered on MI for all three regions in Jiangsu Province. In hot seasons, the RR of MI mortality remained the highest in northern and middle Jiangsu, but the highest RR in southern Jiangsu was due to COPD (RR = 1.00 [95%CI: 0.97, 1.04]; Table S2). The PM_2.5_ pollution-related risk of respiratory mortality was below 1.0 in middle and southern Jiangsu in cold and hot seasons, respectively. However, at the city level, the RR of respiratory mortality associated with PM_2.5_ pollution was higher than 1.0 in both hot and cold seasons (Table S3).

### Interactive effect of extreme temperatures and PM_2.5_ pollution

**Figure 4:**
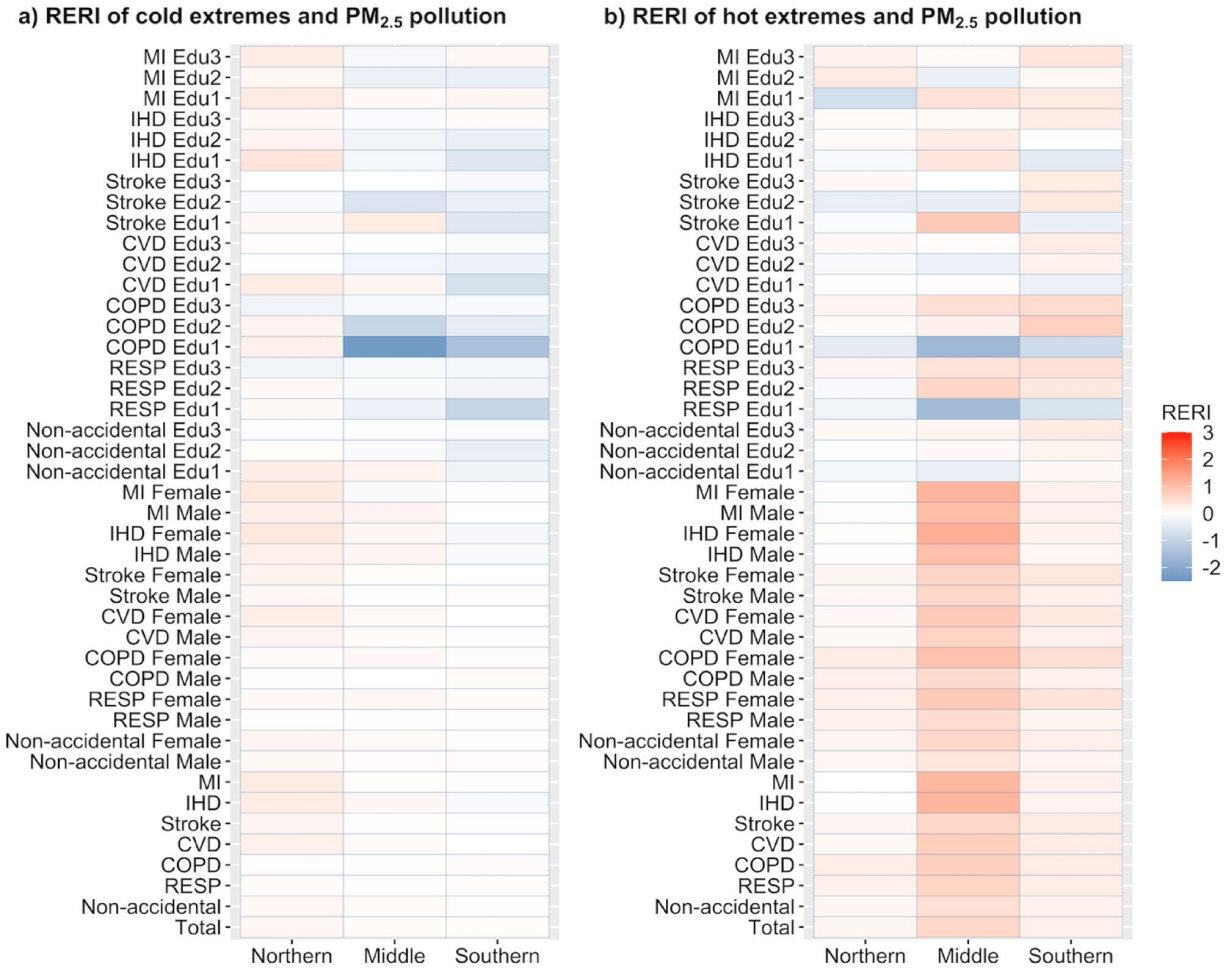
The relative excess risk due to interaction (RERI) of cold/hot extremes (< 5th/> 95th percentile) and PM_2.5_ pollution (> 35 μg m^−3^) on mortalities for different subgroups in the cold (**a**) and hot (**b**) seasons in Jiangsu Province during 2015–2019. *Edu1, college; Edu2, high school; Edu3, middle school and lower.

Figure 4a displayed the joint effects of extreme cold events and PM_2.5_ pollution on total and non-accidental (including cause-specific) mortalities. The estimated RERIs in cold seasons ranged between − 0.14 and 0.3. Positive interactions of cold extremes and PM_2.5_ pollution were detected in northern and middle Jiangsu on cause-specific mortalities; the interactions were negative on CVD, IHD, and MI in southern Jiangsu (Table S4). For total and non-accidental mortality, joint effects were discovered in northern, middle, and southern Jiangsu, with the highest RERI in northern Jiangsu (RERI = 0.12, [95%CI: − 0.04, 0.27]). In northern and middle Jiangsu, the joint effect was the strongest on IHD (RERI = 0.30 [95%CI: − 0.02, 0.62] and RERI = 0.12 [95%CI: − 0.09, 0.33], respectively) among all cause-specific mortalities. The interaction on respiratory disease mortality was insignificant in all three regions during cold seasons.

Positive interactive effects were also discovered between hot extremes and PM_2.5_ pollution on total and cause-specific mortalities in the three regions of Jiangsu Province, with the estimated RERIs between 0.00 and 1.15 (Fig. 4b, Table S4). Significant joint effects (*p* < 0.05) were identified in total and non-accidental mortalities in all three regions in Jiangsu. In hot seasons, the effects were the most notable for interactions on cause-specific mortalities in middle Jiangsu, followed by those in southern and northern Jiangsu. The interactions peaked on IHD (RERI = 1.13 [95%CI: 0.85, 1.41]) in middle Jiangsu. The RERIs for respiratory and cardiovascular mortalities were generally significant, with *p* < 0.05. The estimated RERIs in southern and middle Jiangsu were generally 0.16–1.10 higher in hot seasons than those in cold seasons (Table S4). However, in northern Jiangsu, the RERIs of CVD, Stroke, IHD, and MI were lower during hot seasons than cold seasons, and the differences in RERIs were lower than that in the two other regions. At the city level, interactions were also higher in hot seasons than in cold seasons, except for Xuzhou, a city located in northern Jiangsu (Table S5).

In the stratified analysis, joint effects on respiratory disease and COPD mortality were more robust in females in all three regions in Jiangsu during both hot and cold seasons (Fig. 4a and b). In cold seasons, higher (*p* > 0.05) joint effects were on non-accidental, CVD, MI, and stroke mortalities in the college education group in northern and middle Jiangsu. However, negative interactions were stronger (*p* > 0.05) on respiratory disease and COPD mortalities in the college education group in middle and southern Jiangsu (Fig. 4a). During hot seasons, more potent joint effects were in the middle school and lower education group on respiratory disease mortality with RERIs of 0.17 (95% CI: 0.03, 0.31), 0.42 (95% CI: 0.21, 0.64), and 0.47 (95% CI: 0.31, 0.63) in northern, middle, and southern Jiangsu, respectively. The synergistic effect on non-accidental mortality was the strongest in the oldest age group (> 74) in hot seasons (Fig. S2).

**Figure 5:**
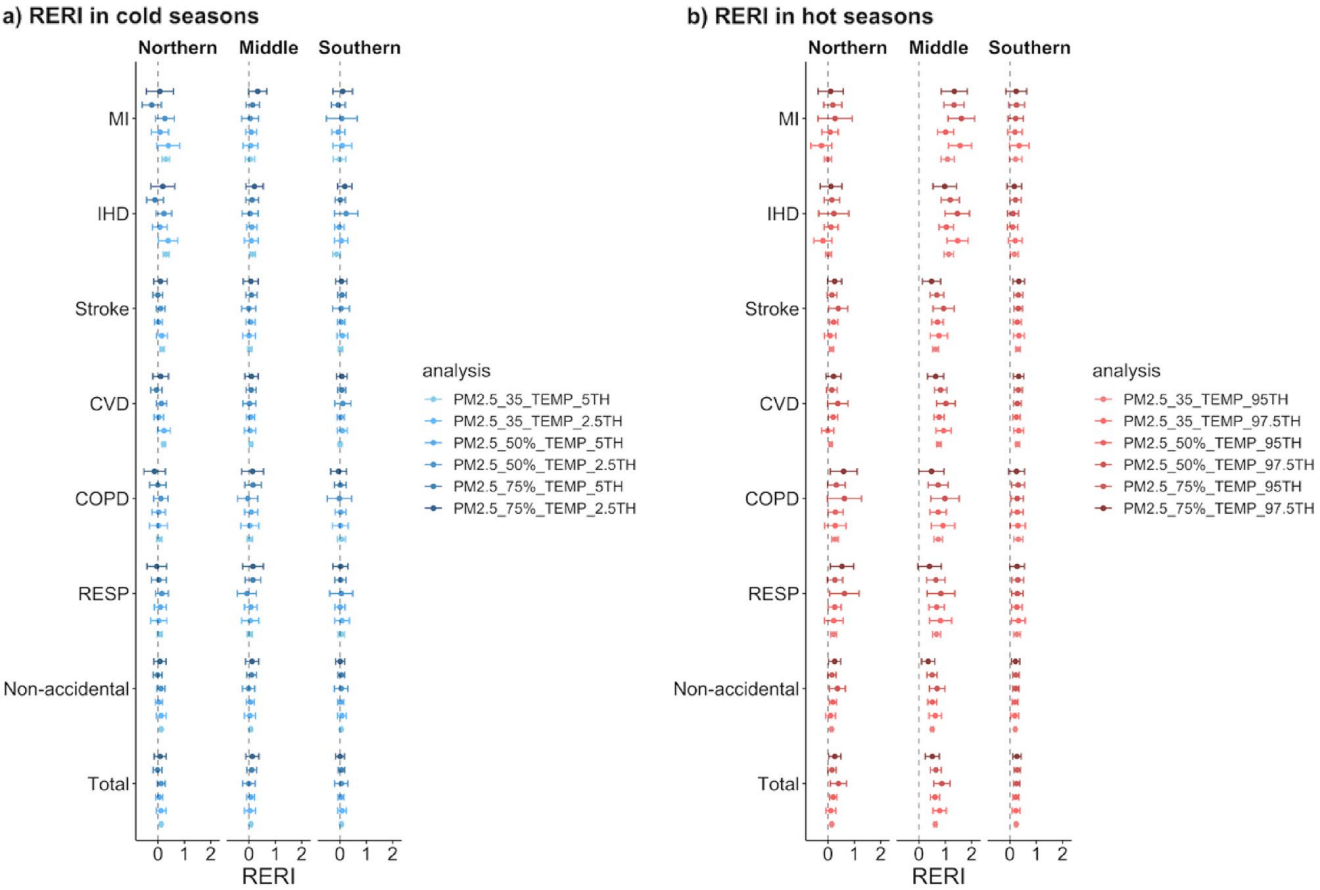
Sensitivity analyses results of RERIs on mortalities under different thresholds of cold/hot extremes and PM_2.5_ pollution in the cold (**a**) and hot (**b**) seasons in Jiangsu Province during 2015–2019.

The interaction effects were robust when using ‘*DOW*’ and calendar day to account for the short-/long-term trends (Tables S6, S7). Similarly, with the same threshold of PM_2.5_ pollution, the interactive effects persisted when changing the thresholds for cold/hot extremes (Fig. 5; Table S8). Specially, in northern and southern Jiangsu, the interactive effects on respiratory and cardiovascular mortality increased as the temperature thresholds became stricter (higher cutoffs for hot extremes and lower cutoffs for cold extremes). Instead, with given extreme temperature thresholds, the more positive interactions on respiratory and cardiovascular mortalities were associated with less intense PM_2.5_ pollution thresholds in northern and middle Jiangsu in hot seasons.

### Lag effect of the combined events, extreme temperatures, and PM_2.5_ pollution

The cumulative relative risks (CRRs) of combined events were lower in cold seasons (median = 1.87 [range: 0.84, 3.95]) than in hot seasons (median = 3.22 [range: 1.36, 8.63]). Compared with the RRs of combined events, the CRRs were higher (*p* > 0.05) in cold seasons in total and cause-specifically mortalities except for stroke in middle Jiangsu (Fig. S3). The RR of combined events on total mortality varied as the lag time increased in both cold and hot seasons, with higher RRs during hot than cold seasons on lag days (Fig. S4). During hot seasons, significantly higher (*p* < 0.05) CRRs were on all mortalities than RRs. The CRRs of extreme temperatures and PM_2.5_ pollution at lag 0–27 and lag 0–7 days in cold and hot seasons showed similar distributions as RRs in three regions of Jiangsu on total and cause-specific mortalities (Figs. S5 and S6).

## Discussion

To the best of our knowledge, this is the first study to provide quantitative assessments and important insights regarding the interactions between hot and cold extremes and air pollution on public health. We focused on the effects of interactions at the regional level because of the similarities in climate, demographical, and socioeconomic status among cities in the specific region, so that the results can be applied to a larger spatial. We detected that exposure to PM_2.5_ pollution and extreme temperatures were associated with excess risk of total and cause-specific mortalities due to interaction. The interactions between PM_2.5_ pollution and cold extremes were mainly positive but less significant. However, significant synergistic effects were detected between PM_2.5_ pollution and hot extremes. Interactions were more robust in respiratory than cardiovascular mortalities across Jiangsu Province.

We revealed similar adverse health effects of extreme temperatures on mortality as existing studies^44–47^. The detected independent acute effects were stronger and more robust in hot seasons. The trends of independent cumulative lag effects were similar as acute effects. Fast urbanization, large populations, and the dramatically changing environment have made China one of the most vulnerable regions experiencing frequent hot extremes^48–50^. It is also plausible that high temperatures tend to trigger more acute health effects while cold events are associated with more chronic influence^14,51^. In contrast with previous studies^52,53^, at the regional level, PM_2.5_ pollution might not always increase the respiratory mortality risk in Jiangsu. However, PM_2.5_ pollution was associated with higher city-level respiratory mortality risks in Jiangsu. This difference may be due to local characteristics at a smaller scale spatial scale, which needs further exploration.

Our results go beyond previous studies by disclosing the notable difference in interactions between hot and cold extremes with PM_2.5_ pollution^39,54,55^. The lag effects of combined events and the synergistic effect were stronger and more robust in hot seasons. Similarly, the excess risk of preterm birth due to interactions between PM_2.5_ and extreme temperatures ranged from 0.10 to 2.45 at high temperatures, higher than that detected at cold temperatures^56,57^. Even though the PM_2.5_ level tends to be more hazardous during cold seasons than hot seasons^58^, people are also less likely to take outdoor physical activity and more inclined to take actions to reduce indoor PM_2.5_ levels when the PM_2.5_ concentration becomes more hazardous^59–61^. Thus, personal PM_2.5_ exposures in cold seasons might be much lower than ambient levels in hot seasons. The uptake of PM_2.5_ may further increase through raised skin blood flow, minute ventilation, and sweating in hot seasons^58^. The toxicity of PM_2.5_ also increases as the temperature elevates^58^. Together, the relatively increased uptake and elevated toxicity of PM_2.5_ aggregate the interactions between PM_2.5_ and hot extremes. With global climate change, the co-occurrence of heatwaves and extreme PM_2.5_ pollution events are expected to increase by 175% in frequency^27^. Causes and solutions to climate change and air pollution are closely linked^62^. Internationally coordinated strategies regarding environmental challenges are warranted for global health benefits.

The synergistic effects on respiratory mortality in hot and cold seasons were consistent across regions; spatial heterogeneity exists in the interactive effects on cardiovascular mortality. The three regions in Jiangsu had higher interactions on respiratory mortality in hot seasons than in cold seasons. Similarly, a stronger synergistic effect of PM_2.5_ on respiratory disease was reported in hot seasons in Beijing and New England^20,31^. However, the studies in Chengdu and Hong Kong suggested an opposite trend^54,63^, which might be related to the special local geological and meteorological conditions. Regional differences exist in the interactions on respiratory mortality. For cardiovascular mortality, the disparities in interactions between regions may be explained by the environmental inequalities that could lead to disproportionate exposure to air pollutants and extreme temperatures for people in higher-middle income regions in China^64^, which differs from the U.S.^65^. The high-ranked average daily temperatures, PM_2.5_ concentrations, vehicle ownership, population density, and gross domestic product in middle Jiangsu may explain the highest synergistic effects on cardiovascular mortality in hot seasons. Regarding the spatial heterogeneity in the interactions, regional-level early warning systems for the occurrence of PM_2.5_ pollution and extreme temperatures are suggested to protect populations from the potential effects in identified areas.

Our estimates bear out the suggestions that the less educated and females may be more vulnerable to joint exposure to extreme temperatures and PM_2.5_ pollution. The association between cause-specific mortalities and high temperatures was more profound in the less educated^66,67^. The Air pollutants-related respiratory mortality risk was also higher in women than in men under both hot and cold extremes^19,68,69^. Lower education levels are associated with lower socioeconomic levels^70^, which may lead to poor housing environments, little preventive knowledge, and limited access to health care^71^. Behavioral differences because of socioeconomic status may explain the differences between education levels. Compared with males, females have slightly greater airway reactivity, smaller airways than males, and different deposition of particles^72^. Different physiological responses to ambient temperatures and air pollutants by sex might be one reason for disparities in females and males^68^. The higher illiterate rate in females and the less unfavorable socioeconomic status of females also make them more vulnerable than males^19,68^.

Limitations also exist. First, the exposure was estimated at the city level, which may lead to an estimation bias due to the difference from the personal exposure. Further investigations with individual-level exposure information are suggested to explore the possible interactive effects of extreme temperatures and air pollution on cause-specific mortality. Second, joint and independent effects had spatial variations, so the results of this study cannot be generalized to other areas.

## Conclusion

In conclusion, our study yields evidence of the interactive effects of extreme temperatures and PM_2.5_ pollution on total and cause-specific mortalities. The interactions were more robust between hot extremes and PM_2.5_ pollution. With the more frequent co-occurrence of extreme temperatures and PM_2.5_ pollution, our findings strongly support the need to further continue improving air pollution abatement, especially during hot seasons. Special measures should target females and the less educated to reduce joint effects on the vulnerable group.

## Supplementary Information

Supplementary Information.

## Data Availability

The data that support the findings of this study are available on request from the corresponding author.
