# Supplementary material for: The interactive effects of extreme temperatures and PM2.5 pollution on mortalities in Jiangsu Province, China
Source: Sci Rep. 2023 Jun 10;13:9479. doi: 10.1038/s41598-023-36635-x (PMC10257702; doi:10.1038/s41598-023-36635-x)
Supplement: Supplementary file 1 — Supplementary Information. [file 41598_2023_36635_MOESM1_ESM.docx]

**The Interactive Effects of Extreme Temperatures and PM_2.5_ Pollution on Mortalities in Jiangsu Province, China**

Lian Zhou^1^, Yuning Wang^1^, Qingqing Wang, Zhen Ding, Hui Jin, Ting Zhang, Baoli Zhu

**^1^**These authors contributed equally.

**Table of Contents**

**Fig. S1.** The distribution of temperature extremes and PM_2.5_ pollution events in 3 regions in Jiangsu, 2015 to 2019.

**Fig. S2.** The relative excess risk due to interaction (RERI) of cold/hot extremes (<5^th^/>95^th^ percentile) and PM_2.5_ pollution (> 35 μg m^-3^) pollution on mortalities stratified by population age in the cold (a) and hot (b) seasons in Jiangsu Province during 2015-2019.

**Fig. S3.** The acute relative risks (RR) and cumulative relative risks (CRR) of total and cause-specific mortalities associated with cold (a) or hot (b) extremes at lag 0-27 or lag 0-7, respectively, in three regions in Jiangsu Province in 2015-2019.

**Fig. S4.** The overall lag structure in effects of combined events of cold extremes and PM_2.5_ pollution or hot extremes and PM_2.5_ on total mortality in three regions in Jiangsu Province in 2015-2019.

**Fig. S5.** The cumulative relative risks (CRR) of total and cause-specific mortalities associated with cold (a) or hot (b) extremes at lag 0-27 or lag 0-7, respectively, in three regions in Jiangsu Province in 2015-2019.

**Fig. S6.** The cumulative relative risks (CRR) of total and cause-specific mortalities associated with PM_2.5_ pollution (>35 μg m^-3^) events in the cold (a) and hot (b) seasons at lag 0-6 at region levels in Jiangsu Province during 2015-2019.

**Table S1.** Corresponding temperature (℃) and PM2.5 concentration (μg m-3) at thresholds of extreme temperatures and PM2.5 pollution in three regions in Jiangsu province, 2015-2019.

**Table S2**. Pooled relative risk (RR) of total and cause-specific mortalities associated with temperature extremes (< 5^th^/> 95^th^ percentile of mean temperature) and PM_2.5_ pollution (> 35 μg m^-3^)) in three regions of Jiangsu Province, 2015-2019.

**Table S3.** Pooled relative risk (RR) of total and cause-specific mortalities associated with temperature extremes (< 5^th^/> 95^th^ percentile of mean temperature) and PM_2.5_ pollution (> 35 μg m^-3^) in 13 cities of Jiangsu Province, 2015-2019.

**Table S4**. The relative excess risk due to interaction (RERI) of temperature extremes and PM_2.5_ pollution in three regions of Jiangsu, 2015-2019.

**Table S5.** Differences between RERIs of extremely hot and cold temperatures and PM_2.5_ pollution in 13 cities of Jiangsu, 2015 to 2019.

**Table S6.** Sensitive analysis results of the relative excess risk due to interaction (RERI) of extreme temperatures and PM_2.5_ using '*DOW*' instead of '*Holiday*' in three regions of Jiangsu, 2015-2019.

**Table S7.** Sensitive analysis results of the relative excess risk due to interaction (RERI) of extreme temperatures and PM_2.5_ pollution using natural cubic spline smooth function to the calendar day instead of '*year*' in three regions of Jiangsu, 2015-2019.

**Table S8.** Sensitive analysis of the interactions between temperature extremes and PM_2.5_ pollution using different extreme temperature and PM_2.5_ pollution thresholds in 3 regions of Jiangsu, 2015 to 2019.


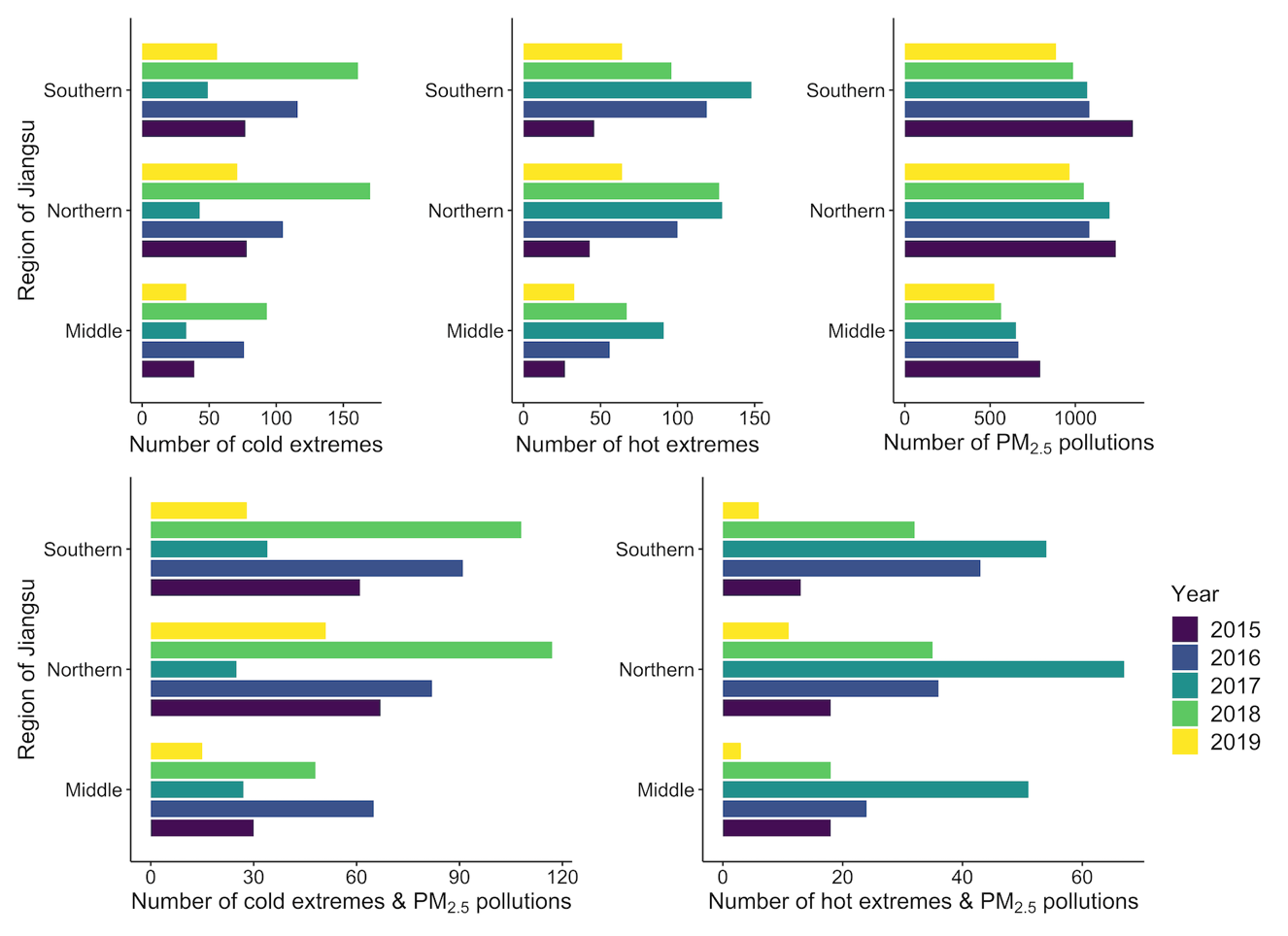


Fig. S1 The distribution of temperature extremes and PM_2.5_ pollution events in 3 regions in Jiangsu, 2015 to 2019.


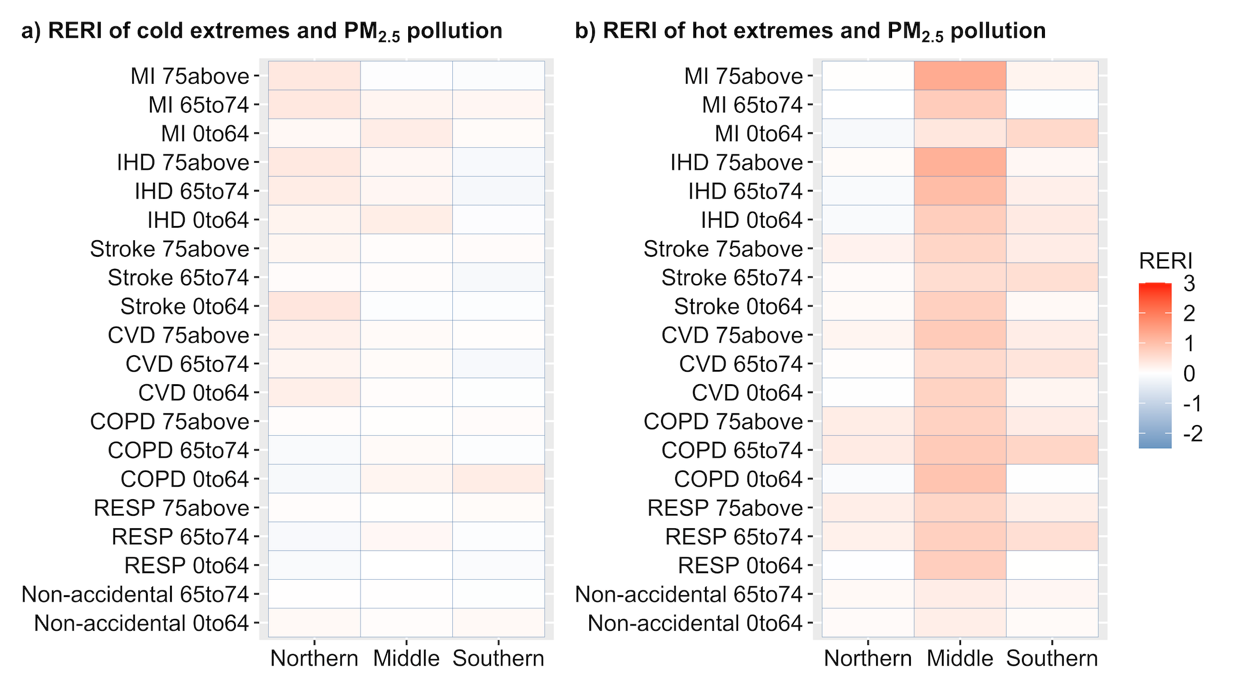


Fig. S2 The relative excess risk due to interaction (RERI) of cold/hot extremes (<5^th^/>95^th^ percentile) and PM_2.5_ pollution (> 35 μg m^-3^) pollution on mortalities stratified by population age in the cold (a) and hot (b) seasons in Jiangsu Province during 2015-2019.


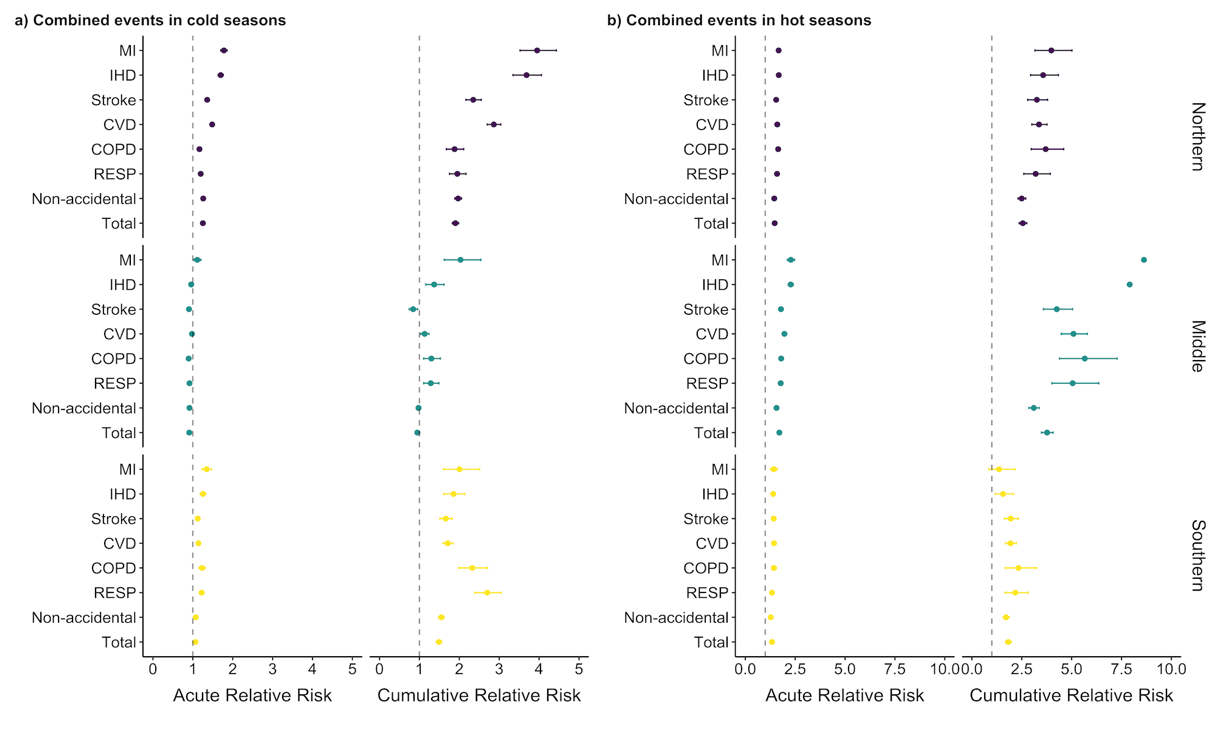


Fig. S3. The acute relative risks (RR) and cumulative relative risks (CRR) of total and cause-specific mortalities associated with cold (a) or hot (b) extremes at lag 0-27 or lag 0-7, respectively, in three regions in Jiangsu Province in 2015-2019.


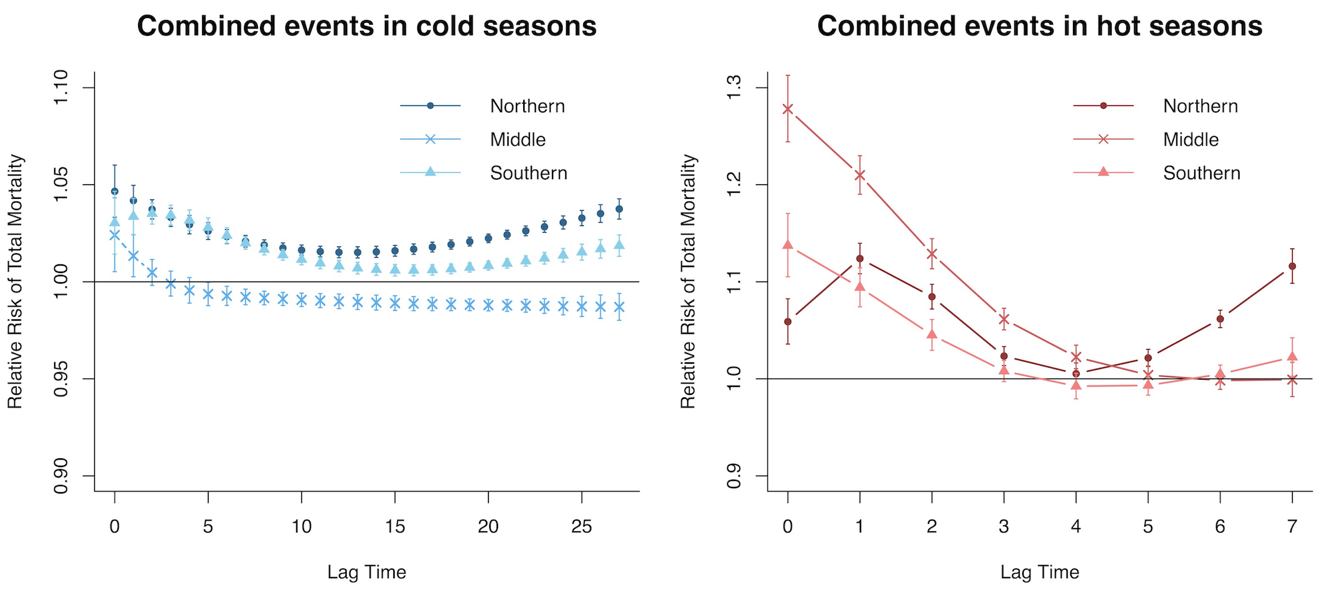


Fig. S4. The overall lag structure in effects of combined events of cold extremes and PM_2.5_ pollution or hot extremes and PM_2.5_ on total mortality in three regions in Jiangsu Province in 2015-2019.


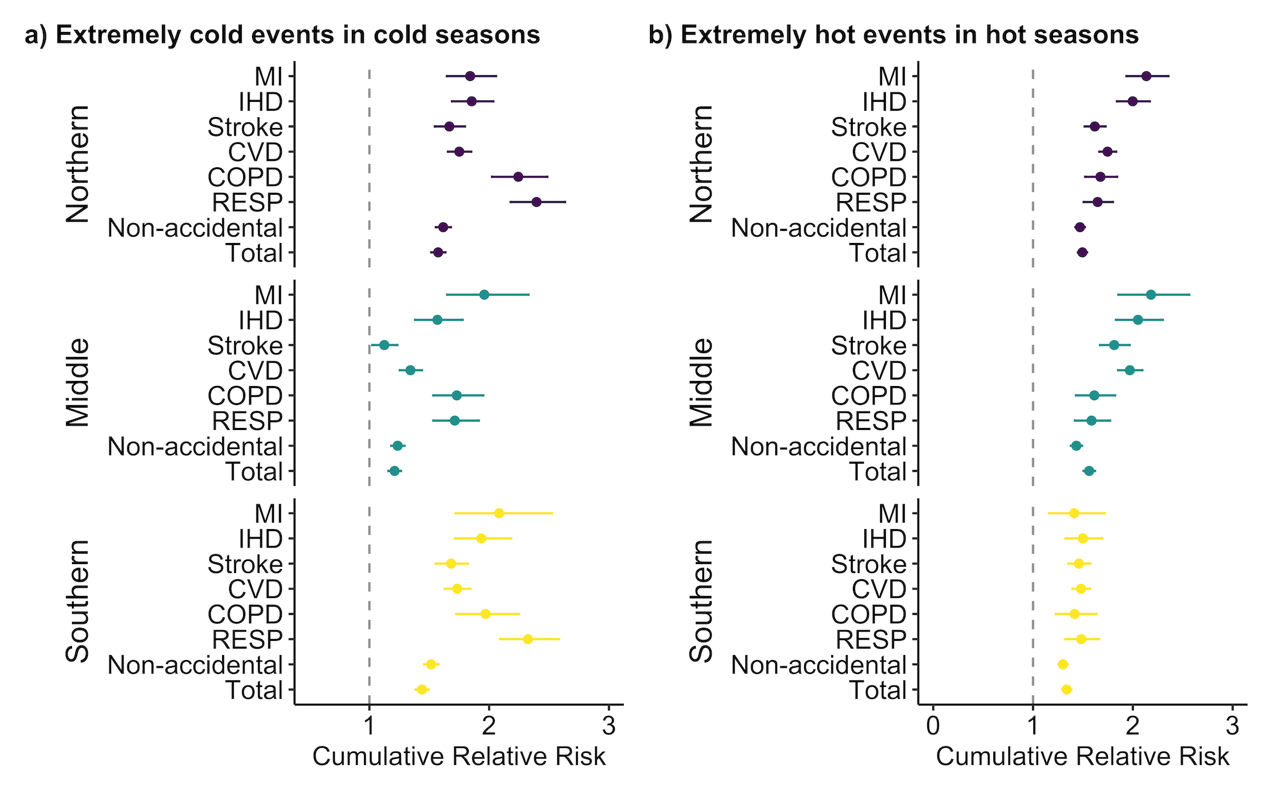


Fig. S5. The cumulative relative risks (CRR) of total and cause-specific mortalities associated with cold (a) or hot (b) extremes at lag 0-27 or lag 0-7, respectively, in three regions in Jiangsu Province in 2015-2019.


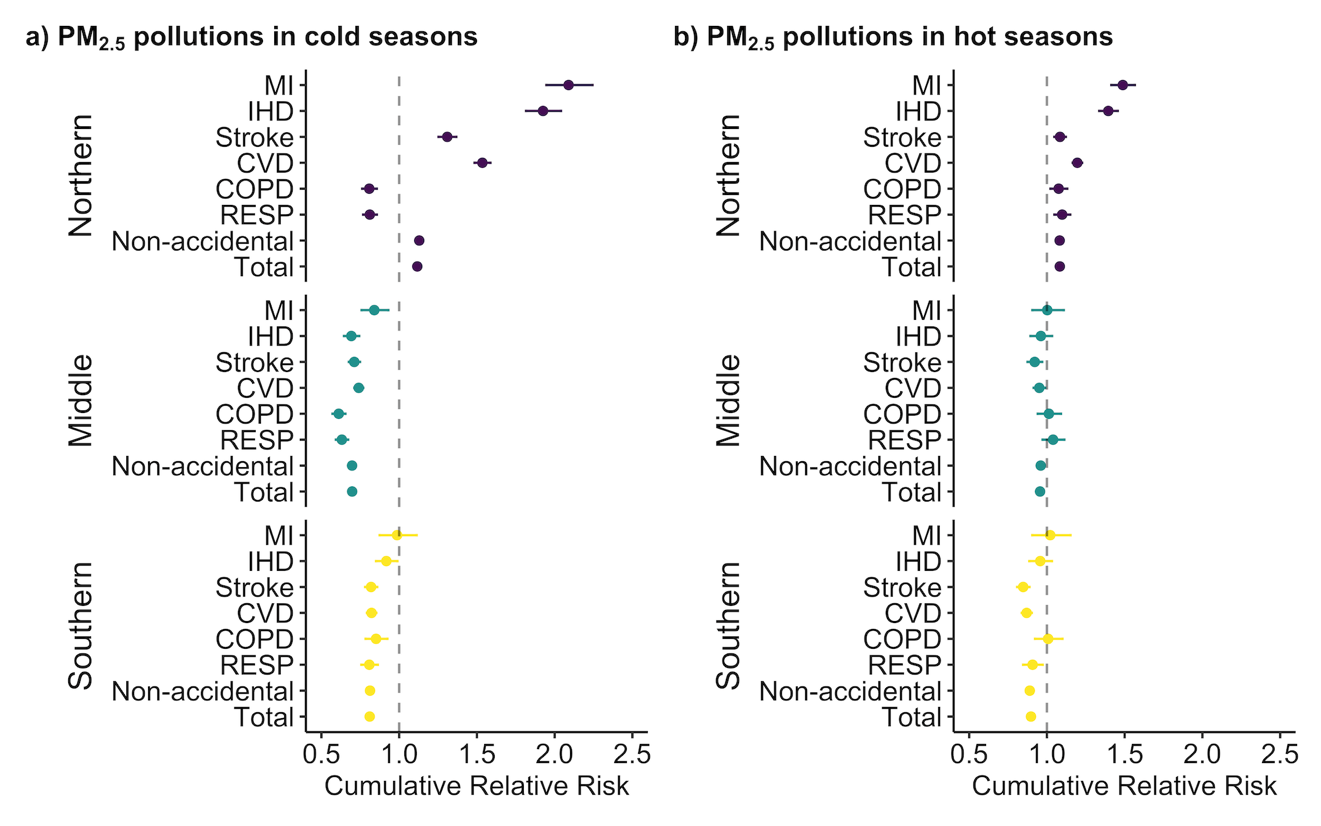


Fig. S6. The cumulative relative risks (CRR) of total and cause-specific mortalities associated with PM_2.5_ pollution (>35 μg m^-3^) events in the cold (a) and hot (b) seasons at lag 0-6 at region levels in Jiangsu Province during 2015-2019.

Table S1 Corresponding temperature (℃) and PM_2.5_ concentration (μg m^-3^) at thresholds of extreme temperatures and PM_2.5_ pollution in three regions in Jiangsu province, 2015-2019.

| Region | Daily Mean Temperature | |  | Daily Meaen PM_2.5_ concentration (μg m^-3^) | | | | |
| --- | --- | --- | --- | --- | --- | --- | --- | --- |
|  |  |  |  | Cold Seasons | |  | Hot Seasons | |
|  | 2.5^th^ percentile in the study period | 97.5^th^ percentile in the study period |  | 75^th^ percentile in the study period | 50^th^ percentile in the study period |  | 75^th^ percentile in the study period | 50^th^ percentile in the study period |
| Northern Jiangsu | -2.4 | 31.5 |  | 92.9 | 63.1 |  | 41.6 | 30.3 |
| Middle Jiangsu | -1.1 | 32.2 |  | 83.2 | 57 |  | 43.2 | 30.7 |
| Southern Jiangsu | -0.3 | 33.5 |  | 81.7 | 56.9 |  | 41 | 30.3 |

Table S2 Pooled relative risk (RR) of total and cause-specific mortalities associated with temperature extremes (< 5^th^/> 95^th^ percentile of mean temperature) and PM_2.5_ pollution (> 35 μg m^-3^)) in three regions of Jiangsu Province, 2015-2019

| Region | Outcome | Cold Seasons | |  | Hot Seasons | |
| --- | --- | --- | --- | --- | --- | --- |
|  |  | RR of cold extremes (95% CI) | RR of PM_2.5_ pollution (95% CI) |  | RR of hot extremes (95% CI) | RR of PM_2.5_ pollution (95% CI) |
| Southern | Total | 1.084 (1.070, 1.099) | 0.971 (0.963, 0.978) |  | 1.251 (1.232, 1.269) | 0.973 (0.966, 0.981) |
|  | Non-accidental | 1.094 (1.079, 1.110) | 0.972 (0.964, 0.980) |  | 1.210 (1.191, 1.229) | 0.969 (0.961, 0.976) |
|  | Stroke | 1.140 (1.108, 1.173) | 0.980 (0.964, 0.997) |  | 1.303 (1.261, 1.345) | 0.970 (0.954, 0.986) |
|  | CVD | 1.161 (1.136, 1.187) | 0.982 (0.969, 0.995) |  | 1.331 (1.298, 1.365) | 0.970 (0.957, 0.982) |
|  | RESP | 1.206 (1.161, 1.253) | 1.010 (0.987, 1.034) |  | 1.249 (1.189, 1.311) | 0.963 (0.940, 0.987) |
|  | IHD | 1.263 (1.210, 1.318) | 1.019 (0.992, 1.046) |  | 1.332 (1.264, 1.402) | 0.986 (0.960, 1.013) |
|  | MI | 1.292 (1.207, 1.381) | 1.048 (1.005, 1.094) |  | 1.315 (1.209, 1.428) | 1.002 (0.961, 1.046) |
|  | COPD | 1.206 (1.149, 1.265) | 1.015 (0.986, 1.045) |  | 1.270 (1.195, 1.350) | 1.004 (0.974, 1.036) |
|  | Total 0to64 | 1.086 (1.049, 1.124) | 0.980 (0.961, 1.000) |  | 1.184 (1.142, 1.227) | 0.971 (0.954, 0.988) |
|  | Total 65to74 | 1.025 (0.991, 1.059) | 0.958 (0.941, 0.976) |  | 1.142 (1.103, 1.182) | 0.973 (0.957, 0.990) |
|  | Total 75above | 1.100 (1.082, 1.118) | 0.972 (0.963, 0.981) |  | 1.304 (1.280, 1.328) | 0.974 (0.965, 0.983) |
|  | Nonaccidental 0to64 | 1.104 (1.064, 1.145) | 0.976 (0.956, 0.997) |  | 1.139 (1.094, 1.185) | 0.968 (0.950, 0.987) |
|  | Nonaccidental 65to74 | 1.043 (1.008, 1.079) | 0.960 (0.942, 0.979) |  | 1.100 (1.060, 1.141) | 0.970 (0.953, 0.987) |
|  | Nonaccidental 75above | 1.106 (1.087, 1.125) | 0.974 (0.964, 0.983) |  | 1.262 (1.238, 1.286) | 0.968 (0.959, 0.978) |
|  | RESP 0to64 | 1.099 (0.921, 1.299) | 1.163 (1.049, 1.293) |  | 1.122 (0.906, 1.377) | 1.097 (0.992, 1.212) |
|  | RESP 65to74 | 1.278 (1.136, 1.431) | 1.037 (0.965, 1.115) |  | 1.366 (1.179, 1.576) | 1.020 (0.948, 1.098) |
|  | RESP 75above | 1.205 (1.155, 1.256) | 0.998 (0.973, 1.024) |  | 1.244 (1.178, 1.312) | 0.947 (0.922, 0.973) |
|  | COPD 0to64 | 1.052 (0.814, 1.335) | 1.157 (1.001, 1.343) |  | 1.335 (1.010, 1.740) | 1.226 (1.067, 1.408) |
|  | COPD 65to74 | 1.274 (1.099, 1.469) | 1.049 (0.959, 1.149) |  | 1.500 (1.258, 1.778) | 1.085 (0.990, 1.188) |
|  | COPD 75above | 1.206 (1.145, 1.270) | 1.005 (0.974, 1.037) |  | 1.239 (1.158, 1.324) | 0.982 (0.949, 1.016) |
|  | CVD 0to64 | 1.140 (1.059, 1.224) | 0.999 (0.958, 1.043) |  | 1.267 (1.168, 1.372) | 0.999 (0.960, 1.040) |
|  | CVD 65to74 | 1.084 (1.019, 1.152) | 1.008 (0.972, 1.044) |  | 1.282 (1.199, 1.370) | 0.984 (0.951, 1.018) |
|  | CVD 75above | 1.177 (1.148, 1.207) | 0.975 (0.961, 0.990) |  | 1.350 (1.312, 1.390) | 0.963 (0.949, 0.978) |
|  | Stroke 0to64 | 1.103 (0.997, 1.218) | 1.014 (0.957, 1.075) |  | 1.184 (1.058, 1.322) | 1.011 (0.958, 1.067) |
|  | Stroke 65to74 | 1.077 (0.996, 1.163) | 1.005 (0.961, 1.051) |  | 1.297 (1.193, 1.408) | 0.972 (0.931, 1.014) |
|  | Stroke 75above | 1.157 (1.120, 1.194) | 0.972 (0.954, 0.991) |  | 1.319 (1.270, 1.368) | 0.964 (0.946, 0.983) |
|  | IHD 0to64 | 1.190 (1.034, 1.362) | 1.001 (0.922, 1.089) |  | 1.374 (1.174, 1.599) | 1.030 (0.952, 1.114) |
|  | IHD 65to74 | 1.160 (1.023, 1.309) | 1.096 (1.017, 1.183) |  | 1.221 (1.054, 1.408) | 1.005 (0.936, 1.079) |
|  | IHD 75above | 1.291 (1.229, 1.354) | 1.009 (0.979, 1.040) |  | 1.346 (1.268, 1.429) | 0.976 (0.946, 1.007) |
|  | MI 0to64 | 1.229 (1.030, 1.454) | 0.993 (0.894, 1.105) |  | 1.278 (1.038, 1.559) | 1.026 (0.927, 1.135) |
|  | MI 65to74 | 1.122 (0.942, 1.327) | 1.130 (1.019, 1.255) |  | 1.025 (0.819, 1.267) | 1.050 (0.950, 1.160) |
|  | MI 75above | 1.352 (1.246, 1.466) | 1.042 (0.989, 1.098) |  | 1.405 (1.269, 1.553) | 0.983 (0.933, 1.037) |
|  | Nonaccidental male | 1.086 (1.065, 1.107) | 0.969 (0.959, 0.980) |  | 1.149 (1.125, 1.174) | 0.968 (0.958, 0.978) |
|  | Nonaccidental female | 1.104 (1.081, 1.127) | 0.975 (0.963, 0.987) |  | 1.286 (1.257, 1.316) | 0.970 (0.958, 0.981) |
|  | RESP male | 1.189 (1.130, 1.250) | 0.997 (0.967, 1.027) |  | 1.166 (1.092, 1.244) | 0.958 (0.928, 0.989) |
|  | RESP female | 1.231 (1.160, 1.305) | 1.030 (0.993, 1.068) |  | 1.370 (1.272, 1.475) | 0.971 (0.934, 1.008) |
|  | COPD male | 1.201 (1.129, 1.276) | 1.003 (0.967, 1.041) |  | 1.218 (1.125, 1.317) | 0.996 (0.957, 1.036) |
|  | COPD female | 1.216 (1.125, 1.312) | 1.035 (0.987, 1.085) |  | 1.358 (1.232, 1.494) | 1.018 (0.968, 1.070) |
|  | CVD male | 1.156 (1.120, 1.192) | 0.979 (0.961, 0.997) |  | 1.273 (1.227, 1.319) | 0.969 (0.952, 0.987) |
|  | CVD female | 1.166 (1.130, 1.203) | 0.985 (0.967, 1.003) |  | 1.391 (1.343, 1.441) | 0.970 (0.953, 0.988) |
|  | Stroke male | 1.144 (1.098, 1.190) | 0.979 (0.956, 1.002) |  | 1.255 (1.198, 1.314) | 0.980 (0.957, 1.003) |
|  | Stroke female | 1.137 (1.092, 1.184) | 0.982 (0.959, 1.006) |  | 1.351 (1.291, 1.413) | 0.960 (0.937, 0.982) |
|  | IHD male | 1.240 (1.167, 1.317) | 0.997 (0.961, 1.035) |  | 1.259 (1.168, 1.355) | 0.968 (0.932, 1.005) |
|  | IHD female | 1.287 (1.211, 1.366) | 1.042 (1.003, 1.083) |  | 1.409 (1.309, 1.514) | 1.005 (0.968, 1.044) |
|  | MI male | 1.264 (1.153, 1.382) | 1.007 (0.952, 1.065) |  | 1.204 (1.071, 1.348) | 1.004 (0.949, 1.063) |
|  | MI female | 1.329 (1.199, 1.469) | 1.107 (1.037, 1.183) |  | 1.459 (1.291, 1.644) | 1.000 (0.938, 1.066) |
|  | Nonaccidental Edu1 | 1.100 (0.945, 1.271) | 1.076 (0.988, 1.172) |  | 0.972 (0.805, 1.162) | 1.002 (0.920, 1.090) |
|  | Nonaccidental Edu2 | 1.140 (1.048, 1.237) | 0.984 (0.938, 1.033) |  | 1.044 (0.945, 1.152) | 1.035 (0.988, 1.085) |
|  | Nonaccidental Edu3 | 1.014 (0.996, 1.032) | 0.965 (0.955, 0.974) |  | 1.276 (1.251, 1.301) | 0.993 (0.984, 1.003) |
|  | RESP Edu1 | 0.991 (0.601, 1.536) | 0.941 (0.736, 1.216) |  | 0.865 (0.420, 1.593) | 0.970 (0.712, 1.310) |
|  | RESP Edu2 | 1.370 (1.066, 1.732) | 1.052 (0.904, 1.229) |  | 0.999 (0.692, 1.400) | 1.043 (0.884, 1.227) |
|  | RESP Edu3 | 1.047 (1.001, 1.095) | 1.015 (0.989, 1.041) |  | 1.280 (1.213, 1.350) | 1.022 (0.994, 1.050) |
|  | COPD Edu1 | 0.695 (0.271, 1.448) | 1.205 (0.812, 1.839) |  | 0.599 (0.144, 1.664) | 0.832 (0.515, 1.308) |
|  | COPD Edu2 | 0.987 (0.938, 1.037) | 1.000 (0.973, 1.028) |  | 1.335 (1.260, 1.414) | 1.031 (1.001, 1.062) |
|  | COPD Edu3 | 1.507 (1.104, 2.008) | 0.969 (0.800, 1.180) |  | 1.036 (0.643, 1.591) | 1.002 (0.809, 1.237) |
|  | CVD Edu1 | 1.292 (1.002, 1.638) | 1.026 (0.885, 1.194) |  | 1.099 (0.775, 1.516) | 1.011 (0.861, 1.184) |
|  | CVD Edu2 | 1.168 (1.012, 1.341) | 0.942 (0.868, 1.024) |  | 1.055 (0.874, 1.262) | 1.030 (0.945, 1.122) |
|  | CVD Edu3 | 1.036 (1.009, 1.065) | 0.952 (0.938, 0.967) |  | 1.421 (1.379, 1.464) | 0.974 (0.959, 0.989) |
|  | Stroke Edu1 | 0.904 (0.570, 1.357) | 1.189 (0.946, 1.508) |  | 1.246 (0.751, 1.960) | 0.998 (0.785, 1.261) |
|  | Stroke Edu2 | 1.245 (1.021, 1.503) | 0.933 (0.832, 1.049) |  | 1.083 (0.832, 1.387) | 1.088 (0.966, 1.224) |
|  | Stroke Edu3 | 1.016 (0.981, 1.053) | 0.951 (0.933, 0.970) |  | 1.415 (1.361, 1.471) | 0.984 (0.964, 1.005) |
|  | IHD Edu1 | 1.281 (0.820, 1.905) | 0.942 (0.736, 1.218) |  | 1.104 (0.586, 1.911) | 1.139 (0.856, 1.505) |
|  | IHD Edu2 | 1.049 (0.798, 1.352) | 0.947 (0.818, 1.100) |  | 1.187 (0.862, 1.599) | 1.029 (0.883, 1.198) |
|  | IHD Edu3 | 0.988 (0.936, 1.041) | 0.947 (0.919, 0.975) |  | 1.442 (1.360, 1.527) | 0.964 (0.935, 0.994) |
|  | MI Edu1 | 1.154 (0.605, 1.993) | 0.963 (0.691, 1.367) |  | 0.837 (0.320, 1.810) | 1.196 (0.825, 1.717) |
|  | MI Edu2 | 1.086 (0.770, 1.485) | 0.875 (0.728, 1.057) |  | 1.478 (1.013, 2.099) | 1.020 (0.839, 1.237) |
|  | MI Edu3 | 0.938 (0.874, 1.005) | 0.922 (0.888, 0.957) |  | 1.413 (1.309, 1.523) | 0.962 (0.925, 1.000) |
| Northern | Total | 1.113 (1.100, 1.127) | 1.104 (1.096, 1.112) |  | 1.350 (1.333, 1.366) | 1.046 (1.039, 1.053) |
|  | Non-accidental | 1.117 (1.103, 1.131) | 1.109 (1.100, 1.117) |  | 1.330 (1.313, 1.348) | 1.047 (1.040, 1.054) |
|  | Stroke | 1.149 (1.122, 1.177) | 1.161 (1.143, 1.178) |  | 1.409 (1.373, 1.447) | 1.060 (1.045, 1.075) |
|  | CVD | 1.169 (1.149, 1.190) | 1.243 (1.229, 1.257) |  | 1.441 (1.413, 1.469) | 1.093 (1.081, 1.105) |
|  | RESP | 1.164 (1.128, 1.200) | 1.021 (1.002, 1.040) |  | 1.424 (1.374, 1.476) | 1.051 (1.031, 1.072) |
|  | IHD | 1.218 (1.182, 1.254) | 1.363 (1.336, 1.391) |  | 1.491 (1.443, 1.541) | 1.142 (1.122, 1.163) |
|  | MI | 1.224 (1.182, 1.268) | 1.427 (1.393, 1.463) |  | 1.475 (1.418, 1.534) | 1.160 (1.136, 1.186) |
|  | COPD | 1.153 (1.115, 1.192) | 1.005 (0.986, 1.026) |  | 1.467 (1.412, 1.523) | 1.044 (1.022, 1.066) |
|  | Total 0to64 | 1.093 (1.063, 1.125) | 1.141 (1.121, 1.160) |  | 1.271 (1.236, 1.306) | 1.048 (1.033, 1.063) |
|  | Total 65to74 | 1.085 (1.053, 1.118) | 1.124 (1.104, 1.144) |  | 1.259 (1.222, 1.298) | 1.061 (1.045, 1.078) |
|  | Total 75above | 1.182 (1.164, 1.200) | 1.178 (1.166, 1.189) |  | 1.464 (1.440, 1.489) | 1.080 (1.070, 1.090) |
|  | Nonaccidental 0to64 | 1.090 (1.057, 1.125) | 1.138 (1.117, 1.160) |  | 1.234 (1.196, 1.274) | 1.052 (1.035, 1.069) |
|  | Nonaccidental 65to74 | 1.085 (1.052, 1.118) | 1.126 (1.105, 1.147) |  | 1.243 (1.205, 1.283) | 1.062 (1.045, 1.080) |
|  | Nonaccidental 75above | 1.186 (1.167, 1.205) | 1.183 (1.171, 1.195) |  | 1.441 (1.416, 1.466) | 1.079 (1.068, 1.089) |
|  | RESP 0to64 | 1.179 (1.027, 1.346) | 1.073 (0.989, 1.167) |  | 1.198 (1.019, 1.397) | 1.042 (0.961, 1.128) |
|  | RESP 65to74 | 1.207 (1.100, 1.322) | 1.066 (1.007, 1.129) |  | 1.346 (1.209, 1.493) | 1.052 (0.994, 1.112) |
|  | RESP 75above | 1.191 (1.150, 1.233) | 1.066 (1.043, 1.089) |  | 1.489 (1.430, 1.551) | 1.071 (1.047, 1.095) |
|  | COPD 0to64 | 1.142 (0.962, 1.346) | 1.021 (0.925, 1.129) |  | 1.317 (1.094, 1.571) | 1.012 (0.920, 1.112) |
|  | COPD 65to74 | 1.170 (1.058, 1.290) | 1.026 (0.967, 1.089) |  | 1.391 (1.242, 1.553) | 1.048 (0.988, 1.113) |
|  | COPD 75above | 1.181 (1.138, 1.226) | 1.041 (1.018, 1.065) |  | 1.508 (1.445, 1.574) | 1.063 (1.038, 1.088) |
|  | CVD 0to64 | 1.139 (1.080, 1.200) | 1.285 (1.242, 1.329) |  | 1.335 (1.263, 1.409) | 1.117 (1.085, 1.150) |
|  | CVD 65to74 | 1.152 (1.099, 1.207) | 1.309 (1.269, 1.349) |  | 1.318 (1.253, 1.386) | 1.141 (1.111, 1.172) |
|  | CVD 75above | 1.228 (1.202, 1.255) | 1.320 (1.302, 1.339) |  | 1.533 (1.497, 1.570) | 1.114 (1.100, 1.129) |
|  | Stroke 0to64 | 1.151 (1.068, 1.237) | 1.235 (1.179, 1.294) |  | 1.311 (1.214, 1.414) | 1.127 (1.083, 1.173) |
|  | Stroke 65to74 | 1.091 (1.024, 1.160) | 1.234 (1.187, 1.283) |  | 1.339 (1.254, 1.428) | 1.084 (1.047, 1.122) |
|  | Stroke 75above | 1.212 (1.177, 1.247) | 1.211 (1.189, 1.234) |  | 1.490 (1.442, 1.538) | 1.074 (1.055, 1.093) |
|  | IHD 0to64 | 1.133 (1.038, 1.236) | 1.354 (1.279, 1.435) |  | 1.365 (1.246, 1.493) | 1.106 (1.053, 1.161) |
|  | IHD 65to74 | 1.294 (1.195, 1.400) | 1.464 (1.384, 1.549) |  | 1.283 (1.171, 1.401) | 1.268 (1.210, 1.329) |
|  | IHD 75above | 1.272 (1.228, 1.318) | 1.479 (1.442, 1.516) |  | 1.609 (1.546, 1.674) | 1.165 (1.139, 1.192) |
|  | MI 0to64 | 1.104 (1.000, 1.215) | 1.397 (1.311, 1.491) |  | 1.364 (1.232, 1.505) | 1.102 (1.044, 1.162) |
|  | MI 65to74 | 1.306 (1.191, 1.429) | 1.506 (1.411, 1.610) |  | 1.289 (1.161, 1.427) | 1.278 (1.211, 1.348) |
|  | MI 75above | 1.279 (1.225, 1.335) | 1.556 (1.508, 1.605) |  | 1.592 (1.516, 1.671) | 1.195 (1.162, 1.228) |
|  | Nonaccidental male | 1.116 (1.098, 1.135) | 1.110 (1.098, 1.121) |  | 1.254 (1.231, 1.277) | 1.044 (1.034, 1.054) |
|  | Nonaccidental female | 1.118 (1.098, 1.139) | 1.107 (1.095, 1.120) |  | 1.425 (1.398, 1.453) | 1.051 (1.040, 1.062) |
|  | RESP male | 1.167 (1.119, 1.218) | 1.027 (1.001, 1.054) |  | 1.276 (1.212, 1.343) | 1.053 (1.025, 1.081) |
|  | RESP female | 1.159 (1.108, 1.212) | 1.014 (0.987, 1.041) |  | 1.592 (1.514, 1.672) | 1.050 (1.021, 1.079) |
|  | COPD male | 1.162 (1.110, 1.216) | 1.000 (0.973, 1.028) |  | 1.313 (1.242, 1.387) | 1.042 (1.012, 1.072) |
|  | COPD female | 1.142 (1.088, 1.199) | 1.011 (0.982, 1.041) |  | 1.639 (1.554, 1.727) | 1.047 (1.015, 1.079) |
|  | CVD male | 1.175 (1.146, 1.205) | 1.247 (1.227, 1.268) |  | 1.375 (1.337, 1.413) | 1.091 (1.075, 1.108) |
|  | CVD female | 1.163 (1.134, 1.193) | 1.238 (1.219, 1.259) |  | 1.509 (1.469, 1.551) | 1.095 (1.078, 1.111) |
|  | Stroke male | 1.157 (1.119, 1.196) | 1.174 (1.149, 1.199) |  | 1.345 (1.296, 1.396) | 1.053 (1.032, 1.074) |
|  | Stroke female | 1.140 (1.101, 1.180) | 1.147 (1.123, 1.172) |  | 1.479 (1.425, 1.534) | 1.067 (1.045, 1.088) |
|  | IHD male | 1.233 (1.182, 1.286) | 1.366 (1.328, 1.406) |  | 1.431 (1.365, 1.500) | 1.152 (1.123, 1.182) |
|  | IHD female | 1.203 (1.154, 1.253) | 1.360 (1.323, 1.399) |  | 1.552 (1.483, 1.624) | 1.133 (1.104, 1.162) |
|  | MI male | 1.270 (1.209, 1.333) | 1.417 (1.370, 1.467) |  | 1.444 (1.367, 1.525) | 1.150 (1.115, 1.185) |
|  | MI female | 1.178 (1.120, 1.239) | 1.438 (1.389, 1.489) |  | 1.509 (1.426, 1.595) | 1.172 (1.136, 1.210) |
|  | Nonaccidental Edu1 | 0.895 (0.796, 1.003) | 0.934 (0.878, 0.995) |  | 0.904 (0.789, 1.029) | 0.788 (0.745, 0.833) |
|  | Nonaccidental Edu2 | 0.939 (0.873, 1.008) | 0.979 (0.941, 1.019) |  | 1.026 (0.943, 1.113) | 0.876 (0.845, 0.909) |
|  | Nonaccidental Edu3 | 1.043 (1.026, 1.060) | 1.009 (0.999, 1.018) |  | 1.044 (1.022, 1.066) | 0.990 (0.981, 0.999) |
|  | RESP Edu1 | 0.898 (0.601, 1.289) | 1.056 (0.858, 1.313) |  | 0.718 (0.424, 1.130) | 0.787 (0.651, 0.948) |
|  | RESP Edu2 | 0.898 (0.698, 1.135) | 0.951 (0.835, 1.086) |  | 1.223 (0.916, 1.598) | 0.943 (0.826, 1.075) |
|  | RESP Edu3 | 1.060 (1.018, 1.102) | 1.015 (0.991, 1.040) |  | 1.111 (1.049, 1.175) | 1.035 (1.010, 1.062) |
|  | COPD Edu1 | 0.347 (0.123, 0.762) | 1.056 (0.765, 1.493) |  | 1.131 (0.533, 2.100) | 0.821 (0.598, 1.120) |
|  | COPD Edu2 | 1.060 (1.014, 1.107) | 1.020 (0.993, 1.047) |  | 1.119 (1.049, 1.192) | 1.059 (1.029, 1.089) |
|  | COPD Edu3 | 0.901 (0.636, 1.237) | 0.940 (0.787, 1.129) |  | 1.334 (0.905, 1.894) | 1.108 (0.926, 1.323) |
|  | CVD Edu1 | 0.886 (0.725, 1.072) | 0.913 (0.824, 1.014) |  | 1.029 (0.818, 1.275) | 0.792 (0.717, 0.875) |
|  | CVD Edu2 | 1.019 (0.909, 1.139) | 0.987 (0.924, 1.054) |  | 1.106 (0.959, 1.267) | 0.882 (0.828, 0.939) |
|  | CVD Edu3 | 1.109 (1.084, 1.136) | 1.072 (1.056, 1.087) |  | 1.121 (1.086, 1.158) | 1.002 (0.988, 1.016) |
|  | Stroke Edu1 | 0.946 (0.697, 1.252) | 0.884 (0.757, 1.038) |  | 1.118 (0.805, 1.511) | 0.787 (0.679, 0.911) |
|  | Stroke Edu2 | 1.030 (0.875, 1.203) | 0.962 (0.878, 1.056) |  | 1.030 (0.838, 1.251) | 0.872 (0.798, 0.952) |
|  | Stroke Edu3 | 1.084 (1.050, 1.118) | 1.022 (1.003, 1.042) |  | 1.093 (1.047, 1.140) | 0.983 (0.965, 1.002) |
|  | IHD Edu1 | 0.695 (0.485, 0.963) | 0.911 (0.775, 1.078) |  | 0.945 (0.630, 1.357) | 0.742 (0.627, 0.875) |
|  | IHD Edu2 | 1.069 (0.885, 1.278) | 0.989 (0.887, 1.104) |  | 1.232 (0.972, 1.538) | 0.888 (0.797, 0.989) |
|  | IHD Edu3 | 1.177 (1.132, 1.225) | 1.158 (1.129, 1.188) |  | 1.204 (1.139, 1.272) | 1.045 (1.019, 1.072) |
|  | MI Edu1 | 0.719 (0.436, 1.111) | 0.880 (0.705, 1.109) |  | 1.128 (0.663, 1.790) | 0.797 (0.628, 1.007) |
|  | MI Edu2 | 1.133 (0.891, 1.419) | 1.035 (0.898, 1.197) |  | 1.458 (1.090, 1.907) | 0.907 (0.787, 1.043) |
|  | MI Edu3 | 1.290 (1.226, 1.356) | 1.290 (1.246, 1.335) |  | 1.270 (1.180, 1.364) | 1.124 (1.088, 1.162) |
| Middle | Total | 0.961 (0.946, 0.977) | 0.927 (0.920, 0.935) |  | 1.470 (1.447, 1.493) | 0.994 (0.986, 1.003) |
|  | Non-accidental | 0.967 (0.951, 0.983) | 0.928 (0.921, 0.936) |  | 1.377 (1.355, 1.401) | 0.991 (0.982, 0.999) |
|  | Stroke | 0.968 (0.936, 1.002) | 0.925 (0.909, 0.941) |  | 1.582 (1.529, 1.637) | 0.971 (0.953, 0.989) |
|  | CVD | 1.018 (0.992, 1.044) | 0.941 (0.929, 0.954) |  | 1.670 (1.628, 1.712) | 1.000 (0.986, 1.014) |
|  | RESP | 0.992 (0.951, 1.033) | 0.917 (0.897, 0.937) |  | 1.509 (1.441, 1.580) | 1.005 (0.981, 1.030) |
|  | IHD | 1.008 (0.962, 1.056) | 0.910 (0.888, 0.933) |  | 1.801 (1.719, 1.886) | 1.025 (0.998, 1.052) |
|  | MI | 1.142 (1.071, 1.216) | 0.964 (0.931, 0.999) |  | 1.766 (1.654, 1.884) | 1.073 (1.034, 1.114) |
|  | COPD | 0.981 (0.937, 1.026) | 0.904 (0.883, 0.926) |  | 1.513 (1.438, 1.591) | 1.003 (0.976, 1.030) |
|  | Total 0to64 | 0.921 (0.882, 0.961) | 0.925 (0.905, 0.945) |  | 1.208 (1.160, 1.257) | 0.977 (0.958, 0.997) |
|  | Total 65to74 | 0.943 (0.908, 0.980) | 0.957 (0.939, 0.976) |  | 1.276 (1.230, 1.324) | 1.000 (0.981, 1.018) |
|  | Total 75above | 0.975 (0.956, 0.994) | 0.920 (0.911, 0.929) |  | 1.609 (1.579, 1.640) | 0.997 (0.987, 1.008) |
|  | Nonaccidental 0to64 | 0.908 (0.868, 0.951) | 0.911 (0.890, 0.932) |  | 1.144 (1.094, 1.196) | 0.968 (0.947, 0.989) |
|  | Nonaccidental 65to74 | 0.952 (0.915, 0.990) | 0.957 (0.938, 0.977) |  | 1.197 (1.151, 1.245) | 1.000 (0.981, 1.020) |
|  | Nonaccidental 75above | 0.983 (0.964, 1.003) | 0.925 (0.915, 0.934) |  | 1.497 (1.467, 1.527) | 0.993 (0.983, 1.004) |
|  | RESP 0to64 | 0.698 (0.582, 0.830) | 0.820 (0.756, 0.889) |  | 1.658 (1.412, 1.937) | 0.916 (0.843, 0.995) |
|  | RESP 65to74 | 0.901 (0.798, 1.013) | 0.886 (0.835, 0.942) |  | 1.414 (1.244, 1.601) | 0.968 (0.906, 1.033) |
|  | RESP 75above | 1.038 (0.991, 1.086) | 0.932 (0.909, 0.955) |  | 1.516 (1.438, 1.597) | 1.023 (0.995, 1.052) |
|  | COPD 0to64 | 0.596 (0.476, 0.736) | 0.764 (0.698, 0.838) |  | 1.628 (1.349, 1.951) | 0.849 (0.772, 0.935) |
|  | COPD 65to74 | 0.908 (0.795, 1.031) | 0.885 (0.829, 0.946) |  | 1.380 (1.198, 1.582) | 0.955 (0.889, 1.026) |
|  | COPD 75above | 1.030 (0.980, 1.082) | 0.920 (0.896, 0.945) |  | 1.533 (1.448, 1.622) | 1.029 (0.998, 1.061) |
|  | CVD 0to64 | 0.849 (0.778, 0.925) | 0.839 (0.804, 0.875) |  | 1.382 (1.274, 1.497) | 0.986 (0.946, 1.028) |
|  | CVD 65to74 | 0.916 (0.853, 0.982) | 0.916 (0.883, 0.949) |  | 1.460 (1.365, 1.559) | 0.989 (0.955, 1.025) |
|  | CVD 75above | 1.061 (1.031, 1.091) | 0.960 (0.945, 0.975) |  | 1.765 (1.714, 1.816) | 1.005 (0.988, 1.021) |
|  | Stroke 0to64 | 0.856 (0.761, 0.958) | 0.821 (0.777, 0.869) |  | 1.385 (1.244, 1.539) | 0.962 (0.910, 1.017) |
|  | Stroke 65to74 | 0.894 (0.815, 0.977) | 0.944 (0.902, 0.988) |  | 1.489 (1.368, 1.619) | 0.963 (0.921, 1.008) |
|  | Stroke 75above | 1.000 (0.962, 1.039) | 0.935 (0.916, 0.955) |  | 1.638 (1.574, 1.704) | 0.974 (0.953, 0.996) |
|  | IHD 0to64 | 0.739 (0.624, 0.869) | 0.830 (0.768, 0.897) |  | 1.511 (1.302, 1.746) | 1.001 (0.926, 1.082) |
|  | IHD 65to74 | 0.944 (0.823, 1.076) | 0.833 (0.778, 0.893) |  | 1.471 (1.291, 1.669) | 1.040 (0.971, 1.114) |
|  | IHD 75above | 1.058 (1.004, 1.115) | 0.935 (0.909, 0.961) |  | 1.919 (1.820, 2.022) | 1.026 (0.995, 1.058) |
|  | MI 0to64 | 0.866 (0.708, 1.049) | 0.884 (0.802, 0.975) |  | 1.497 (1.242, 1.792) | 1.073 (0.974, 1.183) |
|  | MI 65to74 | 1.109 (0.936, 1.304) | 0.925 (0.845, 1.013) |  | 1.374 (1.155, 1.625) | 1.045 (0.955, 1.143) |
|  | MI 75above | 1.201 (1.115, 1.291) | 0.988 (0.948, 1.030) |  | 1.925 (1.783, 2.077) | 1.080 (1.033, 1.129) |
|  | Nonaccidental male | 0.963 (0.942, 0.985) | 0.929 (0.918, 0.940) |  | 1.261 (1.232, 1.290) | 0.982 (0.971, 0.994) |
|  | Nonaccidental female | 0.972 (0.948, 0.996) | 0.928 (0.916, 0.940) |  | 1.520 (1.484, 1.557) | 1.001 (0.988, 1.014) |
|  | RESP male | 1.018 (0.964, 1.074) | 0.914 (0.888, 0.940) |  | 1.384 (1.300, 1.474) | 1.004 (0.972, 1.037) |
|  | RESP female | 0.957 (0.897, 1.020) | 0.921 (0.891, 0.953) |  | 1.684 (1.572, 1.803) | 1.007 (0.970, 1.046) |
|  | COPD male | 1.004 (0.946, 1.065) | 0.900 (0.872, 0.928) |  | 1.346 (1.256, 1.442) | 1.001 (0.966, 1.037) |
|  | COPD female | 0.950 (0.884, 1.019) | 0.911 (0.879, 0.946) |  | 1.756 (1.630, 1.891) | 1.006 (0.965, 1.048) |
|  | CVD male | 1.000 (0.965, 1.037) | 0.928 (0.911, 0.946) |  | 1.526 (1.470, 1.584) | 0.979 (0.960, 0.999) |
|  | CVD female | 1.035 (0.999, 1.072) | 0.954 (0.936, 0.972) |  | 1.810 (1.749, 1.874) | 1.021 (1.001, 1.041) |
|  | Stroke male | 0.969 (0.924, 1.016) | 0.925 (0.902, 0.948) |  | 1.463 (1.392, 1.537) | 0.944 (0.920, 0.969) |
|  | Stroke female | 0.968 (0.921, 1.015) | 0.924 (0.901, 0.948) |  | 1.704 (1.626, 1.785) | 0.999 (0.973, 1.025) |
|  | IHD male | 0.999 (0.933, 1.068) | 0.882 (0.852, 0.914) |  | 1.643 (1.532, 1.759) | 1.014 (0.976, 1.053) |
|  | IHD female | 1.018 (0.953, 1.085) | 0.937 (0.906, 0.970) |  | 1.951 (1.832, 2.076) | 1.036 (0.998, 1.074) |
|  | MI male | 1.156 (1.057, 1.262) | 0.950 (0.905, 0.998) |  | 1.624 (1.477, 1.781) | 1.057 (1.004, 1.113) |
|  | MI female | 1.126 (1.026, 1.233) | 0.979 (0.931, 1.030) |  | 1.916 (1.749, 2.095) | 1.091 (1.034, 1.150) |
|  | Nonaccidental Edu1 | 0.772 (0.605, 0.970) | 1.073 (0.963, 1.199) |  | 0.669 (0.492, 0.889) | 0.948 (0.855, 1.051) |
|  | Nonaccidental Edu2 | 0.855 (0.748, 0.973) | 0.981 (0.922, 1.045) |  | 0.961 (0.823, 1.117) | 0.961 (0.903, 1.023) |
|  | Nonaccidental Edu3 | 0.860 (0.835, 0.885) | 0.924 (0.911, 0.937) |  | 1.057 (1.023, 1.092) | 0.972 (0.959, 0.986) |
|  | RESP Edu1 | 1.028 (0.523, 1.816) | 1.343 (0.960, 1.923) |  | 0.652 (0.225, 1.493) | 1.134 (0.824, 1.556) |
|  | RESP Edu2 | 1.397 (0.971, 1.948) | 1.044 (0.850, 1.291) |  | 0.643 (0.336, 1.119) | 1.029 (0.828, 1.275) |
|  | RESP Edu3 | 0.912 (0.845, 0.984) | 0.919 (0.885, 0.954) |  | 1.105 (1.003, 1.214) | 0.944 (0.906, 0.984) |
|  | COPD Edu1 | 0.687 (0.167, 1.855) | 1.226 (0.731, 2.172) |  | 0.282 (0.016, 1.361) | 1.252 (0.755, 2.059) |
|  | COPD Edu2 | 0.958 (0.881, 1.041) | 0.897 (0.860, 0.936) |  | 1.144 (1.026, 1.271) | 0.938 (0.896, 0.983) |
|  | COPD Edu3 | 1.596 (0.981, 2.460) | 1.001 (0.757, 1.339) |  | 0.485 (0.147, 1.184) | 0.917 (0.667, 1.251) |
|  | CVD Edu1 | 0.816 (0.514, 1.227) | 0.979 (0.805, 1.199) |  | 0.817 (0.457, 1.354) | 0.900 (0.736, 1.099) |
|  | CVD Edu2 | 0.815 (0.639, 1.023) | 0.992 (0.889, 1.109) |  | 1.026 (0.769, 1.341) | 0.871 (0.775, 0.977) |
|  | CVD Edu3 | 0.899 (0.859, 0.940) | 0.937 (0.917, 0.958) |  | 1.117 (1.059, 1.176) | 0.993 (0.971, 1.016) |
|  | Stroke Edu1 | 1.049 (0.565, 1.779) | 1.056 (0.789, 1.436) |  | 0.552 (0.192, 1.250) | 0.885 (0.657, 1.187) |
|  | Stroke Edu2 | 0.797 (0.561, 1.096) | 1.051 (0.900, 1.233) |  | 1.099 (0.742, 1.576) | 0.880 (0.747, 1.034) |
|  | Stroke Edu3 | 0.875 (0.823, 0.929) | 0.966 (0.938, 0.995) |  | 1.099 (1.024, 1.178) | 1.005 (0.975, 1.036) |
|  | IHD Edu1 | 0.518 (0.183, 1.146) | 0.993 (0.711, 1.412) |  | 0.877 (0.334, 1.907) | 0.878 (0.627, 1.224) |
|  | IHD Edu2 | 0.884 (0.572, 1.304) | 0.938 (0.769, 1.151) |  | 0.693 (0.372, 1.181) | 0.818 (0.662, 1.007) |
|  | IHD Edu3 | 0.982 (0.902, 1.067) | 0.915 (0.877, 0.955) |  | 1.187 (1.072, 1.311) | 0.997 (0.954, 1.042) |
|  | MI Edu1 | 0.584 (0.142, 1.587) | 0.837 (0.542, 1.327) |  | 0.423 (0.068, 1.410) | 1.111 (0.716, 1.709) |
|  | MI Edu2 | 0.778 (0.419, 1.318) | 0.798 (0.617, 1.041) |  | 0.679 (0.301, 1.324) | 0.971 (0.743, 1.265) |
|  | MI Edu3 | 1.008 (0.901, 1.124) | 0.879 (0.831, 0.931) |  | 1.223 (1.066, 1.398) | 1.010 (0.952, 1.072) |

*Edu1 refers to college; Edu2 refers to high school; Edu3 refers to middle school and lower

Table S3 Pooled relative risk (RR) of total and cause-specific mortalities associated with temperature extremes (< 5^th^/> 95^th^ percentile of mean temperature) and PM_2.5_ pollution (> 35 μg m^-3^) in 13 cities of Jiangsu Province, 2015-2019

| City | Outcome | Cold Seasons | |  | Hot Seasons | |
| --- | --- | --- | --- | --- | --- | --- |
|  |  | RR of cold extremes (95% CI) | RR of PM_2.5_ pollution (95% CI) |  | RR of hot extremes (95% CI) | RR of PM_2.5_ pollution (95% CI) |
| Changzhou | Total | 1.146 (1.108, 1.184) | 1.040 (1.017, 1.063) |  | 1.278 (1.231, 1.327) | 1.025 (1.007, 1.044) |
|  | Non-accidental | 1.144 (1.104, 1.184) | 1.038 (1.014, 1.062) |  | 1.229 (1.180, 1.279) | 1.022 (1.002, 1.042) |
|  | RESP | 1.250 (1.138, 1.370) | 1.094 (1.025, 1.168) |  | 1.279 (1.128, 1.446) | 1.018 (0.957, 1.082) |
|  | COPD | 1.219 (1.081, 1.369) | 1.095 (1.010, 1.190) |  | 1.309 (1.114, 1.530) | 1.005 (0.929, 1.087) |
|  | CVD | 1.191 (1.127, 1.258) | 1.054 (1.016, 1.094) |  | 1.365 (1.280, 1.455) | 1.011 (0.979, 1.044) |
|  | Stroke | 1.172 (1.089, 1.260) | 1.057 (1.007, 1.111) |  | 1.342 (1.233, 1.459) | 1.021 (0.979, 1.065) |
|  | IHD | 1.206 (1.087, 1.334) | 1.046 (0.976, 1.122) |  | 1.340 (1.184, 1.512) | 0.999 (0.939, 1.062) |
|  | MI | 1.214 (1.025, 1.426) | 1.084 (0.968, 1.217) |  | 1.367 (1.118, 1.657) | 1.057 (0.958, 1.166) |
| Huaian | Total | 1.101 (1.066, 1.136) | 1.068 (1.047, 1.088) |  | 1.274 (1.234, 1.316) | 1.036 (1.019, 1.054) |
|  | Non-accidental | 1.110 (1.074, 1.146) | 1.067 (1.047, 1.089) |  | 1.244 (1.202, 1.286) | 1.038 (1.021, 1.057) |
|  | RESP | 1.215 (1.128, 1.306) | 1.123 (1.072, 1.177) |  | 1.299 (1.191, 1.414) | 1.055 (1.009, 1.103) |
|  | COPD | 1.227 (1.137, 1.322) | 1.127 (1.074, 1.183) |  | 1.311 (1.198, 1.432) | 1.066 (1.018, 1.116) |
|  | CVD | 1.125 (1.068, 1.184) | 1.059 (1.027, 1.093) |  | 1.381 (1.309, 1.456) | 1.055 (1.025, 1.085) |
|  | Stroke | 1.148 (1.077, 1.223) | 1.052 (1.012, 1.093) |  | 1.372 (1.283, 1.466) | 1.048 (1.011, 1.086) |
|  | IHD | 1.061 (0.957, 1.174) | 1.079 (1.015, 1.148) |  | 1.450 (1.308, 1.603) | 1.089 (1.029, 1.151) |
|  | MI | 1.034 (0.900, 1.182) | 1.073 (0.991, 1.163) |  | 1.370 (1.194, 1.565) | 1.113 (1.036, 1.197) |
| Lianyungang | Total | 1.190 (1.152, 1.228) | 1.069 (1.050, 1.087) |  | 1.229 (1.186, 1.274) | 1.019 (1.000, 1.039) |
|  | Non-accidental | 1.188 (1.149, 1.228) | 1.066 (1.047, 1.085) |  | 1.194 (1.149, 1.241) | 1.014 (0.993, 1.034) |
|  | RESP | 1.269 (1.170, 1.374) | 1.089 (1.041, 1.139) |  | 1.168 (1.043, 1.304) | 1.098 (1.036, 1.163) |
|  | COPD | 1.223 (1.104, 1.352) | 1.078 (1.020, 1.140) |  | 1.278 (1.114, 1.459) | 1.062 (0.988, 1.142) |
|  | CVD | 1.218 (1.157, 1.280) | 1.067 (1.038, 1.097) |  | 1.307 (1.232, 1.386) | 1.003 (0.971, 1.035) |
|  | Stroke | 1.200 (1.122, 1.282) | 1.080 (1.042, 1.120) |  | 1.263 (1.166, 1.365) | 1.004 (0.962, 1.047) |
|  | IHD | 1.242 (1.139, 1.351) | 1.041 (0.994, 1.091) |  | 1.331 (1.203, 1.470) | 1.002 (0.948, 1.059) |
|  | MI | 1.305 (1.173, 1.448) | 1.036 (0.977, 1.098) |  | 1.293 (1.137, 1.463) | 0.988 (0.921, 1.058) |
| Nanjing | Total | 1.120 (1.090, 1.150) | 1.056 (1.040, 1.073) |  | 1.252 (1.214, 1.290) | 1.027 (1.011, 1.044) |
|  | Non-accidental | 1.124 (1.093, 1.155) | 1.057 (1.040, 1.074) |  | 1.222 (1.184, 1.261) | 1.026 (1.010, 1.043) |
|  | RESP | 1.305 (1.208, 1.409) | 1.087 (1.036, 1.141) |  | 1.289 (1.160, 1.428) | 1.007 (0.954, 1.063) |
|  | COPD | 1.355 (1.222, 1.497) | 1.089 (1.022, 1.162) |  | 1.286 (1.116, 1.477) | 1.034 (0.961, 1.113) |
|  | CVD | 1.183 (1.136, 1.231) | 1.059 (1.034, 1.084) |  | 1.371 (1.309, 1.435) | 1.033 (1.008, 1.059) |
|  | Stroke | 1.171 (1.111, 1.232) | 1.061 (1.029, 1.094) |  | 1.395 (1.316, 1.478) | 1.029 (0.997, 1.063) |
|  | IHD | 1.245 (1.154, 1.341) | 1.030 (0.984, 1.077) |  | 1.281 (1.167, 1.403) | 1.008 (0.960, 1.059) |
|  | MI | 1.181 (1.043, 1.333) | 1.034 (0.962, 1.113) |  | 1.291 (1.114, 1.490) | 0.980 (0.907, 1.059) |
| Nantong | Total | 1.176 (1.151, 1.202) | 1.059 (1.047, 1.070) |  | 1.394 (1.362, 1.426) | 1.027 (1.014, 1.040) |
|  | Non-accidental | 1.180 (1.154, 1.207) | 1.059 (1.047, 1.071) |  | 1.338 (1.306, 1.370) | 1.026 (1.013, 1.040) |
|  | RESP | 1.332 (1.265, 1.402) | 1.095 (1.065, 1.126) |  | 1.484 (1.394, 1.579) | 1.050 (1.014, 1.087) |
|  | COPD | 1.313 (1.242, 1.388) | 1.090 (1.058, 1.123) |  | 1.470 (1.374, 1.573) | 1.051 (1.012, 1.091) |
|  | CVD | 1.242 (1.199, 1.285) | 1.062 (1.042, 1.081) |  | 1.535 (1.477, 1.594) | 1.040 (1.017, 1.063) |
|  | Stroke | 1.241 (1.186, 1.298) | 1.065 (1.040, 1.090) |  | 1.471 (1.399, 1.545) | 1.021 (0.993, 1.050) |
|  | IHD | 1.244 (1.168, 1.324) | 1.051 (1.018, 1.086) |  | 1.675 (1.565, 1.792) | 1.071 (1.029, 1.114) |
|  | MI | 1.257 (1.138, 1.384) | 1.030 (0.979, 1.085) |  | 1.606 (1.442, 1.786) | 1.119 (1.051, 1.191) |
| Suqian | Total | 1.147 (1.113, 1.181) | 1.077 (1.054, 1.101) |  | 1.176 (1.137, 1.216) | 1.043 (1.025, 1.060) |
|  | Non-accidental | 1.152 (1.118, 1.187) | 1.079 (1.055, 1.103) |  | 1.176 (1.135, 1.217) | 1.045 (1.027, 1.064) |
|  | RESP | 1.339 (1.207, 1.480) | 1.192 (1.098, 1.298) |  | 1.248 (1.089, 1.423) | 1.134 (1.059, 1.213) |
|  | COPD | 1.340 (1.192, 1.502) | 1.217 (1.108, 1.341) |  | 1.246 (1.068, 1.445) | 1.160 (1.075, 1.253) |
|  | CVD | 1.198 (1.152, 1.246) | 1.081 (1.050, 1.114) |  | 1.252 (1.194, 1.311) | 1.053 (1.028, 1.079) |
|  | Stroke | 1.207 (1.144, 1.272) | 1.052 (1.011, 1.096) |  | 1.214 (1.137, 1.294) | 1.058 (1.023, 1.093) |
|  | IHD | 1.220 (1.138, 1.306) | 1.110 (1.053, 1.171) |  | 1.250 (1.149, 1.357) | 1.018 (0.975, 1.063) |
|  | MI | 1.233 (1.137, 1.334) | 1.078 (1.014, 1.146) |  | 1.276 (1.157, 1.403) | 1.039 (0.988, 1.093) |
| Suzhou | Total | 1.153 (1.125, 1.182) | 1.062 (1.048, 1.077) |  | 1.205 (1.171, 1.240) | 1.023 (1.008, 1.039) |
|  | Non-accidental | 1.162 (1.132, 1.192) | 1.067 (1.051, 1.083) |  | 1.167 (1.132, 1.203) | 1.022 (1.006, 1.038) |
|  | RESP | 1.308 (1.223, 1.396) | 1.146 (1.101, 1.192) |  | 1.179 (1.078, 1.288) | 1.042 (0.995, 1.091) |
|  | COPD | 1.324 (1.216, 1.439) | 1.130 (1.075, 1.189) |  | 1.169 (1.041, 1.310) | 1.070 (1.008, 1.135) |
|  | CVD | 1.153 (1.104, 1.203) | 1.071 (1.045, 1.097) |  | 1.233 (1.170, 1.298) | 1.057 (1.029, 1.086) |
|  | Stroke | 1.155 (1.093, 1.220) | 1.060 (1.028, 1.093) |  | 1.167 (1.090, 1.247) | 1.059 (1.023, 1.097) |
|  | IHD | 1.129 (1.014, 1.253) | 1.057 (0.997, 1.122) |  | 1.269 (1.116, 1.438) | 1.109 (1.037, 1.187) |
|  | MI | 1.202 (1.022, 1.404) | 1.080 (0.986, 1.185) |  | 1.323 (1.089, 1.594) | 1.178 (1.062, 1.305) |
| Taizhou | Total | 1.160 (1.126, 1.195) | 1.045 (1.026, 1.064) |  | 1.535 (1.492, 1.580) | 1.044 (1.028, 1.061) |
|  | Non-accidental | 1.161 (1.126, 1.197) | 1.044 (1.025, 1.063) |  | 1.401 (1.358, 1.445) | 1.044 (1.027, 1.062) |
|  | RESP | 1.217 (1.122, 1.318) | 1.083 (1.030, 1.140) |  | 1.503 (1.377, 1.639) | 1.070 (1.018, 1.125) |
|  | COPD | 1.220 (1.117, 1.330) | 1.086 (1.029, 1.148) |  | 1.533 (1.395, 1.683) | 1.060 (1.004, 1.119) |
|  | CVD | 1.213 (1.156, 1.272) | 1.051 (1.020, 1.082) |  | 1.699 (1.620, 1.782) | 1.040 (1.011, 1.069) |
|  | Stroke | 1.136 (1.061, 1.214) | 1.042 (1.001, 1.085) |  | 1.548 (1.446, 1.655) | 1.031 (0.992, 1.071) |
|  | IHD | 1.270 (1.164, 1.383) | 1.033 (0.980, 1.090) |  | 1.962 (1.807, 2.128) | 1.043 (0.991, 1.097) |
|  | MI | 1.306 (1.172, 1.450) | 1.032 (0.964, 1.105) |  | 1.938 (1.752, 2.141) | 1.028 (0.965, 1.094) |
| Wuxi | Total | 1.129 (1.096, 1.162) | 1.030 (1.012, 1.048) |  | 1.152 (1.114, 1.192) | 1.022 (1.005, 1.039) |
|  | Non-accidental | 1.130 (1.096, 1.165) | 1.032 (1.012, 1.051) |  | 1.103 (1.063, 1.144) | 1.018 (1.001, 1.036) |
|  | RESP | 1.305 (1.200, 1.417) | 1.047 (0.992, 1.106) |  | 1.173 (1.044, 1.314) | 1.022 (0.966, 1.080) |
|  | COPD | 1.331 (1.208, 1.464) | 1.029 (0.967, 1.097) |  | 1.242 (1.088, 1.413) | 1.058 (0.992, 1.129) |
|  | CVD | 1.135 (1.077, 1.194) | 1.045 (1.012, 1.079) |  | 1.137 (1.067, 1.211) | 1.034 (1.002, 1.066) |
|  | Stroke | 1.136 (1.063, 1.213) | 1.070 (1.028, 1.115) |  | 1.071 (0.986, 1.162) | 1.034 (0.994, 1.075) |
|  | IHD | 1.191 (1.071, 1.319) | 1.076 (1.008, 1.150) |  | 1.254 (1.100, 1.425) | 1.076 (1.009, 1.149) |
|  | MI | 1.214 (0.998, 1.463) | 1.092 (0.965, 1.240) |  | 1.141 (0.884, 1.452) | 1.147 (1.017, 1.293) |
| Xuzhou | Total | 1.088 (1.064, 1.112) | 1.054 (1.031, 1.077) |  | 1.120 (1.092, 1.148) | 1.033 (1.020, 1.046) |
|  | Non-accidental | 1.088 (1.063, 1.113) | 1.060 (1.037, 1.085) |  | 1.107 (1.078, 1.137) | 1.034 (1.020, 1.047) |
|  | RESP | 1.144 (1.079, 1.212) | 1.148 (1.083, 1.219) |  | 1.113 (1.033, 1.198) | 1.060 (1.023, 1.098) |
|  | COPD | 1.141 (1.071, 1.215) | 1.120 (1.051, 1.194) |  | 1.102 (1.015, 1.194) | 1.069 (1.029, 1.111) |
|  | CVD | 1.107 (1.073, 1.142) | 1.073 (1.041, 1.107) |  | 1.177 (1.135, 1.221) | 1.037 (1.018, 1.056) |
|  | Stroke | 1.078 (1.030, 1.127) | 1.067 (1.021, 1.116) |  | 1.123 (1.064, 1.183) | 1.026 (0.999, 1.052) |
|  | IHD | 1.168 (1.115, 1.224) | 1.071 (1.021, 1.123) |  | 1.229 (1.161, 1.299) | 1.052 (1.023, 1.082) |
|  | MI | 1.149 (1.088, 1.214) | 1.080 (1.022, 1.141) |  | 1.227 (1.149, 1.308) | 1.039 (1.006, 1.073) |
| Yancheng | Total | 1.093 (1.067, 1.119) | 1.042 (1.030, 1.055) |  | 1.434 (1.400, 1.469) | 1.036 (1.021, 1.051) |
|  | Non-accidental | 1.100 (1.073, 1.127) | 1.041 (1.028, 1.054) |  | 1.401 (1.366, 1.436) | 1.036 (1.020, 1.051) |
|  | RESP | 1.187 (1.124, 1.251) | 1.047 (1.018, 1.077) |  | 1.579 (1.487, 1.676) | 1.026 (0.989, 1.065) |
|  | COPD | 1.181 (1.117, 1.247) | 1.046 (1.017, 1.077) |  | 1.603 (1.507, 1.704) | 1.021 (0.983, 1.061) |
|  | CVD | 1.100 (1.056, 1.145) | 1.042 (1.021, 1.063) |  | 1.595 (1.532, 1.661) | 1.028 (1.003, 1.055) |
|  | Stroke | 1.105 (1.050, 1.163) | 1.046 (1.020, 1.073) |  | 1.594 (1.514, 1.677) | 1.015 (0.983, 1.048) |
|  | IHD | 1.104 (1.024, 1.188) | 1.022 (0.985, 1.061) |  | 1.661 (1.540, 1.789) | 1.042 (0.994, 1.092) |
|  | MI | 1.125 (1.026, 1.231) | 1.008 (0.962, 1.056) |  | 1.570 (1.426, 1.724) | 1.037 (0.978, 1.099) |
| Yangzhou | Total | 1.113 (1.080, 1.147) | 1.063 (1.044, 1.083) |  | 1.544 (1.501, 1.588) | 1.029 (1.013, 1.046) |
|  | Non-accidental | 1.116 (1.082, 1.151) | 1.060 (1.041, 1.081) |  | 1.434 (1.391, 1.477) | 1.022 (1.005, 1.039) |
|  | RESP | 1.169 (1.074, 1.270) | 1.086 (1.030, 1.146) |  | 1.499 (1.366, 1.641) | 1.037 (0.984, 1.093) |
|  | COPD | 1.198 (1.089, 1.313) | 1.084 (1.021, 1.151) |  | 1.537 (1.387, 1.701) | 1.043 (0.983, 1.105) |
|  | CVD | 1.173 (1.120, 1.227) | 1.059 (1.029, 1.089) |  | 1.774 (1.698, 1.853) | 1.023 (0.997, 1.050) |
|  | Stroke | 1.181 (1.111, 1.255) | 1.068 (1.028, 1.110) |  | 1.717 (1.620, 1.820) | 1.002 (0.968, 1.037) |
|  | IHD | 1.144 (1.045, 1.249) | 1.014 (0.960, 1.072) |  | 1.819 (1.669, 1.981) | 1.035 (0.983, 1.090) |
|  | MI | 1.083 (0.954, 1.223) | 0.986 (0.916, 1.063) |  | 1.679 (1.484, 1.895) | 1.012 (0.942, 1.086) |
| Zhenjiang | Total | 1.128 (1.084, 1.173) | 1.046 (1.021, 1.072) |  | 1.304 (1.251, 1.359) | 1.038 (1.017, 1.060) |
|  | Non-accidental | 1.135 (1.090, 1.182) | 1.046 (1.020, 1.073) |  | 1.261 (1.207, 1.317) | 1.035 (1.013, 1.057) |
|  | RESP | 1.277 (1.136, 1.432) | 1.126 (1.043, 1.218) |  | 1.329 (1.148, 1.531) | 1.034 (0.962, 1.111) |
|  | COPD | 1.336 (1.165, 1.524) | 1.119 (1.022, 1.227) |  | 1.295 (1.087, 1.532) | 1.025 (0.941, 1.116) |
|  | CVD | 1.189 (1.117, 1.264) | 1.053 (1.011, 1.096) |  | 1.520 (1.422, 1.623) | 1.036 (1.001, 1.073) |
|  | Stroke | 1.117 (1.027, 1.212) | 1.063 (1.009, 1.120) |  | 1.514 (1.389, 1.647) | 1.045 (0.999, 1.092) |
|  | IHD | 1.233 (1.100, 1.377) | 1.057 (0.982, 1.139) |  | 1.522 (1.340, 1.723) | 0.995 (0.931, 1.063) |
|  | MI | 1.226 (1.042, 1.431) | 1.085 (0.978, 1.207) |  | 1.510 (1.254, 1.806) | 0.968 (0.879, 1.066) |

Table S4 The relative excess risk due to interaction (RERI) of temperature extremes and PM_2.5_ pollution in three regions of Jiangsu, 2015-2019

| Region | Outcome | Cold Seasons |  | Hot Seasons |
| --- | --- | --- | --- | --- |
|  |  | RERI (95% CI) |  | RERI (95% CI) |
| Southern | Total | 0.052 (0.015, 0.089) |  | 0.239 (0.198, 0.279) |
|  | Non-accidental | 0.040 (0.001, 0.079) |  | 0.202 (0.161, 0.243) |
|  | Stroke | 0.020 (-0.063, 0.103) |  | 0.306 (0.215, 0.398) |
|  | CVD | 0.003 (-0.062, 0.069) |  | 0.285 (0.212, 0.358) |
|  | RESP | 0.048 (-0.072, 0.168) |  | 0.267 (0.135, 0.399) |
|  | IHD | -0.130 (-0.275, 0.016) |  | 0.167 (0.016, 0.319) |
|  | MI | -0.016 (-0.251, 0.220) |  | 0.217 (-0.025, 0.460) |
|  | COPD | 0.055 (-0.095, 0.205) |  | 0.315 (0.144, 0.487) |
|  | Nonaccidental 0to64 | 0.098 (-0.005, 0.200) |  | 0.078 (-0.020, 0.176) |
|  | Nonaccidental 65to74 | -0.028 (-0.118, 0.062) |  | 0.139 (0.051, 0.227) |
|  | Nonaccidental 75above | 0.046 (-0.001, 0.094) |  | 0.254 (0.201, 0.307) |
|  | RESP 0to64 | -0.091 (-0.654, 0.472) |  | 0.007 (-0.539, 0.553) |
|  | RESP 65to74 | -0.051 (-0.449, 0.348) |  | 0.496 (0.056, 0.937) |
|  | RESP 75above | 0.069 (-0.060, 0.197) |  | 0.259 (0.116, 0.402) |
|  | CVD 0to64 | -0.029 (-0.245, 0.187) |  | 0.163 (-0.060, 0.386) |
|  | CVD 65to74 | -0.131 (-0.310, 0.048) |  | 0.407 (0.220, 0.595) |
|  | CVD 75above | 0.030 (-0.044, 0.104) |  | 0.279 (0.195, 0.364) |
|  | Total 0to64 | 0.109 (0.015, 0.202) |  | 0.154 (0.062, 0.246) |
|  | Total 65to74 | -0.011 (-0.097, 0.074) |  | 0.158 (0.072, 0.245) |
|  | Total 75above | 0.056 (0.011, 0.102) |  | 0.289 (0.237, 0.341) |
|  | IHD 0to64 | -0.058 (-0.492, 0.376) |  | 0.325 (-0.149, 0.798) |
|  | IHD 65to74 | -0.158 (-0.569, 0.253) |  | 0.247 (-0.143, 0.637) |
|  | IHD 75above | -0.134 (-0.301, 0.033) |  | 0.130 (-0.045, 0.306) |
|  | MI 0to64 | 0.068 (-0.480, 0.616) |  | 0.603 (0.018, 1.188) |
|  | MI 65to74 | 0.141 (-0.393, 0.675) |  | -0.027 (-0.530, 0.477) |
|  | MI 75above | -0.075 (-0.372, 0.223) |  | 0.180 (-0.131, 0.491) |
|  | Stroke 0to64 | -0.047 (-0.339, 0.246) |  | 0.100 (-0.190, 0.391) |
|  | Stroke 65to74 | -0.139 (-0.364, 0.086) |  | 0.505 (0.271, 0.739) |
|  | Stroke 75above | 0.055 (-0.038, 0.149) |  | 0.293 (0.187, 0.400) |
|  | COPD 0to64 | 0.288 (-0.426, 1.002) |  | -0.022 (-0.939, 0.896) |
|  | COPD 65to74 | -0.056 (-0.561, 0.449) |  | 0.648 (0.051, 1.245) |
|  | COPD 75above | 0.060 (-0.101, 0.221) |  | 0.293 (0.111, 0.476) |
|  | Nonaccidental Edu1 | -0.265 (-0.717, 0.187) |  | 0.110 (-0.295, 0.514) |
|  | Nonaccidental Edu2 | -0.365 (-0.623, -0.107) |  | 0.184 (-0.055, 0.423) |
|  | Nonaccidental Edu3 | -0.093 (-0.139, -0.046) |  | 0.318 (0.262, 0.373) |
|  | CVD Edu1 | -0.697 (-1.612, 0.217) |  | -0.329 (-1.137, 0.478) |
|  | CVD Edu2 | -0.302 (-0.733, 0.130) |  | 0.207 (-0.240, 0.655) |
|  | CVD Edu3 | -0.070 (-0.141, 0.001) |  | 0.286 (0.193, 0.380) |
|  | Stroke Edu1 | -0.548 (-1.766, 0.669) |  | -0.337 (-1.638, 0.964) |
|  | Stroke Edu2 | -0.340 (-0.972, 0.293) |  | 0.350 (-0.315, 1.014) |
|  | Stroke Edu3 | -0.127 (-0.220, -0.035) |  | 0.305 (0.184, 0.427) |
|  | IHD Edu1 | -0.551 (-1.955, 0.854) |  | -0.458 (-2.002, 1.086) |
|  | IHD Edu2 | -0.352 (-1.090, 0.387) |  | -0.013 (-0.832, 0.806) |
|  | IHD Edu3 | 0.070 (-0.060, 0.200) |  | 0.316 (0.135, 0.497) |
|  | MI Edu1 | 0.144 (-1.498, 1.785) |  | 0.318 (-1.576, 2.212) |
|  | MI Edu2 | -0.354 (-1.271, 0.563) |  | 0.114 (-1.086, 1.314) |
|  | MI Edu3 | 0.115 (-0.043, 0.274) |  | 0.387 (0.156, 0.618) |
|  | RESP Edu1 | -0.975 (-2.289, 0.339) |  | -0.628 (-1.816, 0.560) |
|  | RESP Edu2 | -0.238 (-1.155, 0.678) |  | 0.359 (-0.462, 1.179) |
|  | RESP Edu3 | -0.187 (-0.314, -0.060) |  | 0.467 (0.310, 0.625) |
|  | COPD Edu1 | -1.442 (-3.565, 0.680) |  | -0.803 (-2.051, 0.444) |
|  | COPD Edu2 | -0.408 (-1.631, 0.815) |  | 0.564 (0.387, 0.742) |
|  | COPD Edu3 | -0.112 (-0.244, 0.019) |  | 0.728 (-0.364, 1.819) |
|  | Nonaccidental male | 0.049 (-0.003, 0.101) |  | 0.172 (0.118, 0.225) |
|  | Nonaccidental female | 0.030 (-0.029, 0.088) |  | 0.241 (0.176, 0.305) |
|  | CVD male | 0.010 (-0.082, 0.101) |  | 0.220 (0.120, 0.320) |
|  | CVD female | -0.003 (-0.096, 0.090) |  | 0.351 (0.244, 0.457) |
|  | Stroke male | 0.044 (-0.071, 0.160) |  | 0.230 (0.103, 0.357) |
|  | Stroke female | -0.005 (-0.123, 0.113) |  | 0.384 (0.251, 0.517) |
|  | IHD male | -0.100 (-0.298, 0.098) |  | 0.131 (-0.072, 0.334) |
|  | IHD female | -0.162 (-0.376, 0.053) |  | 0.206 (-0.021, 0.433) |
|  | MI male | -0.004 (-0.303, 0.295) |  | 0.215 (-0.092, 0.522) |
|  | MI female | -0.033 (-0.415, 0.348) |  | 0.218 (-0.172, 0.609) |
|  | RESP male | 0.039 (-0.115, 0.193) |  | 0.156 (-0.008, 0.321) |
|  | RESP female | 0.061 (-0.130, 0.251) |  | 0.425 (0.206, 0.644) |
|  | COPD male | 0.066 (-0.123, 0.255) |  | 0.207 (-0.006, 0.419) |
|  | COPD female | 0.037 (-0.210, 0.284) |  | 0.491 (0.201, 0.782) |
| Northern | Total | 0.121 (0.084, 0.158) |  | 0.145 (0.107, 0.183) |
|  | Non-accidental | 0.124 (0.085, 0.162) |  | 0.136 (0.097, 0.176) |
|  | Stroke | 0.160 (0.081, 0.239) |  | 0.135 (0.051, 0.219) |
|  | CVD | 0.213 (0.151, 0.275) |  | 0.101 (0.036, 0.166) |
|  | RESP | 0.063 (-0.032, 0.158) |  | 0.217 (0.102, 0.332) |
|  | IHD | 0.306 (0.191, 0.421) |  | 0.017 (-0.099, 0.133) |
|  | MI | 0.299 (0.155, 0.443) |  | -0.005 (-0.144, 0.134) |
|  | COPD | 0.047 (-0.055, 0.148) |  | 0.265 (0.140, 0.391) |
|  | Nonaccidental 0to64 | 0.098 (0.001, 0.195) |  | 0.074 (-0.014, 0.161) |
|  | Nonaccidental 65to74 | 0.010 (-0.085, 0.106) |  | 0.100 (0.012, 0.189) |
|  | Nonaccidental 75above | 0.132 (0.078, 0.187) |  | 0.210 (0.153, 0.267) |
|  | RESP 0to64 | -0.102 (-0.557, 0.353) |  | -0.013 (-0.438, 0.413) |
|  | RESP 65to74 | -0.127 (-0.444, 0.190) |  | 0.228 (-0.094, 0.549) |
|  | RESP 75above | 0.047 (-0.067, 0.162) |  | 0.261 (0.123, 0.399) |
|  | CVD 0to64 | 0.255 (0.073, 0.438) |  | -0.022 (-0.194, 0.149) |
|  | CVD 65to74 | 0.157 (-0.014, 0.327) |  | 0.034 (-0.124, 0.192) |
|  | CVD 75above | 0.216 (0.133, 0.299) |  | 0.163 (0.077, 0.248) |
|  | Total 0to64 | 0.102 (0.013, 0.190) |  | 0.070 (-0.009, 0.150) |
|  | Total 65to74 | 0.013 (-0.080, 0.105) |  | 0.103 (0.016, 0.189) |
|  | Total 75above | 0.125 (0.072, 0.178) |  | 0.231 (0.174, 0.288) |
|  | IHD 0to64 | 0.181 (-0.138, 0.500) |  | -0.110 (-0.398, 0.178) |
|  | IHD 65to74 | 0.284 (-0.069, 0.638) |  | -0.103 (-0.395, 0.190) |
|  | IHD 75above | 0.348 (0.194, 0.502) |  | 0.070 (-0.084, 0.224) |
|  | MI 0to64 | 0.116 (-0.243, 0.475) |  | -0.145 (-0.463, 0.173) |
|  | MI 65to74 | 0.361 (-0.057, 0.779) |  | -0.006 (-0.346, 0.335) |
|  | MI 75above | 0.357 (0.159, 0.555) |  | 0.021 (-0.169, 0.211) |
|  | Stroke 0to64 | 0.386 (0.144, 0.629) |  | 0.084 (-0.151, 0.320) |
|  | Stroke 65to74 | 0.055 (-0.151, 0.262) |  | 0.071 (-0.131, 0.273) |
|  | Stroke 75above | 0.145 (0.040, 0.250) |  | 0.201 (0.090, 0.311) |
|  | COPD 0to64 | -0.141 (-0.672, 0.391) |  | -0.092 (-0.619, 0.434) |
|  | COPD 65to74 | -0.107 (-0.426, 0.212) |  | 0.313 (-0.037, 0.664) |
|  | COPD 75above | 0.048 (-0.071, 0.167) |  | 0.291 (0.144, 0.438) |
|  | Nonaccidental Edu1 | 0.288 (0.047, 0.529) |  | -0.183 (-0.412, 0.045) |
|  | Nonaccidental Edu2 | 0.053 (-0.118, 0.224) |  | -0.031 (-0.202, 0.141) |
|  | Nonaccidental Edu3 | -0.054 (-0.098, -0.010) |  | 0.093 (0.046, 0.141) |
|  | CVD Edu1 | 0.297 (-0.099, 0.693) |  | -0.036 (-0.473, 0.401) |
|  | CVD Edu2 | -0.009 (-0.307, 0.289) |  | -0.109 (-0.419, 0.201) |
|  | CVD Edu3 | 0.043 (-0.028, 0.113) |  | 0.090 (0.012, 0.168) |
|  | Stroke Edu1 | 0.092 (-0.561, 0.744) |  | -0.061 (-0.733, 0.610) |
|  | Stroke Edu2 | -0.118 (-0.549, 0.313) |  | -0.367 (-0.778, 0.043) |
|  | Stroke Edu3 | -0.006 (-0.096, 0.084) |  | 0.096 (-0.004, 0.195) |
|  | IHD Edu1 | 0.406 (-0.117, 0.928) |  | -0.115 (-0.787, 0.556) |
|  | IHD Edu2 | 0.167 (-0.323, 0.657) |  | 0.082 (-0.493, 0.657) |
|  | IHD Edu3 | 0.118 (-0.014, 0.250) |  | 0.071 (-0.077, 0.218) |
|  | MI Edu1 | 0.326 (-0.415, 1.066) |  | -0.741 (-1.796, 0.314) |
|  | MI Edu2 | 0.133 (-0.548, 0.814) |  | 0.328 (-0.515, 1.171) |
|  | MI Edu3 | 0.296 (0.100, 0.493) |  | 0.207 (-0.006, 0.420) |
|  | RESP Edu1 | 0.097 (-0.802, 0.995) |  | -0.244 (-0.909, 0.421) |
|  | RESP Edu2 | 0.102 (-0.430, 0.634) |  | -0.099 (-0.814, 0.616) |
|  | RESP Edu3 | -0.219 (-0.333, -0.104) |  | 0.165 (0.025, 0.305) |
|  | COPD Edu1 | 0.218 (-0.556, 0.991) |  | -0.427 (-1.917, 1.062) |
|  | COPD Edu2 | 0.170 (-0.545, 0.886) |  | 0.168 (0.008, 0.329) |
|  | COPD Edu3 | -0.242 (-0.370, -0.113) |  | 0.070 (-1.071, 1.211) |
|  | Nonaccidental male | 0.099 (0.046, 0.152) |  | 0.125 (0.073, 0.176) |
|  | Nonaccidental female | 0.153 (0.096, 0.209) |  | 0.151 (0.089, 0.213) |
|  | CVD male | 0.163 (0.074, 0.252) |  | 0.100 (0.012, 0.189) |
|  | CVD female | 0.264 (0.177, 0.351) |  | 0.102 (0.007, 0.197) |
|  | Stroke male | 0.129 (0.017, 0.242) |  | 0.121 (0.007, 0.234) |
|  | Stroke female | 0.193 (0.082, 0.305) |  | 0.149 (0.025, 0.274) |
|  | IHD male | 0.244 (0.075, 0.412) |  | 0.014 (-0.147, 0.175) |
|  | IHD female | 0.364 (0.208, 0.521) |  | 0.021 (-0.147, 0.188) |
|  | MI male | 0.257 (0.048, 0.466) |  | -0.038 (-0.228, 0.151) |
|  | MI female | 0.342 (0.143, 0.541) |  | 0.031 (-0.174, 0.236) |
|  | RESP male | 0.031 (-0.101, 0.164) |  | 0.210 (0.061, 0.359) |
|  | RESP female | 0.098 (-0.039, 0.234) |  | 0.224 (0.046, 0.402) |
|  | COPD male | 0.028 (-0.112, 0.169) |  | 0.237 (0.074, 0.400) |
|  | COPD female | 0.067 (-0.079, 0.214) |  | 0.296 (0.101, 0.491) |
| Middle | Total | 0.068 (0.032, 0.105) |  | 0.620 (0.571, 0.669) |
|  | Non-accidental | 0.063 (0.026, 0.101) |  | 0.500 (0.451, 0.550) |
|  | Stroke | 0.033 (-0.045, 0.112) |  | 0.633 (0.520, 0.746) |
|  | CVD | 0.072 (0.011, 0.134) |  | 0.756 (0.666, 0.846) |
|  | RESP | 0.026 (-0.072, 0.124) |  | 0.667 (0.517, 0.816) |
|  | IHD | 0.137 (0.029, 0.246) |  | 1.131 (0.954, 1.309) |
|  | MI | 0.039 (-0.138, 0.217) |  | 1.087 (0.835, 1.338) |
|  | COPD | 0.031 (-0.074, 0.136) |  | 0.732 (0.569, 0.894) |
|  | Nonaccidental 0to64 | 0.042 (-0.056, 0.141) |  | 0.266 (0.157, 0.376) |
|  | Nonaccidental 65to74 | 0.016 (-0.075, 0.107) |  | 0.282 (0.180, 0.384) |
|  | Nonaccidental 75above | 0.080 (0.035, 0.126) |  | 0.634 (0.570, 0.699) |
|  | RESP 0to64 | 0.000 (-0.297, 0.296) |  | 0.763 (0.244, 1.281) |
|  | RESP 65to74 | 0.120 (-0.126, 0.366) |  | 0.736 (0.367, 1.105) |
|  | RESP 75above | 0.020 (-0.094, 0.133) |  | 0.650 (0.477, 0.822) |
|  | CVD 0to64 | 0.047 (-0.121, 0.215) |  | 0.694 (0.460, 0.928) |
|  | CVD 65to74 | 0.064 (-0.089, 0.218) |  | 0.584 (0.377, 0.791) |
|  | CVD 75above | 0.082 (0.009, 0.154) |  | 0.814 (0.705, 0.924) |
|  | Total 0to64 | 0.056 (-0.037, 0.148) |  | 0.345 (0.241, 0.449) |
|  | Total 65to74 | 0.017 (-0.070, 0.105) |  | 0.393 (0.292, 0.494) |
|  | Total 75above | 0.085 (0.041, 0.129) |  | 0.776 (0.711, 0.841) |
|  | IHD 0to64 | 0.273 (0.012, 0.535) |  | 0.776 (0.306, 1.245) |
|  | IHD 65to74 | 0.142 (-0.138, 0.423) |  | 1.036 (0.633, 1.439) |
|  | IHD 75above | 0.121 (-0.008, 0.251) |  | 1.219 (1.003, 1.435) |
|  | MI 0to64 | 0.278 (-0.096, 0.651) |  | 0.380 (-0.240, 1.001) |
|  | MI 65to74 | 0.157 (-0.273, 0.587) |  | 0.798 (0.286, 1.309) |
|  | MI 75above | -0.035 (-0.257, 0.188) |  | 1.308 (0.988, 1.629) |
|  | Stroke 0to64 | -0.047 (-0.277, 0.182) |  | 0.730 (0.428, 1.033) |
|  | Stroke 65to74 | 0.044 (-0.152, 0.240) |  | 0.534 (0.270, 0.799) |
|  | Stroke 75above | 0.045 (-0.047, 0.137) |  | 0.648 (0.511, 0.785) |
|  | COPD 0to64 | 0.161 (-0.129, 0.450) |  | 0.929 (0.372, 1.485) |
|  | COPD 65to74 | 0.076 (-0.196, 0.348) |  | 0.816 (0.425, 1.207) |
|  | COPD 75above | 0.022 (-0.099, 0.144) |  | 0.705 (0.516, 0.894) |
|  | Nonaccidental Edu1 | 0.184 (-0.276, 0.644) |  | -0.366 (-0.877, 0.146) |
|  | Nonaccidental Edu2 | -0.107 (-0.391, 0.177) |  | 0.092 (-0.235, 0.420) |
|  | Nonaccidental Edu3 | -0.047 (-0.108, 0.014) |  | 0.158 (0.080, 0.236) |
|  | CVD Edu1 | 0.148 (-0.707, 1.002) |  | 0.029 (-0.954, 1.011) |
|  | CVD Edu2 | -0.253 (-0.759, 0.253) |  | -0.290 (-0.939, 0.359) |
|  | CVD Edu3 | -0.030 (-0.129, 0.070) |  | 0.047 (-0.090, 0.183) |
|  | Stroke Edu1 | 0.301 (-1.193, 1.795) |  | 0.828 (0.103, 1.553) |
|  | Stroke Edu2 | -0.612 (-1.390, 0.166) |  | -0.390 (-1.351, 0.572) |
|  | Stroke Edu3 | 0.001 (-0.131, 0.133) |  | 0.001 (-0.181, 0.183) |
|  | IHD Edu1 | -0.187 (-1.429, 1.056) |  | 0.401 (-1.045, 1.846) |
|  | IHD Edu2 | -0.225 (-1.160, 0.710) |  | 0.299 (-0.464, 1.062) |
|  | IHD Edu3 | -0.078 (-0.277, 0.121) |  | 0.086 (-0.187, 0.359) |
|  | MI Edu1 | 0.077 (-1.493, 1.648) |  | 0.450 (-0.554, 1.454) |
|  | MI Edu2 | -0.322 (-1.434, 0.791) |  | -0.338 (-1.584, 0.908) |
|  | MI Edu3 | -0.151 (-0.422, 0.120) |  | 0.075 (-0.307, 0.457) |
|  | RESP Edu1 | -0.310 (-2.499, 1.879) |  | -1.547 (-3.792, 0.697) |
|  | RESP Edu2 | -0.096 (-1.424, 1.233) |  | 0.637 (-0.094, 1.369) |
|  | RESP Edu3 | -0.145 (-0.319, 0.030) |  | 0.424 (0.205, 0.643) |
|  | COPD Edu1 | -2.365 (-6.435, 1.704) |  | -1.630 (-4.459, 1.200) |
|  | COPD Edu2 | -0.949 (-3.091, 1.193) |  | 0.484 (0.235, 0.732) |
|  | COPD Edu3 | -0.128 (-0.324, 0.069) |  | 0.213 (-0.842, 1.267) |
|  | Nonaccidental male | 0.044 (-0.007, 0.095) |  | 0.398 (0.335, 0.461) |
|  | Nonaccidental female | 0.087 (0.031, 0.142) |  | 0.627 (0.549, 0.705) |
|  | CVD male | 0.065 (-0.020, 0.151) |  | 0.691 (0.571, 0.811) |
|  | CVD female | 0.080 (-0.008, 0.168) |  | 0.824 (0.689, 0.959) |
|  | Stroke male | 0.012 (-0.098, 0.123) |  | 0.616 (0.467, 0.766) |
|  | Stroke female | 0.055 (-0.057, 0.166) |  | 0.653 (0.482, 0.824) |
|  | IHD male | 0.152 (0.000, 0.304) |  | 0.997 (0.757, 1.237) |
|  | IHD female | 0.124 (-0.031, 0.278) |  | 1.261 (1.000, 1.522) |
|  | MI male | 0.167 (-0.074, 0.408) |  | 1.023 (0.693, 1.353) |
|  | MI female | -0.101 (-0.363, 0.162) |  | 1.152 (0.768, 1.536) |
|  | RESP male | -0.033 (-0.165, 0.099) |  | 0.544 (0.356, 0.731) |
|  | RESP female | 0.110 (-0.036, 0.256) |  | 0.834 (0.589, 1.079) |
|  | COPD male | -0.009 (-0.148, 0.130) |  | 0.579 (0.380, 0.778) |
|  | COPD female | 0.092 (-0.068, 0.251) |  | 0.946 (0.671, 1.221) |

*Edu1 refers to college; Edu2 refers to high school; Edu3 refers to middle school and lower

Table S5 Differences between RERIs of extremely hot and cold temperatures and PM_2.5_ pollution in 13 cities of Jiangsu, 2015 to 2019

| City | Outcome | RERI cold | RERI hot | Delta RERI |
| --- | --- | --- | --- | --- |
| Changzhou | Total | -0.013 | 0.391 | 0.404 |
|  | Non-accidental | -0.010 | 0.287 | 0.298 |
|  | RESP | -0.054 | 0.293 | 0.347 |
|  | COPD | -0.235 | 0.225 | 0.460 |
|  | CVD | -0.013 | 0.533 | 0.547 |
|  | Stroke | 0.082 | 0.480 | 0.398 |
|  | IHD | -0.271 | 0.495 | 0.765 |
|  | MI | -0.184 | 0.447 | 0.632 |
| Huaian | Total | 0.031 | 0.342 | 0.310 |
|  | Non-accidental | 0.044 | 0.325 | 0.282 |
|  | RESP | 0.050 | 0.549 | 0.498 |
|  | COPD | 0.019 | 0.502 | 0.484 |
|  | CVD | 0.033 | 0.480 | 0.447 |
|  | Stroke | 0.133 | 0.457 | 0.324 |
|  | IHD | -0.146 | 0.575 | 0.721 |
|  | MI | -0.193 | 0.577 | 0.770 |
| Lianyungang | Total | 0.071 | 0.150 | 0.079 |
|  | Non-accidental | 0.069 | 0.117 | 0.048 |
|  | RESP | 0.171 | 0.077 | -0.094 |
|  | COPD | 0.067 | 0.117 | 0.050 |
|  | CVD | 0.048 | 0.272 | 0.224 |
|  | Stroke | 0.062 | 0.142 | 0.080 |
|  | IHD | -0.046 | 0.405 | 0.451 |
|  | MI | -0.157 | 0.466 | 0.624 |
| Nanjing | Total | -0.032 | 0.228 | 0.260 |
|  | Non-accidental | -0.034 | 0.198 | 0.233 |
|  | RESP | -0.042 | 0.045 | 0.088 |
|  | COPD | 0.029 | 0.150 | 0.121 |
|  | CVD | -0.024 | 0.396 | 0.420 |
|  | Stroke | 0.025 | 0.389 | 0.363 |
|  | IHD | -0.082 | 0.246 | 0.327 |
|  | MI | 0.018 | 0.150 | 0.133 |
| Nantong | Total | 0.034 | 0.296 | 0.263 |
|  | Non-accidental | 0.028 | 0.243 | 0.214 |
|  | RESP | 0.038 | 0.205 | 0.166 |
|  | COPD | 0.030 | 0.216 | 0.186 |
|  | CVD | 0.013 | 0.451 | 0.438 |
|  | Stroke | -0.021 | 0.387 | 0.409 |
|  | IHD | 0.060 | 0.601 | 0.541 |
|  | MI | 0.016 | 0.520 | 0.505 |
| Suqian | Total | -0.106 | 0.281 | 0.387 |
|  | Non-accidental | -0.109 | 0.285 | 0.395 |
|  | RESP | 0.133 | 0.318 | 0.184 |
|  | COPD | 0.211 | 0.344 | 0.133 |
|  | CVD | -0.095 | 0.414 | 0.508 |
|  | Stroke | -0.027 | 0.430 | 0.457 |
|  | IHD | -0.108 | 0.347 | 0.456 |
|  | MI | -0.181 | 0.381 | 0.562 |
| Suzhou | Total | -0.054 | 0.162 | 0.216 |
|  | Non-accidental | -0.060 | 0.129 | 0.189 |
|  | RESP | -0.165 | 0.143 | 0.308 |
|  | COPD | -0.113 | 0.197 | 0.310 |
|  | CVD | -0.084 | 0.214 | 0.298 |
|  | Stroke | -0.074 | 0.260 | 0.334 |
|  | IHD | -0.160 | 0.067 | 0.227 |
|  | MI | -0.240 | 0.281 | 0.521 |
| Taizhou | Total | 0.006 | 0.435 | 0.430 |
|  | Non-accidental | 0.018 | 0.321 | 0.303 |
|  | RESP | -0.108 | 0.423 | 0.531 |
|  | COPD | -0.135 | 0.493 | 0.628 |
|  | CVD | 0.080 | 0.565 | 0.484 |
|  | Stroke | -0.005 | 0.560 | 0.566 |
|  | IHD | 0.172 | 0.645 | 0.472 |
|  | MI | 0.093 | 0.556 | 0.464 |
| Wuxi | Total | -0.041 | 0.092 | 0.132 |
|  | Non-accidental | -0.060 | 0.069 | 0.129 |
|  | RESP | -0.275 | 0.081 | 0.357 |
|  | COPD | -0.270 | 0.224 | 0.494 |
|  | CVD | 0.005 | 0.030 | 0.025 |
|  | Stroke | -0.027 | 0.115 | 0.142 |
|  | IHD | 0.024 | -0.060 | -0.084 |
|  | MI | 0.476 | -0.067 | -0.543 |
| Xuzhou | Total | 0.174 | 0.096 | -0.078 |
|  | Non-accidental | 0.174 | 0.081 | -0.093 |
|  | RESP | 0.073 | 0.187 | 0.114 |
|  | COPD | 0.121 | 0.179 | 0.058 |
|  | CVD | 0.197 | 0.058 | -0.139 |
|  | Stroke | 0.167 | 0.036 | -0.131 |
|  | IHD | 0.220 | 0.074 | -0.145 |
|  | MI | 0.259 | 0.068 | -0.191 |
| Yancheng | Total | 0.028 | 0.143 | 0.115 |
|  | Non-accidental | 0.026 | 0.129 | 0.103 |
|  | RESP | 0.081 | 0.122 | 0.042 |
|  | COPD | 0.079 | 0.133 | 0.054 |
|  | CVD | -0.036 | 0.140 | 0.176 |
|  | Stroke | -0.049 | 0.120 | 0.170 |
|  | IHD | -0.079 | 0.187 | 0.266 |
|  | MI | -0.092 | 0.275 | 0.367 |
| Yangzhou | Total | -0.051 | 0.451 | 0.502 |
|  | Non-accidental | -0.068 | 0.282 | 0.350 |
|  | RESP | -0.236 | 0.212 | 0.448 |
|  | COPD | -0.199 | 0.299 | 0.498 |
|  | CVD | -0.011 | 0.640 | 0.651 |
|  | Stroke | -0.052 | 0.498 | 0.550 |
|  | IHD | 0.061 | 0.945 | 0.883 |
|  | MI | -0.067 | 0.994 | 1.060 |
| Zhenjiang | Total | -0.062 | 0.275 | 0.337 |
|  | Non-accidental | -0.071 | 0.221 | 0.292 |
|  | RESP | -0.078 | 0.222 | 0.300 |
|  | COPD | -0.115 | 0.186 | 0.301 |
|  | CVD | -0.164 | 0.460 | 0.624 |
|  | Stroke | -0.254 | 0.556 | 0.809 |
|  | IHD | -0.073 | 0.343 | 0.415 |
|  | MI | -0.109 | 0.334 | 0.444 |

Table S6 Sensitive analysis results of the relative excess risk due to interaction (RERI) of extreme temperatures and PM_2.5_ using '*DOW*' instead of '*Holiday*' in three regions of Jiangsu, 2015-2019

| Region | Outcome | Cold Seasons |  | Hot Seasons |
| --- | --- | --- | --- | --- |
|  |  | RERI (95% CI) |  | RERI (95% CI) |
| Southern | Total | 0.059 (0.022, 0.096) |  | 0.242 (0.201, 0.282) |
|  | Non-accidental | 0.048 (0.009, 0.087) |  | 0.205 (0.164, 0.247) |
|  | Stroke | 0.031 (-0.051, 0.114) |  | 0.311 (0.219, 0.403) |
|  | CVD | 0.016 (-0.049, 0.081) |  | 0.288 (0.215, 0.361) |
|  | RESP | 0.060 (-0.059, 0.179) |  | 0.271 (0.139, 0.403) |
|  | IHD | -0.115 (-0.260, 0.030) |  | 0.169 (0.016, 0.321) |
|  | MI | 0.004 (-0.231, 0.239) |  | 0.215 (-0.028, 0.458) |
|  | COPD | 0.066 (-0.083, 0.215) |  | 0.317 (0.145, 0.488) |
|  | Nonaccidental 0to64 | 0.103 (0.001, 0.205) |  | 0.080 (-0.018, 0.178) |
|  | Nonaccidental 65to74 | -0.021 (-0.110, 0.069) |  | 0.139 (0.051, 0.227) |
|  | Nonaccidental 75above | 0.055 (0.008, 0.103) |  | 0.259 (0.206, 0.312) |
|  | RESP 0to64 | -0.090 (-0.651, 0.470) |  | 0.013 (-0.537, 0.562) |
|  | RESP 65to74 | -0.023 (-0.416, 0.369) |  | 0.479 (0.041, 0.917) |
|  | RESP 75above | 0.079 (-0.048, 0.207) |  | 0.265 (0.121, 0.408) |
|  | CVD 0to64 | -0.021 (-0.238, 0.195) |  | 0.158 (-0.066, 0.381) |
|  | CVD 65to74 | -0.114 (-0.292, 0.064) |  | 0.404 (0.216, 0.592) |
|  | CVD 75above | 0.042 (-0.031, 0.116) |  | 0.285 (0.200, 0.370) |
|  | Total 0to64 | 0.113 (0.019, 0.207) |  | 0.156 (0.063, 0.248) |
|  | Total 65to74 | -0.006 (-0.092, 0.079) |  | 0.158 (0.071, 0.244) |
|  | Total 75above | 0.064 (0.018, 0.109) |  | 0.293 (0.241, 0.345) |
|  | IHD 0to64 | -0.049 (-0.482, 0.385) |  | 0.310 (-0.163, 0.784) |
|  | IHD 65to74 | -0.138 (-0.545, 0.269) |  | 0.243 (-0.149, 0.635) |
|  | IHD 75above | -0.119 (-0.286, 0.047) |  | 0.135 (-0.041, 0.311) |
|  | MI 0to64 | 0.081 (-0.464, 0.627) |  | 0.576 (-0.007, 1.158) |
|  | MI 65to74 | 0.158 (-0.369, 0.686) |  | -0.030 (-0.538, 0.478) |
|  | MI 75above | -0.052 (-0.349, 0.244) |  | 0.185 (-0.126, 0.496) |
|  | Stroke 0to64 | -0.031 (-0.324, 0.262) |  | 0.096 (-0.196, 0.388) |
|  | Stroke 65to74 | -0.122 (-0.345, 0.102) |  | 0.501 (0.266, 0.736) |
|  | Stroke 75above | 0.065 (-0.029, 0.158) |  | 0.301 (0.194, 0.407) |
|  | COPD 0to64 | 0.277 (-0.437, 0.990) |  | -0.010 (-0.918, 0.898) |
|  | COPD 65to74 | -0.036 (-0.533, 0.460) |  | 0.636 (0.043, 1.229) |
|  | COPD 75above | 0.072 (-0.088, 0.232) |  | 0.295 (0.112, 0.478) |
|  | Nonaccidental Edu1 | -0.255 (-0.708, 0.197) |  | 0.111 (-0.297, 0.518) |
|  | Nonaccidental Edu2 | -0.360 (-0.619, -0.102) |  | 0.184 (-0.055, 0.424) |
|  | Nonaccidental Edu3 | -0.085 (-0.131, -0.038) |  | 0.319 (0.263, 0.374) |
|  | CVD Edu1 | -0.641 (-1.538, 0.257) |  | -0.321 (-1.134, 0.491) |
|  | CVD Edu2 | -0.285 (-0.716, 0.145) |  | 0.217 (-0.230, 0.664) |
|  | CVD Edu3 | -0.062 (-0.134, 0.009) |  | 0.288 (0.194, 0.381) |
|  | Stroke Edu1 | -0.489 (-1.673, 0.695) |  | -0.317 (-1.649, 1.016) |
|  | Stroke Edu2 | -0.329 (-0.962, 0.304) |  | 0.362 (-0.301, 1.025) |
|  | Stroke Edu3 | -0.119 (-0.212, -0.026) |  | 0.307 (0.185, 0.428) |
|  | IHD Edu1 | -0.556 (-1.970, 0.858) |  | -0.421 (-1.938, 1.095) |
|  | IHD Edu2 | -0.327 (-1.060, 0.405) |  | -0.011 (-0.836, 0.814) |
|  | IHD Edu3 | 0.073 (-0.057, 0.203) |  | 0.317 (0.135, 0.498) |
|  | MI Edu1 | 0.143 (-1.507, 1.792) |  | 0.357 (-1.504, 2.219) |
|  | MI Edu2 | -0.329 (-1.237, 0.579) |  | 0.131 (-1.079, 1.340) |
|  | MI Edu3 | 0.117 (-0.042, 0.275) |  | 0.389 (0.158, 0.620) |
|  | RESP Edu1 | -0.869 (-2.128, 0.389) |  | -0.633 (-1.816, 0.549) |
|  | RESP Edu2 | -0.213 (-1.125, 0.700) |  | 0.347 (-0.473, 1.168) |
|  | RESP Edu3 | -0.170 (-0.297, -0.044) |  | 0.471 (0.313, 0.629) |
|  | COPD Edu1 | -1.262 (-3.210, 0.686) |  | -0.779 (-1.988, 0.430) |
|  | COPD Edu2 | -0.099 (-0.230, 0.031) |  | 0.569 (0.391, 0.747) |
|  | COPD Edu3 | -0.387 (-1.608, 0.835) |  | 0.700 (-0.384, 1.784) |
|  | Nonaccidental male | 0.058 (0.006, 0.110) |  | 0.174 (0.120, 0.228) |
|  | Nonaccidental female | 0.036 (-0.023, 0.094) |  | 0.245 (0.180, 0.309) |
|  | CVD male | 0.024 (-0.067, 0.115) |  | 0.222 (0.122, 0.322) |
|  | CVD female | 0.007 (-0.085, 0.100) |  | 0.355 (0.249, 0.462) |
|  | Stroke male | 0.059 (-0.056, 0.174) |  | 0.233 (0.106, 0.361) |
|  | Stroke female | 0.002 (-0.116, 0.120) |  | 0.390 (0.256, 0.523) |
|  | IHD male | -0.086 (-0.283, 0.112) |  | 0.131 (-0.072, 0.334) |
|  | IHD female | -0.147 (-0.361, 0.067) |  | 0.209 (-0.019, 0.437) |
|  | MI male | 0.007 (-0.292, 0.306) |  | 0.208 (-0.099, 0.515) |
|  | MI female | -0.001 (-0.379, 0.376) |  | 0.223 (-0.169, 0.615) |
|  | RESP male | 0.054 (-0.099, 0.206) |  | 0.156 (-0.008, 0.320) |
|  | RESP female | 0.069 (-0.121, 0.259) |  | 0.436 (0.217, 0.655) |
|  | COPD male | 0.077 (-0.110, 0.265) |  | 0.206 (-0.006, 0.418) |
|  | COPD female | 0.048 (-0.198, 0.293) |  | 0.497 (0.206, 0.788) |
| Northern | Total | 0.120 (0.083, 0.157) |  | 0.144 (0.106, 0.182) |
|  | Non-accidental | 0.122 (0.084, 0.161) |  | 0.135 (0.096, 0.175) |
|  | Stroke | 0.159 (0.080, 0.238) |  | 0.130 (0.046, 0.214) |
|  | CVD | 0.210 (0.149, 0.272) |  | 0.098 (0.033, 0.163) |
|  | RESP | 0.062 (-0.032, 0.156) |  | 0.214 (0.099, 0.329) |
|  | IHD | 0.304 (0.190, 0.418) |  | 0.017 (-0.099, 0.133) |
|  | MI | 0.293 (0.149, 0.436) |  | -0.006 (-0.145, 0.134) |
|  | COPD | 0.047 (-0.053, 0.148) |  | 0.263 (0.137, 0.389) |
|  | Nonaccidental 0to64 | 0.096 (0.000, 0.193) |  | 0.075 (-0.013, 0.163) |
|  | Nonaccidental 65to74 | 0.006 (-0.089, 0.102) |  | 0.101 (0.012, 0.190) |
|  | Nonaccidental 75above | 0.133 (0.078, 0.187) |  | 0.208 (0.151, 0.266) |
|  | RESP 0to64 | -0.096 (-0.546, 0.354) |  | -0.006 (-0.435, 0.422) |
|  | RESP 65to74 | -0.133 (-0.448, 0.181) |  | 0.218 (-0.104, 0.539) |
|  | RESP 75above | 0.051 (-0.063, 0.164) |  | 0.256 (0.118, 0.395) |
|  | CVD 0to64 | 0.247 (0.066, 0.429) |  | -0.022 (-0.193, 0.150) |
|  | CVD 65to74 | 0.152 (-0.018, 0.322) |  | 0.031 (-0.127, 0.190) |
|  | CVD 75above | 0.216 (0.134, 0.298) |  | 0.160 (0.074, 0.246) |
|  | Total 0to64 | 0.101 (0.012, 0.189) |  | 0.071 (-0.008, 0.150) |
|  | Total 65to74 | 0.010 (-0.083, 0.102) |  | 0.104 (0.018, 0.190) |
|  | Total 75above | 0.126 (0.073, 0.179) |  | 0.230 (0.173, 0.287) |
|  | IHD 0to64 | 0.172 (-0.143, 0.487) |  | -0.106 (-0.393, 0.181) |
|  | IHD 65to74 | 0.279 (-0.075, 0.632) |  | -0.098 (-0.391, 0.195) |
|  | IHD 75above | 0.349 (0.197, 0.502) |  | 0.070 (-0.084, 0.225) |
|  | MI 0to64 | 0.105 (-0.250, 0.460) |  | -0.140 (-0.456, 0.177) |
|  | MI 65to74 | 0.355 (-0.063, 0.773) |  | -0.001 (-0.341, 0.339) |
|  | MI 75above | 0.354 (0.157, 0.551) |  | 0.020 (-0.170, 0.211) |
|  | Stroke 0to64 | 0.381 (0.139, 0.623) |  | 0.086 (-0.150, 0.323) |
|  | Stroke 65to74 | 0.051 (-0.155, 0.258) |  | 0.066 (-0.136, 0.267) |
|  | Stroke 75above | 0.147 (0.043, 0.251) |  | 0.196 (0.086, 0.307) |
|  | COPD 0to64 | -0.120 (-0.646, 0.405) |  | -0.081 (-0.613, 0.451) |
|  | COPD 65to74 | -0.113 (-0.430, 0.204) |  | 0.303 (-0.047, 0.653) |
|  | COPD 75above | 0.052 (-0.066, 0.170) |  | 0.287 (0.140, 0.434) |
|  | Nonaccidental Edu1 | 0.291 (0.050, 0.532) |  | -0.178 (-0.407, 0.051) |
|  | Nonaccidental Edu2 | 0.055 (-0.116, 0.225) |  | -0.037 (-0.208, 0.135) |
|  | Nonaccidental Edu3 | -0.054 (-0.099, -0.010) |  | 0.091 (0.043, 0.139) |
|  | CVD Edu1 | 0.300 (-0.093, 0.694) |  | -0.034 (-0.472, 0.404) |
|  | CVD Edu2 | -0.004 (-0.301, 0.293) |  | -0.113 (-0.423, 0.196) |
|  | CVD Edu3 | 0.041 (-0.029, 0.112) |  | 0.087 (0.009, 0.165) |
|  | Stroke Edu1 | 0.106 (-0.546, 0.758) |  | -0.044 (-0.720, 0.632) |
|  | Stroke Edu2 | -0.107 (-0.534, 0.321) |  | -0.371 (-0.781, 0.040) |
|  | Stroke Edu3 | -0.008 (-0.098, 0.082) |  | 0.095 (-0.005, 0.194) |
|  | IHD Edu1 | 0.414 (-0.093, 0.922) |  | -0.126 (-0.796, 0.544) |
|  | IHD Edu2 | 0.172 (-0.320, 0.665) |  | 0.068 (-0.504, 0.639) |
|  | IHD Edu3 | 0.115 (-0.018, 0.248) |  | 0.066 (-0.082, 0.213) |
|  | MI Edu1 | 0.336 (-0.390, 1.061) |  | -0.752 (-1.817, 0.312) |
|  | MI Edu2 | 0.142 (-0.545, 0.830) |  | 0.297 (-0.538, 1.132) |
|  | MI Edu3 | 0.292 (0.094, 0.490) |  | 0.204 (-0.009, 0.418) |
|  | RESP Edu1 | 0.153 (-0.715, 1.020) |  | -0.245 (-0.909, 0.419) |
|  | RESP Edu2 | 0.120 (-0.409, 0.649) |  | -0.144 (-0.851, 0.563) |
|  | RESP Edu3 | -0.214 (-0.327, -0.100) |  | 0.163 (0.024, 0.303) |
|  | COPD Edu1 | 0.224 (-0.554, 1.001) |  | -0.434 (-1.924, 1.056) |
|  | COPD Edu2 | -0.239 (-0.367, -0.111) |  | 0.168 (0.008, 0.327) |
|  | COPD Edu3 | 0.207 (-0.489, 0.903) |  | -0.005 (-1.127, 1.116) |
|  | Nonaccidental male | 0.097 (0.045, 0.150) |  | 0.124 (0.072, 0.175) |
|  | Nonaccidental female | 0.151 (0.095, 0.208) |  | 0.150 (0.088, 0.212) |
|  | CVD male | 0.161 (0.072, 0.249) |  | 0.096 (0.007, 0.184) |
|  | CVD female | 0.260 (0.174, 0.347) |  | 0.100 (0.005, 0.195) |
|  | Stroke male | 0.129 (0.017, 0.241) |  | 0.116 (0.002, 0.229) |
|  | Stroke female | 0.191 (0.080, 0.302) |  | 0.146 (0.021, 0.271) |
|  | IHD male | 0.240 (0.072, 0.407) |  | 0.011 (-0.150, 0.172) |
|  | IHD female | 0.363 (0.208, 0.519) |  | 0.023 (-0.144, 0.191) |
|  | MI male | 0.250 (0.042, 0.458) |  | -0.040 (-0.229, 0.149) |
|  | MI female | 0.336 (0.138, 0.534) |  | 0.032 (-0.172, 0.237) |
|  | RESP male | 0.029 (-0.103, 0.161) |  | 0.206 (0.057, 0.355) |
|  | RESP female | 0.098 (-0.036, 0.233) |  | 0.223 (0.045, 0.401) |
|  | COPD male | 0.027 (-0.112, 0.167) |  | 0.233 (0.070, 0.396) |
|  | COPD female | 0.070 (-0.076, 0.215) |  | 0.295 (0.100, 0.490) |
| Middle | Total | 0.087 (0.051, 0.123) |  | 0.627 (0.578, 0.676) |
|  | Non-accidental | 0.084 (0.047, 0.122) |  | 0.506 (0.457, 0.556) |
|  | Stroke | 0.057 (-0.021, 0.134) |  | 0.643 (0.529, 0.757) |
|  | CVD | 0.103 (0.042, 0.163) |  | 0.763 (0.673, 0.854) |
|  | RESP | 0.067 (-0.029, 0.163) |  | 0.676 (0.527, 0.826) |
|  | IHD | 0.176 (0.070, 0.283) |  | 1.130 (0.952, 1.307) |
|  | MI | 0.096 (-0.078, 0.270) |  | 1.089 (0.837, 1.341) |
|  | COPD | 0.073 (-0.030, 0.175) |  | 0.742 (0.579, 0.905) |
|  | Nonaccidental 0to64 | 0.063 (-0.035, 0.160) |  | 0.268 (0.159, 0.378) |
|  | Nonaccidental 65to74 | 0.022 (-0.069, 0.113) |  | 0.289 (0.187, 0.391) |
|  | Nonaccidental 75above | 0.106 (0.061, 0.151) |  | 0.641 (0.576, 0.706) |
|  | RESP 0to64 | 0.023 (-0.267, 0.313) |  | 0.795 (0.274, 1.316) |
|  | RESP 65to74 | 0.136 (-0.108, 0.379) |  | 0.763 (0.391, 1.134) |
|  | RESP 75above | 0.067 (-0.044, 0.179) |  | 0.653 (0.480, 0.826) |
|  | CVD 0to64 | 0.078 (-0.088, 0.243) |  | 0.697 (0.463, 0.932) |
|  | CVD 65to74 | 0.065 (-0.088, 0.217) |  | 0.596 (0.389, 0.804) |
|  | CVD 75above | 0.118 (0.047, 0.190) |  | 0.822 (0.712, 0.932) |
|  | Total 0to64 | 0.070 (-0.022, 0.162) |  | 0.348 (0.244, 0.452) |
|  | Total 65to74 | 0.022 (-0.065, 0.110) |  | 0.399 (0.298, 0.501) |
|  | Total 75above | 0.108 (0.064, 0.151) |  | 0.784 (0.718, 0.849) |
|  | IHD 0to64 | 0.279 (0.022, 0.537) |  | 0.766 (0.297, 1.234) |
|  | IHD 65to74 | 0.155 (-0.121, 0.432) |  | 1.050 (0.646, 1.455) |
|  | IHD 75above | 0.171 (0.044, 0.298) |  | 1.216 (1.000, 1.432) |
|  | MI 0to64 | 0.306 (-0.059, 0.671) |  | 0.373 (-0.248, 0.995) |
|  | MI 65to74 | 0.203 (-0.218, 0.623) |  | 0.809 (0.296, 1.321) |
|  | MI 75above | 0.031 (-0.187, 0.249) |  | 1.311 (0.990, 1.632) |
|  | Stroke 0to64 | -0.007 (-0.232, 0.218) |  | 0.743 (0.439, 1.047) |
|  | Stroke 65to74 | 0.039 (-0.157, 0.234) |  | 0.544 (0.279, 0.809) |
|  | Stroke 75above | 0.072 (-0.019, 0.164) |  | 0.659 (0.521, 0.796) |
|  | COPD 0to64 | 0.174 (-0.110, 0.458) |  | 0.963 (0.402, 1.524) |
|  | COPD 65to74 | 0.094 (-0.177, 0.364) |  | 0.844 (0.451, 1.237) |
|  | COPD 75above | 0.072 (-0.047, 0.190) |  | 0.708 (0.519, 0.898) |
|  | Nonaccidental Edu1 | 0.184 (-0.275, 0.643) |  | -0.361 (-0.872, 0.150) |
|  | Nonaccidental Edu2 | -0.090 (-0.374, 0.194) |  | 0.101 (-0.227, 0.430) |
|  | Nonaccidental Edu3 | -0.040 (-0.101, 0.021) |  | 0.154 (0.076, 0.232) |
|  | CVD Edu1 | 0.129 (-0.720, 0.978) |  | 0.041 (-0.949, 1.032) |
|  | CVD Edu2 | -0.223 (-0.722, 0.276) |  | -0.266 (-0.917, 0.386) |
|  | CVD Edu3 | -0.023 (-0.122, 0.076) |  | 0.046 (-0.090, 0.182) |
|  | Stroke Edu1 | 0.278 (-1.201, 1.756) |  | 0.794 (0.083, 1.505) |
|  | Stroke Edu2 | -0.618 (-1.401, 0.166) |  | -0.376 (-1.333, 0.580) |
|  | Stroke Edu3 | 0.002 (-0.130, 0.134) |  | 0.000 (-0.181, 0.182) |
|  | IHD Edu1 | -0.199 (-1.439, 1.041) |  | 0.458 (-1.018, 1.934) |
|  | IHD Edu2 | -0.159 (-1.051, 0.733) |  | 0.320 (-0.456, 1.096) |
|  | IHD Edu3 | -0.064 (-0.263, 0.134) |  | 0.079 (-0.194, 0.352) |
|  | MI Edu1 | 0.097 (-1.426, 1.621) |  | 0.497 (-0.582, 1.575) |
|  | MI Edu2 | -0.251 (-1.304, 0.802) |  | -0.327 (-1.606, 0.952) |
|  | MI Edu3 | -0.140 (-0.410, 0.130) |  | 0.060 (-0.322, 0.442) |
|  | RESP Edu1 | -0.332 (-2.493, 1.828) |  | -1.528 (-3.770, 0.714) |
|  | RESP Edu2 | -0.085 (-1.385, 1.215) |  | 0.640 (-0.092, 1.372) |
|  | RESP Edu3 | -0.139 (-0.313, 0.035) |  | 0.417 (0.197, 0.636) |
|  | COPD Edu1 | -2.501 (-6.766, 1.763) |  | -1.623 (-4.458, 1.212) |
|  | COPD Edu2 | -0.117 (-0.312, 0.079) |  | 0.485 (0.236, 0.735) |
|  | COPD Edu3 | -0.777 (-2.832, 1.279) |  | 0.200 (-0.837, 1.237) |
|  | Nonaccidental male | 0.066 (0.015, 0.117) |  | 0.402 (0.339, 0.465) |
|  | Nonaccidental female | 0.106 (0.051, 0.162) |  | 0.635 (0.556, 0.713) |
|  | CVD male | 0.097 (0.013, 0.181) |  | 0.697 (0.577, 0.818) |
|  | CVD female | 0.109 (0.022, 0.195) |  | 0.832 (0.697, 0.967) |
|  | Stroke male | 0.041 (-0.069, 0.150) |  | 0.623 (0.473, 0.773) |
|  | Stroke female | 0.073 (-0.037, 0.184) |  | 0.667 (0.495, 0.839) |
|  | IHD male | 0.187 (0.037, 0.336) |  | 0.998 (0.758, 1.239) |
|  | IHD female | 0.166 (0.014, 0.318) |  | 1.256 (0.995, 1.517) |
|  | MI male | 0.222 (-0.014, 0.459) |  | 1.028 (0.697, 1.358) |
|  | MI female | -0.041 (-0.298, 0.215) |  | 1.152 (0.768, 1.536) |
|  | RESP male | 0.011 (-0.118, 0.140) |  | 0.552 (0.364, 0.740) |
|  | RESP female | 0.145 (0.003, 0.288) |  | 0.845 (0.600, 1.091) |
|  | COPD male | 0.038 (-0.098, 0.174) |  | 0.586 (0.386, 0.785) |
|  | COPD female | 0.126 (-0.030, 0.281) |  | 0.961 (0.685, 1.237) |

*Edu1 refers to college; Edu2 refers to high school; Edu3 refers to middle school and lower

Table S7 Sensitive analysis results of the relative excess risk due to interaction (RERI) of extreme temperatures and PM_2.5_ pollution using natural cubic spline smooth function to the calendar day instead of '*year*' in three regions of Jiangsu, 2015-2019

| Region | Outcome | Cold Seasons |  | Hot Seasons |
| --- | --- | --- | --- | --- |
|  |  | RERI (95% CI) |  | RERI (95% CI) |
| Southern | Total | 0.043 (0.006, 0.080) |  | 0.182 (0.135, 0.230) |
|  | Non-accidental | 0.033 (-0.006, 0.071) |  | 0.150 (0.101, 0.199) |
|  | Stroke | 0.015 (-0.068, 0.098) |  | 0.206 (0.099, 0.314) |
|  | CVD | 0.004 (-0.060, 0.069) |  | 0.194 (0.108, 0.280) |
|  | RESP | 0.007 (-0.096, 0.110) |  | 0.230 (0.071, 0.389) |
|  | IHD | -0.111 (-0.256, 0.034) |  | 0.072 (-0.104, 0.249) |
|  | MI | -0.007 (-0.245, 0.231) |  | 0.128 (-0.145, 0.401) |
|  | COPD | 0.017 (-0.110, 0.144) |  | 0.280 (0.071, 0.490) |
|  | Nonaccidental 0to64 | 0.081 (-0.028, 0.189) |  | 0.046 (-0.069, 0.161) |
|  | Nonaccidental 65to74 | -0.026 (-0.119, 0.068) |  | 0.087 (-0.018, 0.193) |
|  | Nonaccidental 75above | 0.038 (-0.008, 0.084) |  | 0.196 (0.133, 0.259) |
|  | RESP 0to64 | -0.174 (-0.697, 0.349) |  | -0.169 (-0.700, 0.361) |
|  | RESP 65to74 | -0.081 (-0.425, 0.263) |  | 0.450 (-0.086, 0.986) |
|  | RESP 75above | 0.028 (-0.082, 0.138) |  | 0.233 (0.058, 0.408) |
|  | CVD 0to64 | -0.049 (-0.277, 0.178) |  | 0.068 (-0.198, 0.334) |
|  | CVD 65to74 | -0.125 (-0.311, 0.062) |  | 0.316 (0.090, 0.543) |
|  | CVD 75above | 0.031 (-0.041, 0.103) |  | 0.188 (0.089, 0.287) |
|  | Total 0to64 | 0.093 (-0.006, 0.193) |  | 0.119 (0.011, 0.226) |
|  | Total 65to74 | -0.012 (-0.102, 0.077) |  | 0.109 (0.006, 0.212) |
|  | Total 75above | 0.046 (0.002, 0.091) |  | 0.223 (0.161, 0.284) |
|  | IHD 0to64 | -0.080 (-0.515, 0.356) |  | 0.168 (-0.378, 0.713) |
|  | IHD 65to74 | -0.141 (-0.562, 0.280) |  | 0.185 (-0.270, 0.639) |
|  | IHD 75above | -0.110 (-0.276, 0.056) |  | 0.036 (-0.169, 0.241) |
|  | MI 0to64 | 0.028 (-0.521, 0.577) |  | 0.496 (-0.178, 1.169) |
|  | MI 65to74 | 0.109 (-0.416, 0.634) |  | -0.028 (-0.610, 0.555) |
|  | MI 75above | -0.044 (-0.347, 0.259) |  | 0.071 (-0.275, 0.416) |
|  | Stroke 0to64 | -0.072 (-0.389, 0.244) |  | 0.025 (-0.324, 0.374) |
|  | Stroke 65to74 | -0.136 (-0.373, 0.101) |  | 0.379 (0.100, 0.657) |
|  | Stroke 75above | 0.049 (-0.044, 0.141) |  | 0.193 (0.070, 0.316) |
|  | COPD 0to64 | 0.157 (-0.570, 0.884) |  | -0.289 (-1.126, 0.547) |
|  | COPD 65to74 | -0.081 (-0.496, 0.334) |  | 0.664 (-0.113, 1.441) |
|  | COPD 75above | 0.026 (-0.109, 0.162) |  | 0.266 (0.042, 0.490) |
|  | Nonaccidental Edu1 | -0.324 (-0.805, 0.158) |  | 0.065 (-0.452, 0.583) |
|  | Nonaccidental Edu2 | -0.352 (-0.639, -0.066) |  | 0.183 (-0.096, 0.462) |
|  | Nonaccidental Edu3 | -0.077 (-0.126, -0.028) |  | 0.268 (0.207, 0.329) |
|  | CVD Edu1 | -0.729 (-1.669, 0.210) |  | -0.337 (-1.338, 0.663) |
|  | CVD Edu2 | -0.277 (-0.745, 0.191) |  | 0.249 (-0.295, 0.792) |
|  | CVD Edu3 | -0.054 (-0.130, 0.022) |  | 0.207 (0.116, 0.299) |
|  | Stroke Edu1 | -0.591 (-1.796, 0.614) |  | -0.423 (-2.409, 1.564) |
|  | Stroke Edu2 | -0.263 (-0.955, 0.428) |  | 0.284 (-0.657, 1.225) |
|  | Stroke Edu3 | -0.107 (-0.206, -0.008) |  | 0.215 (0.095, 0.334) |
|  | IHD Edu1 | -0.615 (-2.132, 0.902) |  | -0.374 (-2.005, 1.257) |
|  | IHD Edu2 | -0.362 (-1.197, 0.473) |  | 0.092 (-0.810, 0.994) |
|  | IHD Edu3 | 0.084 (-0.061, 0.229) |  | 0.204 (0.035, 0.374) |
|  | MI Edu1 | -0.029 (-1.989, 1.930) |  | 0.329 (-1.384, 2.041) |
|  | MI Edu2 | -0.300 (-1.333, 0.733) |  | 0.234 (-1.147, 1.614) |
|  | MI Edu3 | 0.092 (-0.086, 0.269) |  | 0.193 (-0.012, 0.399) |
|  | RESP Edu1 | -0.661 (-1.840, 0.518) |  | -0.861 (-3.164, 1.442) |
|  | RESP Edu2 | -0.151 (-1.191, 0.890) |  | 0.402 (-0.658, 1.462) |
|  | RESP Edu3 | -0.158 (-0.280, -0.037) |  | 0.385 (0.215, 0.555) |
|  | COPD Edu1 | -0.933 (-2.661, 0.796) |  | -0.869 (-2.585, 0.847) |
|  | COPD Edu2 | -0.090 (-0.215, 0.035) |  | 0.447 (0.260, 0.635) |
|  | COPD Edu3 | -0.324 (-1.666, 1.018) |  | 0.860 (-0.688, 2.408) |
|  | Nonaccidental male | 0.040 (-0.013, 0.092) |  | 0.132 (0.068, 0.196) |
|  | Nonaccidental female | 0.024 (-0.033, 0.082) |  | 0.172 (0.096, 0.248) |
|  | CVD male | 0.004 (-0.088, 0.096) |  | 0.138 (0.021, 0.256) |
|  | CVD female | 0.004 (-0.086, 0.095) |  | 0.250 (0.125, 0.375) |
|  | Stroke male | 0.031 (-0.085, 0.148) |  | 0.141 (-0.009, 0.290) |
|  | Stroke female | -0.001 (-0.120, 0.117) |  | 0.271 (0.118, 0.425) |
|  | IHD male | -0.090 (-0.292, 0.111) |  | 0.065 (-0.173, 0.303) |
|  | IHD female | -0.133 (-0.344, 0.078) |  | 0.079 (-0.184, 0.341) |
|  | MI male | -0.005 (-0.309, 0.300) |  | 0.191 (-0.155, 0.538) |
|  | MI female | -0.013 (-0.392, 0.366) |  | 0.039 (-0.403, 0.480) |
|  | RESP male | -0.005 (-0.139, 0.130) |  | 0.130 (-0.064, 0.324) |
|  | RESP female | 0.023 (-0.138, 0.184) |  | 0.373 (0.101, 0.645) |
|  | COPD male | 0.018 (-0.142, 0.179) |  | 0.173 (-0.082, 0.429) |
|  | COPD female | 0.014 (-0.194, 0.222) |  | 0.453 (0.091, 0.815) |
| Northern | Total | 0.115 (0.078, 0.152) |  | 0.098 (0.056, 0.140) |
|  | Non-accidental | 0.117 (0.079, 0.155) |  | 0.095 (0.051, 0.139) |
|  | Stroke | 0.150 (0.073, 0.227) |  | 0.067 (-0.024, 0.158) |
|  | CVD | 0.188 (0.127, 0.248) |  | 0.023 (-0.047, 0.093) |
|  | RESP | 0.074 (-0.012, 0.159) |  | 0.161 (0.031, 0.292) |
|  | IHD | 0.255 (0.144, 0.367) |  | -0.079 (-0.202, 0.044) |
|  | MI | 0.235 (0.093, 0.376) |  | -0.109 (-0.255, 0.037) |
|  | COPD | 0.061 (-0.031, 0.152) |  | 0.217 (0.073, 0.362) |
|  | Nonaccidental 0to64 | 0.091 (-0.006, 0.189) |  | 0.068 (-0.034, 0.169) |
|  | Nonaccidental 65to74 | 0.006 (-0.091, 0.104) |  | 0.072 (-0.030, 0.174) |
|  | Nonaccidental 75above | 0.119 (0.067, 0.171) |  | 0.150 (0.085, 0.215) |
|  | RESP 0to64 | -0.056 (-0.447, 0.335) |  | -0.025 (-0.516, 0.467) |
|  | RESP 65to74 | -0.103 (-0.393, 0.186) |  | 0.159 (-0.200, 0.518) |
|  | RESP 75above | 0.062 (-0.039, 0.162) |  | 0.206 (0.046, 0.366) |
|  | CVD 0to64 | 0.206 (0.031, 0.381) |  | -0.075 (-0.266, 0.115) |
|  | CVD 65to74 | 0.132 (-0.042, 0.305) |  | -0.055 (-0.225, 0.115) |
|  | CVD 75above | 0.179 (0.100, 0.257) |  | 0.073 (-0.020, 0.167) |
|  | Total 0to64 | 0.093 (0.004, 0.182) |  | 0.056 (-0.035, 0.146) |
|  | Total 65to74 | 0.008 (-0.087, 0.103) |  | 0.069 (-0.029, 0.168) |
|  | Total 75above | 0.115 (0.064, 0.166) |  | 0.163 (0.099, 0.227) |
|  | IHD 0to64 | 0.136 (-0.173, 0.444) |  | -0.157 (-0.472, 0.158) |
|  | IHD 65to74 | 0.241 (-0.117, 0.599) |  | -0.201 (-0.513, 0.111) |
|  | IHD 75above | 0.268 (0.123, 0.413) |  | -0.047 (-0.212, 0.118) |
|  | MI 0to64 | 0.065 (-0.278, 0.407) |  | -0.210 (-0.554, 0.133) |
|  | MI 65to74 | 0.308 (-0.125, 0.741) |  | -0.131 (-0.494, 0.232) |
|  | MI 75above | 0.255 (0.065, 0.444) |  | -0.109 (-0.309, 0.091) |
|  | Stroke 0to64 | 0.329 (0.096, 0.561) |  | 0.027 (-0.233, 0.287) |
|  | Stroke 65to74 | 0.040 (-0.171, 0.250) |  | -0.018 (-0.234, 0.198) |
|  | Stroke 75above | 0.133 (0.034, 0.232) |  | 0.127 (0.004, 0.249) |
|  | COPD 0to64 | -0.109 (-0.613, 0.394) |  | -0.095 (-0.670, 0.480) |
|  | COPD 65to74 | -0.085 (-0.376, 0.205) |  | 0.251 (-0.150, 0.653) |
|  | COPD 75above | 0.064 (-0.041, 0.169) |  | 0.248 (0.076, 0.420) |
|  | Nonaccidental Edu1 | 0.273 (0.008, 0.539) |  | -0.259 (-0.539, 0.021) |
|  | Nonaccidental Edu2 | 0.064 (-0.118, 0.247) |  | -0.036 (-0.233, 0.161) |
|  | Nonaccidental Edu3 | 0.010 (-0.036, 0.056) |  | 0.104 (0.057, 0.151) |
|  | CVD Edu1 | 0.296 (-0.110, 0.702) |  | -0.149 (-0.704, 0.407) |
|  | CVD Edu2 | 0.011 (-0.305, 0.328) |  | -0.092 (-0.448, 0.263) |
|  | CVD Edu3 | 0.101 (0.029, 0.174) |  | 0.096 (0.021, 0.170) |
|  | Stroke Edu1 | 0.054 (-0.665, 0.774) |  | -0.066 (-1.039, 0.906) |
|  | Stroke Edu2 | -0.097 (-0.569, 0.375) |  | -0.365 (-0.852, 0.123) |
|  | Stroke Edu3 | 0.054 (-0.038, 0.145) |  | 0.094 (-0.002, 0.190) |
|  | IHD Edu1 | 0.456 (-0.036, 0.947) |  | -0.304 (-1.063, 0.456) |
|  | IHD Edu2 | 0.217 (-0.283, 0.717) |  | 0.033 (-0.582, 0.649) |
|  | IHD Edu3 | 0.175 (0.038, 0.312) |  | 0.094 (-0.045, 0.233) |
|  | MI Edu1 | 0.432 (-0.255, 1.120) |  | -0.885 (-2.143, 0.373) |
|  | MI Edu2 | 0.186 (-0.488, 0.859) |  | 0.181 (-0.722, 1.084) |
|  | MI Edu3 | 0.369 (0.160, 0.578) |  | 0.198 (-0.004, 0.400) |
|  | RESP Edu1 | 0.209 (-0.641, 1.059) |  | -0.396 (-1.223, 0.431) |
|  | RESP Edu2 | 0.153 (-0.378, 0.685) |  | -0.091 (-0.919, 0.738) |
|  | RESP Edu3 | -0.126 (-0.238, -0.014) |  | 0.198 (0.064, 0.332) |
|  | COPD Edu1 | 0.236 (-0.418, 0.891) |  | -0.618 (-2.577, 1.340) |
|  | COPD Edu2 | -0.134 (-0.260, -0.008) |  | 0.202 (0.054, 0.350) |
|  | COPD Edu3 | 0.236 (-0.488, 0.960) |  | 0.055 (-1.318, 1.428) |
|  | Nonaccidental male | 0.095 (0.043, 0.148) |  | 0.089 (0.031, 0.146) |
|  | Nonaccidental female | 0.143 (0.087, 0.198) |  | 0.102 (0.033, 0.171) |
|  | CVD male | 0.143 (0.057, 0.230) |  | 0.019 (-0.076, 0.113) |
|  | CVD female | 0.232 (0.148, 0.316) |  | 0.028 (-0.075, 0.130) |
|  | Stroke male | 0.119 (0.010, 0.229) |  | 0.044 (-0.076, 0.165) |
|  | Stroke female | 0.183 (0.074, 0.292) |  | 0.093 (-0.045, 0.230) |
|  | IHD male | 0.201 (0.037, 0.365) |  | -0.075 (-0.247, 0.098) |
|  | IHD female | 0.306 (0.155, 0.456) |  | -0.084 (-0.259, 0.091) |
|  | MI male | 0.191 (-0.013, 0.396) |  | -0.130 (-0.332, 0.072) |
|  | MI female | 0.279 (0.083, 0.475) |  | -0.088 (-0.299, 0.123) |
|  | RESP male | 0.041 (-0.081, 0.163) |  | 0.176 (0.006, 0.345) |
|  | RESP female | 0.109 (-0.010, 0.229) |  | 0.143 (-0.058, 0.344) |
|  | COPD male | 0.039 (-0.091, 0.169) |  | 0.218 (0.029, 0.407) |
|  | COPD female | 0.084 (-0.045, 0.214) |  | 0.215 (-0.008, 0.437) |
| Middle | Total | 0.088 (0.054, 0.122) |  | 0.436 (0.383, 0.490) |
|  | Non-accidental | 0.084 (0.049, 0.119) |  | 0.369 (0.314, 0.424) |
|  | Stroke | 0.065 (-0.006, 0.136) |  | 0.422 (0.300, 0.544) |
|  | CVD | 0.094 (0.038, 0.149) |  | 0.476 (0.381, 0.571) |
|  | RESP | 0.077 (-0.004, 0.158) |  | 0.510 (0.344, 0.677) |
|  | IHD | 0.150 (0.053, 0.247) |  | 0.699 (0.522, 0.876) |
|  | MI | 0.061 (-0.104, 0.225) |  | 0.605 (0.351, 0.859) |
|  | COPD | 0.093 (0.007, 0.179) |  | 0.562 (0.382, 0.741) |
|  | Nonaccidental 0to64 | 0.064 (-0.033, 0.161) |  | 0.230 (0.104, 0.357) |
|  | Nonaccidental 65to74 | 0.025 (-0.065, 0.115) |  | 0.233 (0.114, 0.351) |
|  | Nonaccidental 75above | 0.102 (0.061, 0.144) |  | 0.441 (0.371, 0.511) |
|  | RESP 0to64 | 0.017 (-0.257, 0.292) |  | 0.466 (-0.009, 0.942) |
|  | RESP 65to74 | 0.127 (-0.082, 0.335) |  | 0.566 (0.157, 0.974) |
|  | RESP 75above | 0.077 (-0.017, 0.170) |  | 0.516 (0.320, 0.712) |
|  | CVD 0to64 | 0.076 (-0.084, 0.236) |  | 0.511 (0.265, 0.756) |
|  | CVD 65to74 | 0.058 (-0.088, 0.205) |  | 0.396 (0.171, 0.621) |
|  | CVD 75above | 0.103 (0.038, 0.168) |  | 0.489 (0.375, 0.603) |
|  | Total 0to64 | 0.072 (-0.021, 0.164) |  | 0.291 (0.172, 0.409) |
|  | Total 65to74 | 0.026 (-0.061, 0.112) |  | 0.312 (0.196, 0.428) |
|  | Total 75above | 0.107 (0.067, 0.147) |  | 0.509 (0.440, 0.578) |
|  | IHD 0to64 | 0.237 (-0.020, 0.494) |  | 0.460 (0.025, 0.895) |
|  | IHD 65to74 | 0.109 (-0.151, 0.370) |  | 0.751 (0.336, 1.167) |
|  | IHD 75above | 0.146 (0.032, 0.261) |  | 0.733 (0.517, 0.948) |
|  | MI 0to64 | 0.261 (-0.120, 0.641) |  | 0.058 (-0.530, 0.646) |
|  | MI 65to74 | 0.158 (-0.230, 0.547) |  | 0.567 (-0.020, 1.154) |
|  | MI 75above | -0.001 (-0.205, 0.203) |  | 0.727 (0.409, 1.045) |
|  | Stroke 0to64 | 0.018 (-0.195, 0.231) |  | 0.569 (0.248, 0.890) |
|  | Stroke 65to74 | 0.047 (-0.142, 0.235) |  | 0.335 (0.045, 0.626) |
|  | Stroke 75above | 0.075 (-0.007, 0.157) |  | 0.421 (0.274, 0.568) |
|  | COPD 0to64 | 0.162 (-0.107, 0.432) |  | 0.601 (0.126, 1.077) |
|  | COPD 65to74 | 0.094 (-0.136, 0.324) |  | 0.662 (0.229, 1.096) |
|  | COPD 75above | 0.092 (-0.006, 0.190) |  | 0.549 (0.335, 0.763) |
|  | Nonaccidental Edu1 | 0.179 (-0.447, 0.804) |  | -0.302 (-0.906, 0.302) |
|  | Nonaccidental Edu2 | -0.123 (-0.463, 0.217) |  | 0.167 (-0.224, 0.559) |
|  | Nonaccidental Edu3 | 0.017 (-0.048, 0.082) |  | 0.094 (0.008, 0.179) |
|  | CVD Edu1 | 0.201 (-0.839, 1.241) |  | 0.263 (-0.937, 1.464) |
|  | CVD Edu2 | -0.166 (-0.691, 0.358) |  | -0.181 (-0.949, 0.587) |
|  | CVD Edu3 | 0.049 (-0.052, 0.151) |  | -0.061 (-0.205, 0.083) |
|  | Stroke Edu1 | 0.501 (-1.359, 2.361) |  | 0.867 (-0.025, 1.760) |
|  | Stroke Edu2 | -0.741 (-1.715, 0.233) |  | -0.273 (-1.386, 0.839) |
|  | Stroke Edu3 | 0.063 (-0.076, 0.202) |  | -0.105 (-0.303, 0.094) |
|  | IHD Edu1 | -0.290 (-1.822, 1.241) |  | 0.561 (-1.564, 2.686) |
|  | IHD Edu2 | 0.011 (-0.757, 0.779) |  | 0.361 (-0.472, 1.193) |
|  | IHD Edu3 | 0.031 (-0.161, 0.223) |  | -0.073 (-0.338, 0.193) |
|  | MI Edu1 | 0.014 (-1.925, 1.952) |  | 1.181 (-0.723, 3.085) |
|  | MI Edu2 | 0.023 (-0.782, 0.828) |  | -0.251 (-1.603, 1.102) |
|  | MI Edu3 | 0.049 (-0.174, 0.272) |  | -0.063 (-0.437, 0.312) |
|  | RESP Edu1 | -0.287 (-2.410, 1.836) |  | -1.251 (-3.354, 0.852) |
|  | RESP Edu2 | -0.160 (-1.519, 1.198) |  | 0.630 (-0.232, 1.492) |
|  | RESP Edu3 | -0.062 (-0.234, 0.110) |  | 0.298 (0.087, 0.509) |
|  | COPD Edu1 | -2.768 (-8.381, 2.845) |  | -2.749 (-8.298, 2.800) |
|  | COPD Edu2 | -0.029 (-0.215, 0.157) |  | 0.354 (0.121, 0.588) |
|  | COPD Edu3 | -1.075 (-3.832, 1.682) |  | 0.464 (-1.400, 2.328) |
|  | Nonaccidental male | 0.068 (0.020, 0.116) |  | 0.316 (0.245, 0.388) |
|  | Nonaccidental female | 0.103 (0.052, 0.155) |  | 0.426 (0.342, 0.510) |
|  | CVD male | 0.094 (0.016, 0.172) |  | 0.475 (0.345, 0.606) |
|  | CVD female | 0.094 (0.014, 0.173) |  | 0.473 (0.336, 0.610) |
|  | Stroke male | 0.057 (-0.043, 0.156) |  | 0.442 (0.276, 0.608) |
|  | Stroke female | 0.074 (-0.027, 0.176) |  | 0.401 (0.221, 0.580) |
|  | IHD male | 0.163 (0.025, 0.302) |  | 0.664 (0.417, 0.911) |
|  | IHD female | 0.136 (-0.001, 0.272) |  | 0.725 (0.473, 0.977) |
|  | MI male | 0.166 (-0.063, 0.396) |  | 0.654 (0.296, 1.013) |
|  | MI female | -0.050 (-0.287, 0.187) |  | 0.550 (0.192, 0.907) |
|  | RESP male | 0.036 (-0.072, 0.143) |  | 0.441 (0.227, 0.656) |
|  | RESP female | 0.137 (0.013, 0.261) |  | 0.597 (0.335, 0.858) |
|  | COPD male | 0.072 (-0.039, 0.182) |  | 0.467 (0.243, 0.690) |
|  | COPD female | 0.126 (-0.009, 0.261) |  | 0.685 (0.387, 0.982) |

*Edu1 refers to college; Edu2 refers to high school; Edu3 refers to middle school and lower

Table S8 Sensitive analysis of the interactions between temperature extremes and PM_2.5_ pollution using different extreme temperature and PM_2.5_ pollution thresholds in 3 regions of Jiangsu, 2015 to 2019

| Analysis | Region | Outcome | RERI (95% CI) |
| --- | --- | --- | --- |
| PM2.5_35_TEMP_5TH | Southern | Total | 0.052 (0.015, 0.089) |
|  |  | Non-accidental | 0.040 (0.001, 0.079) |
|  |  | Stroke | 0.020 (-0.063, 0.103) |
|  |  | CVD | 0.003 (-0.062, 0.069) |
|  |  | RESP | 0.048 (-0.072, 0.168) |
|  |  | IHD | -0.130 (-0.275, 0.016) |
|  |  | MI | -0.016 (-0.251, 0.220) |
|  |  | COPD | 0.055 (-0.095, 0.205) |
|  | Northern | Total | 0.121 (0.084, 0.158) |
|  |  | Non-accidental | 0.124 (0.085, 0.162) |
|  |  | Stroke | 0.160 (0.081, 0.239) |
|  |  | CVD | 0.213 (0.151, 0.275) |
|  |  | RESP | 0.063 (-0.032, 0.158) |
|  |  | IHD | 0.306 (0.191, 0.421) |
|  |  | MI | 0.299 (0.155, 0.443) |
|  |  | COPD | 0.047 (-0.055, 0.148) |
|  | Middle | Total | 0.068 (0.032, 0.105) |
|  |  | Non-accidental | 0.063 (0.026, 0.101) |
|  |  | Stroke | 0.033 (-0.045, 0.112) |
|  |  | CVD | 0.072 (0.011, 0.134) |
|  |  | RESP | 0.026 (-0.072, 0.124) |
|  |  | IHD | 0.137 (0.029, 0.246) |
|  |  | MI | 0.039 (-0.138, 0.217) |
|  |  | COPD | 0.031 (-0.074, 0.136) |
| PM2.5_35_TEMP_95TH | Southern | Total | 0.240 (0.128, 0.351) |
|  |  | Non-accidental | 0.204 (0.093, 0.315) |
|  |  | Stroke | 0.303 (0.149, 0.457) |
|  |  | CVD | 0.282 (0.142, 0.423) |
|  |  | RESP | 0.300 (0.097, 0.504) |
|  |  | IHD | 0.164 (-0.032, 0.361) |
|  |  | MI | 0.214 (-0.064, 0.491) |
|  |  | COPD | 0.349 (0.123, 0.574) |
|  | Northern | Total | 0.146 (0.011, 0.281) |
|  |  | Non-accidental | 0.137 (0.002, 0.271) |
|  |  | Stroke | 0.134 (-0.023, 0.290) |
|  |  | CVD | 0.101 (-0.068, 0.271) |
|  |  | RESP | 0.226 (-0.019, 0.471) |
|  |  | IHD | 0.018 (-0.236, 0.271) |
|  |  | MI | -0.004 (-0.298, 0.290) |
|  |  | COPD | 0.276 (-0.004, 0.555) |
|  | Middle | Total | 0.615 (0.437, 0.793) |
|  |  | Non-accidental | 0.498 (0.330, 0.667) |
|  |  | Stroke | 0.638 (0.415, 0.861) |
|  |  | CVD | 0.758 (0.559, 0.956) |
|  |  | RESP | 0.696 (0.395, 0.996) |
|  |  | IHD | 1.130 (0.852, 1.409) |
|  |  | MI | 1.113 (0.805, 1.422) |
|  |  | COPD | 0.768 (0.449, 1.087) |
| PM2.5_75%_TEMP_5TH | Southern | Total | 0.057 (-0.067, 0.182) |
|  |  | Non-accidental | 0.053 (-0.074, 0.179) |
|  |  | Stroke | 0.077 (-0.080, 0.234) |
|  |  | CVD | 0.066 (-0.080, 0.211) |
|  |  | RESP | 0.017 (-0.196, 0.229) |
|  |  | IHD | 0.011 (-0.181, 0.203) |
|  |  | MI | -0.064 (-0.328, 0.201) |
|  |  | COPD | 0.010 (-0.211, 0.232) |
|  | Northern | Total | -0.017 (-0.183, 0.149) |
|  |  | Non-accidental | -0.014 (-0.182, 0.153) |
|  |  | Stroke | -0.006 (-0.189, 0.177) |
|  |  | CVD | -0.061 (-0.273, 0.151) |
|  |  | RESP | 0.032 (-0.249, 0.313) |
|  |  | IHD | -0.115 (-0.434, 0.204) |
|  |  | MI | -0.239 (-0.603, 0.125) |
|  |  | COPD | -0.008 (-0.317, 0.302) |
|  | Middle | Total | 0.105 (-0.083, 0.292) |
|  |  | Non-accidental | 0.098 (-0.089, 0.286) |
|  |  | Stroke | 0.093 (-0.121, 0.307) |
|  |  | CVD | 0.080 (-0.112, 0.273) |
|  |  | RESP | 0.149 (-0.149, 0.447) |
|  |  | IHD | 0.120 (-0.122, 0.363) |
|  |  | MI | 0.139 (-0.113, 0.391) |
|  |  | COPD | 0.154 (-0.161, 0.468) |
| PM2.5_50%_TEMP_2.5TH | Southern | Total | 0.049 (-0.204, 0.301) |
|  |  | Non-accidental | 0.044 (-0.213, 0.302) |
|  |  | Stroke | 0.038 (-0.283, 0.360) |
|  |  | CVD | 0.109 (-0.198, 0.417) |
|  |  | RESP | 0.049 (-0.388, 0.486) |
|  |  | IHD | 0.234 (-0.209, 0.678) |
|  |  | MI | 0.067 (-0.515, 0.649) |
|  |  | COPD | -0.021 (-0.484, 0.441) |
|  | Northern | Total | 0.121 (-0.027, 0.269) |
|  |  | Non-accidental | 0.112 (-0.037, 0.261) |
|  |  | Stroke | 0.098 (-0.066, 0.263) |
|  |  | CVD | 0.133 (-0.061, 0.327) |
|  |  | RESP | 0.148 (-0.097, 0.393) |
|  |  | IHD | 0.222 (-0.078, 0.522) |
|  |  | MI | 0.259 (-0.099, 0.617) |
|  |  | COPD | 0.108 (-0.164, 0.379) |
|  | Middle | Total | -0.006 (-0.243, 0.231) |
|  |  | Non-accidental | -0.017 (-0.254, 0.220) |
|  |  | Stroke | -0.010 (-0.280, 0.261) |
|  |  | CVD | 0.019 (-0.227, 0.264) |
|  |  | RESP | -0.079 (-0.439, 0.281) |
|  |  | IHD | 0.041 (-0.265, 0.346) |
|  |  | MI | 0.043 (-0.276, 0.363) |
|  |  | COPD | -0.047 (-0.428, 0.333) |
| PM2.5_50%_TEMP_97.5TH | Southern | Total | 0.252 (0.135, 0.369) |
|  |  | Non-accidental | 0.222 (0.105, 0.339) |
|  |  | Stroke | 0.313 (0.153, 0.473) |
|  |  | CVD | 0.282 (0.135, 0.429) |
|  |  | RESP | 0.281 (0.065, 0.497) |
|  |  | IHD | 0.118 (-0.092, 0.328) |
|  |  | MI | 0.218 (-0.072, 0.509) |
|  |  | COPD | 0.276 (0.039, 0.513) |
|  | Northern | Total | 0.397 (0.090, 0.705) |
|  |  | Non-accidental | 0.358 (0.054, 0.662) |
|  |  | Stroke | 0.390 (0.029, 0.750) |
|  |  | CVD | 0.370 (-0.017, 0.757) |
|  |  | RESP | 0.624 (0.066, 1.183) |
|  |  | IHD | 0.222 (-0.351, 0.794) |
|  |  | MI | 0.271 (-0.376, 0.917) |
|  |  | COPD | 0.622 (-0.027, 1.270) |
|  | Middle | Total | 0.870 (0.561, 1.179) |
|  |  | Non-accidental | 0.690 (0.393, 0.986) |
|  |  | Stroke | 0.934 (0.535, 1.333) |
|  |  | CVD | 1.022 (0.670, 1.374) |
|  |  | RESP | 0.834 (0.309, 1.360) |
|  |  | IHD | 1.456 (0.993, 1.919) |
|  |  | MI | 1.610 (1.101, 2.119) |
|  |  | COPD | 0.988 (0.452, 1.523) |
| PM2.5_75%_TEMP_95TH | Southern | Total | 0.276 (0.153, 0.400) |
|  |  | Non-accidental | 0.226 (0.104, 0.349) |
|  |  | Stroke | 0.315 (0.144, 0.487) |
|  |  | CVD | 0.316 (0.159, 0.472) |
|  |  | RESP | 0.297 (0.070, 0.523) |
|  |  | IHD | 0.212 (-0.003, 0.428) |
|  |  | MI | 0.253 (-0.052, 0.559) |
|  |  | COPD | 0.310 (0.060, 0.559) |
|  | Northern | Total | 0.152 (-0.007, 0.312) |
|  |  | Non-accidental | 0.150 (-0.008, 0.309) |
|  |  | Stroke | 0.152 (-0.032, 0.336) |
|  |  | CVD | 0.149 (-0.049, 0.348) |
|  |  | RESP | 0.262 (-0.030, 0.555) |
|  |  | IHD | 0.150 (-0.146, 0.446) |
|  |  | MI | 0.182 (-0.161, 0.526) |
|  |  | COPD | 0.318 (-0.018, 0.655) |
|  | Middle | Total | 0.641 (0.433, 0.849) |
|  |  | Non-accidental | 0.496 (0.302, 0.689) |
|  |  | Stroke | 0.682 (0.422, 0.942) |
|  |  | CVD | 0.824 (0.589, 1.058) |
|  |  | RESP | 0.642 (0.292, 0.991) |
|  |  | IHD | 1.184 (0.840, 1.528) |
|  |  | MI | 1.328 (0.947, 1.710) |
|  |  | COPD | 0.726 (0.350, 1.102) |
| PM2.5_35_TEMP_2.5TH | Southern | Total | 0.081 (-0.080, 0.242) |
|  |  | Non-accidental | 0.076 (-0.088, 0.239) |
|  |  | Stroke | 0.096 (-0.106, 0.298) |
|  |  | CVD | 0.090 (-0.098, 0.279) |
|  |  | RESP | 0.077 (-0.204, 0.358) |
|  |  | IHD | 0.045 (-0.210, 0.301) |
|  |  | MI | 0.090 (-0.269, 0.448) |
|  |  | COPD | 0.013 (-0.289, 0.315) |
|  | Northern | Total | 0.116 (-0.068, 0.300) |
|  |  | Non-accidental | 0.119 (-0.067, 0.305) |
|  |  | Stroke | 0.146 (-0.062, 0.353) |
|  |  | CVD | 0.224 (-0.019, 0.466) |
|  |  | RESP | 0.028 (-0.275, 0.332) |
|  |  | IHD | 0.381 (0.015, 0.746) |
|  |  | MI | 0.390 (-0.044, 0.823) |
|  |  | COPD | 0.015 (-0.323, 0.353) |
|  | Middle | Total | 0.035 (-0.176, 0.246) |
|  |  | Non-accidental | 0.033 (-0.178, 0.244) |
|  |  | Stroke | 0.002 (-0.238, 0.243) |
|  |  | CVD | 0.040 (-0.175, 0.256) |
|  |  | RESP | 0.046 (-0.281, 0.374) |
|  |  | IHD | 0.086 (-0.178, 0.350) |
|  |  | MI | 0.059 (-0.220, 0.338) |
|  |  | COPD | 0.029 (-0.315, 0.373) |
| PM2.5_35_TEMP_97.5TH | Southern | Total | 0.232 (0.083, 0.381) |
|  |  | Non-accidental | 0.181 (0.033, 0.330) |
|  |  | Stroke | 0.342 (0.134, 0.549) |
|  |  | CVD | 0.332 (0.143, 0.520) |
|  |  | RESP | 0.322 (0.057, 0.586) |
|  |  | IHD | 0.202 (-0.060, 0.465) |
|  |  | MI | 0.352 (-0.020, 0.724) |
|  |  | COPD | 0.301 (0.012, 0.590) |
|  | Northern | Total | 0.108 (-0.082, 0.297) |
|  |  | Non-accidental | 0.095 (-0.093, 0.282) |
|  |  | Stroke | 0.079 (-0.140, 0.297) |
|  |  | CVD | -0.009 (-0.244, 0.225) |
|  |  | RESP | 0.217 (-0.135, 0.569) |
|  |  | IHD | -0.194 (-0.540, 0.152) |
|  |  | MI | -0.249 (-0.645, 0.146) |
|  |  | COPD | 0.278 (-0.126, 0.681) |
|  | Middle | Total | 0.787 (0.534, 1.040) |
|  |  | Non-accidental | 0.618 (0.380, 0.857) |
|  |  | Stroke | 0.757 (0.430, 1.084) |
|  |  | CVD | 0.935 (0.648, 1.222) |
|  |  | RESP | 0.819 (0.397, 1.242) |
|  |  | IHD | 1.464 (1.065, 1.863) |
|  |  | MI | 1.562 (1.123, 2.001) |
|  |  | COPD | 0.912 (0.467, 1.358) |
| PM2.5_50%_TEMP_5TH | Southern | Total | 0.039 (-0.074, 0.151) |
|  |  | Non-accidental | 0.032 (-0.082, 0.146) |
|  |  | Stroke | 0.049 (-0.093, 0.190) |
|  |  | CVD | 0.031 (-0.101, 0.163) |
|  |  | RESP | 0.002 (-0.190, 0.194) |
|  |  | IHD | -0.023 (-0.201, 0.155) |
|  |  | MI | -0.059 (-0.307, 0.190) |
|  |  | COPD | 0.023 (-0.179, 0.226) |
|  | Northern | Total | 0.047 (-0.090, 0.184) |
|  |  | Non-accidental | 0.038 (-0.101, 0.176) |
|  |  | Stroke | 0.017 (-0.135, 0.169) |
|  |  | CVD | 0.027 (-0.151, 0.206) |
|  |  | RESP | 0.083 (-0.144, 0.311) |
|  |  | IHD | 0.068 (-0.208, 0.343) |
|  |  | MI | 0.075 (-0.252, 0.401) |
|  |  | COPD | 0.024 (-0.227, 0.275) |
|  | Middle | Total | 0.057 (-0.096, 0.209) |
|  |  | Non-accidental | 0.048 (-0.105, 0.200) |
|  |  | Stroke | 0.057 (-0.117, 0.230) |
|  |  | CVD | 0.054 (-0.103, 0.212) |
|  |  | RESP | 0.066 (-0.174, 0.306) |
|  |  | IHD | 0.106 (-0.091, 0.303) |
|  |  | MI | 0.083 (-0.126, 0.291) |
|  |  | COPD | 0.083 (-0.168, 0.335) |
| PM2.5_50%_TEMP_95TH | Southern | Total | 0.224 (0.112, 0.335) |
|  |  | Non-accidental | 0.195 (0.084, 0.307) |
|  |  | Stroke | 0.280 (0.128, 0.432) |
|  |  | CVD | 0.249 (0.109, 0.388) |
|  |  | RESP | 0.263 (0.059, 0.467) |
|  |  | IHD | 0.102 (-0.097, 0.300) |
|  |  | MI | 0.191 (-0.085, 0.467) |
|  |  | COPD | 0.279 (0.054, 0.504) |
|  | Northern | Total | 0.200 (0.061, 0.339) |
|  |  | Non-accidental | 0.187 (0.048, 0.325) |
|  |  | Stroke | 0.216 (0.055, 0.376) |
|  |  | CVD | 0.196 (0.020, 0.371) |
|  |  | RESP | 0.255 (0.007, 0.504) |
|  |  | IHD | 0.119 (-0.147, 0.385) |
|  |  | MI | 0.078 (-0.232, 0.388) |
|  |  | COPD | 0.284 (0.002, 0.567) |
|  | Middle | Total | 0.608 (0.432, 0.784) |
|  |  | Non-accidental | 0.506 (0.339, 0.674) |
|  |  | Stroke | 0.706 (0.487, 0.924) |
|  |  | CVD | 0.764 (0.568, 0.959) |
|  |  | RESP | 0.673 (0.377, 0.969) |
|  |  | IHD | 1.039 (0.766, 1.311) |
|  |  | MI | 1.012 (0.708, 1.316) |
|  |  | COPD | 0.729 (0.416, 1.041) |
| PM2.5_75%_TEMP_2.5TH | Southern | Total | 0.003 (-0.164, 0.170) |
|  |  | Non-accidental | 0.008 (-0.163, 0.178) |
|  |  | Stroke | 0.053 (-0.160, 0.265) |
|  |  | CVD | 0.074 (-0.125, 0.273) |
|  |  | RESP | 0.018 (-0.269, 0.305) |
|  |  | IHD | 0.184 (-0.086, 0.453) |
|  |  | MI | 0.105 (-0.268, 0.478) |
|  |  | COPD | -0.050 (-0.351, 0.251) |
|  | Northern | Total | 0.077 (-0.151, 0.305) |
|  |  | Non-accidental | 0.071 (-0.159, 0.300) |
|  |  | Stroke | 0.090 (-0.162, 0.342) |
|  |  | CVD | 0.103 (-0.192, 0.398) |
|  |  | RESP | -0.048 (-0.423, 0.327) |
|  |  | IHD | 0.182 (-0.269, 0.632) |
|  |  | MI | 0.068 (-0.445, 0.582) |
|  |  | COPD | -0.130 (-0.538, 0.277) |
|  | Middle | Total | 0.126 (-0.124, 0.376) |
|  |  | Non-accidental | 0.118 (-0.131, 0.368) |
|  |  | Stroke | 0.068 (-0.215, 0.350) |
|  |  | CVD | 0.096 (-0.160, 0.351) |
|  |  | RESP | 0.151 (-0.242, 0.543) |
|  |  | IHD | 0.208 (-0.120, 0.536) |
|  |  | MI | 0.328 (-0.016, 0.672) |
|  |  | COPD | 0.135 (-0.278, 0.549) |
| PM2.5_75%_TEMP_97.5TH | Southern | Total | 0.266 (0.108, 0.423) |
|  |  | Non-accidental | 0.210 (0.054, 0.367) |
|  |  | Stroke | 0.336 (0.114, 0.557) |
|  |  | CVD | 0.330 (0.130, 0.531) |
|  |  | RESP | 0.272 (-0.011, 0.554) |
|  |  | IHD | 0.166 (-0.109, 0.441) |
|  |  | MI | 0.240 (-0.154, 0.633) |
|  |  | COPD | 0.251 (-0.055, 0.557) |
|  | Northern | Total | 0.259 (0.027, 0.491) |
|  |  | Non-accidental | 0.252 (0.022, 0.482) |
|  |  | Stroke | 0.252 (-0.015, 0.519) |
|  |  | CVD | 0.209 (-0.077, 0.494) |
|  |  | RESP | 0.531 (0.087, 0.974) |
|  |  | IHD | 0.114 (-0.301, 0.530) |
|  |  | MI | 0.101 (-0.373, 0.575) |
|  |  | COPD | 0.595 (0.084, 1.106) |
|  | Middle | Total | 0.507 (0.237, 0.776) |
|  |  | Non-accidental | 0.348 (0.098, 0.597) |
|  |  | Stroke | 0.474 (0.132, 0.817) |
|  |  | CVD | 0.633 (0.326, 0.939) |
|  |  | RESP | 0.401 (-0.044, 0.846) |
|  |  | IHD | 0.981 (0.535, 1.427) |
|  |  | MI | 1.340 (0.844, 1.836) |
|  |  | COPD | 0.473 (-0.004, 0.949) |
